# Metamaterial Antennas Enhance MRI of the Eye and Occipital Brain

**DOI:** 10.1002/adma.202517760

**Published:** 2026-02-02

**Authors:** Nandita Saha, Bilguun Nurzed, Mostafa Berangi, Andre Kuehne, Helmar Waiczies, Igor Fabian Tellez Ceja, Xiang Hu, Thomas Gladytz, Lisa Krenz, Dave Huebler, Beate Endemann, Claudia Brockmann, Ebba Beller, Oliver Stachs, Thoralf Niendorf

**Affiliations:** ^1^ Max‐Delbrück‐Center for Molecular Medicine in the Helmholtz Association (MDC) Berlin Ultrahigh Field Facility (B.U.F.F.) Berlin Germany; ^2^ Charité—Universitätsmedizin Berlin Experimental and Clinical Research Center (ECRC) A Joint Cooperation between the Charité Medical Faculty and the Max‐Delbrück Center for Molecular Medicine in the Helmholtz Association Berlin Germany; ^3^ Faculty V, Medical Engineering Technische Universität Berlin Berlin Germany; ^4^ Faculty II Berliner Hochschule für Technik Berlin Germany; ^5^ Magforce NT GmbH Berlin Germany; ^6^ MRI.TOOLS GmbH Berlin Germany; ^7^ Department of Ophthalmology Rostock University Medical Center Rostock Germany; ^8^ Institute of Diagnostic and Interventional Radiology, Pediatric Radiology and Neuroradiology Rostock University Medical Center Rostock Germany; ^9^ Department Life, Light & Matter University of Rostock Rostock Germany

**Keywords:** clinical translation, epsilon‐negative unit cell, eye and orbit, metamaterials, MRI, occipital brain, radiofrequency antennas, split‐ring resonators

## Abstract

A metamaterial‐integrated radio frequency antenna (MTMA), implemented in planar and bend configurations, enables high‐resolution MRI of the eye, orbit, and occipital brain at 7.0 T. Its dual‐layer co‐planar architecture integrates a two‐channel transceive loop with a metamaterial layer composed of subwavelength epsilon‐negative unit cells. These unit cells were custom‐designed based on classical split‐ring resonators for operation at 7.0 T. Electromagnetic simulations, including human voxel models, guided the design and characterization of the MTMA's electromagnetic behavior. Both MTMA configurations were benchmarked against conventional loop coil arrays in phantoms and in vivo for experimental validation, demonstrating enhanced transmit (B_1_^+^) efficiency and receive sensitivity enabled by the metamaterial layer through resonant near‐field coupling. MRI safety was verified through SAR simulations, bio‐thermal modeling, Magnetic Resonance thermometry, and fiber‐optic sensors, confirming compliance with safety guidelines. The Bend‐MTMA enabled in vivo human MRI of the eye and orbit in healthy volunteers, including B_1_^+^ mapping, and provided diagnostic T_1_‐ and T_2_‐weighted imaging in volunteers with retinal pathology and sinus cysts, demonstrating clinical applicability. The Planar‐MTMA enabled occipital lobe MRI in human volunteers, achieving superior signal coverage and transmit performance. The modular unit cell design enables tuning across MRI magnetic field strengths, establishing a clinically translatable metamaterial‐integrated antenna platform for ocular and neurological imaging.

## Graphical abstract

A radiofrequency antenna platform comprising planar and bend configurations is developed, incorporating structurally integrated epsilon‐negative metamaterial unit cells to enhance MRI. These antennas enable high‐resolution in vivo human MRI of the eye, orbit, and occipital brain. Comprehensive validation, including simulations, phantom experiments, SAR, MR thermometry, B₁^+^ mapping, and in vivo MRI in healthy volunteers and one volunteer with retinal pathology, demonstrates clinical feasibility.

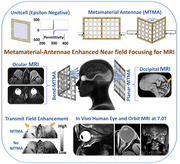

## 1 Introduction

Magnetic resonance imaging (MRI) is a mainstay of diagnostic imaging [1, 2]. A growing number of reports document technical innovations specifically in MRI of the eye and orbit and promote their application in translational research and clinical diagnostics [3]. The delicate morphology of the eye and orbit requires imaging with sub‐millimeter spatial resolution, whereas MRI's susceptibility to bulk motion necessitates short acquisition times [3]. Seizing this opportunity, new research directions and emerging clinical applications of ocular MRI are enabled by the sensitivity gains and spatial resolution enhancements afforded by high‐field (B_0_≥3.0 T) and ultrahigh‐field (UHF, B_0_≥7.0 T) MRI [4, 5, 6, 7, 8, 9]. Emerging applications of UHF‐MRI of the eye and orbit include diagnostic confirmation of ambiguous ophthalmoscopic findings such as retinal detachment, visualization and local staging of ocular masses (e.g. uveal melanoma, retinoblastoma) with direct relevance for treatment planning and follow‐up, 3D MRI for improved ocular biometry, fusion with color Doppler ultrasound for the assessment of choroidal melanoma and optic nerve disorders or physio‐metabolic imaging probing iron concentration or water diffusion [4, 5, 6, 7, 8, 9, 10, 11].

Despite this progress, the potential of UHF‐MRI of the eye and orbit is yet untapped, and the advantages are sometimes offset by a number of concomitant physics effects that bear the potential to spoil the benefits of UHF‐MRI [12]. The radiofrequency (RF) wavelength shortening at UHFs cause transmission field inhomogeneities, which may impair image quality due to shading and even signal voids. RF power deposition and specific absorption rate (SAR) increase with higher RF frequency and tissue conductivity at UHF. This physics phenomenon induces local tissue heating and may constitute safety concerns for imaging highly conductive organs like the eye [3, 12]. Dedicated RF coil arrays with independent phase setting for each element for excitation transmit field modulation have been explored to enhance the image quality in UHF‐MRI of the eye and orbit [11, 12, 13]. These RF technologies have been synergistically supported by the introduction of parallel RF transmission (pTx) techniques and by high‐permittivity dielectric pads [14, 15, 16, 17]. However, the latter remain constrained by geometry conditions, bulky design, and difficulty in integration with an anatomically adaptive RF coil [17, 18]. Another aspect of RF coil development is parallel imaging, which facilitates acceleration of MRI to reduce scan times by utilizing spatial sensitivity across phased‐array RF coils [19, 20, 21, 22, 23, 24, 25, 26, 27, 28, 29, 30, 31]. Because parallel imaging inherently reduces signal‐to‐noise ratio (SNR), advanced RF coil development would help to enhance performance in accelerated imaging.

Electromagnetic (EM) metamaterials (MTMs) present a viable alternative to dielectric pads and offer a transformative solution to the persistent challenges of UHF MRI by enhancing transmit and receive efficiency through tailored RF field shaping [17, 18, 21, 26, 27, 32]. MTMs are artificial media composed of periodic subwavelength unit cells (UCs), engineered to control effective permittivity (ε_eff_) and permeability (µ_eff_), thereby enabling manipulation of electromagnetic fields [33, 34]. Their electromagnetic behavior arises from geometry rather than chemical composition. MTMs can exhibit epsilon‐negative (ε_eff_ < 0, ENG), mu‐negative (µ_eff_ < 0, MNG), or double‐negative (ε_eff_ < 0, µ_eff_ < 0, DNG) behavior, allowing diverse EM‐field control and enabling unconventional wave phenomena such as negative refraction, reversed Doppler effect, and superlensing [33, 34, 35, 36, 37, 38]. Pendry et al. first demonstrated DNG media using periodic arrays of split‐ring resonators (SRRs), subwavelength UC structures [36, 37]. An SRR consists of two concentric rings with gaps and acts as an LC resonator [36, 39, 40, 41, 42, 43]. When arranged periodically, they produce magnetic or electric responses depending on geometry and excitation [43]. SRRs, widely used in optics and microwave, for example, in superlensing, antenna miniaturization, and gain enhancement, also show promise in MRI for RF field shaping to improve imaging and safety [37, 43, 44, 45].

Prior MRI studies have explored flexible thin or bulk dielectric‐based MTM surfaces as passive add‐ons to conventional RF coils to improve receive sensitivity, but few have pursued true structural integration [15, 16, 17, 18, 26, 32, 46, 47, 48, 49, 50, 51, 52, 53, 54, 55, 56, 57, 58, 59, 60, 61, 62, 63, 64, 65, 66, 67, 68, 69, 70, 71, 72, 73, 74, 75]. Unlike add‐on metamaterial surfaces used in conjunction with a conventional RF coil, metamaterial‐loaded antennas can be directly implemented as a single integrated resonant structure to reshape transmit–receive fields via resonant or non‐resonant near‐field coupling [76, 77, 78, 79]. Seizing this opportunity, we developed a multi‐channel transceive metamaterial antenna (MTMA) for 7.0 T MRI. It was implemented in planar and bend configurations for neuro‐ and ocular imaging, with each design embedding an MTM layer into a 2‐channel loop array using a coplanar dual‐layer approach. The MTM layer consists of a periodic array of custom‐designed subwavelength SRR UCs, structurally distinct from classical SRRs and engineered to exhibit epsilon‐negative (ENG) behavior with electric‐dipole resonance at the 7.0 T Larmor frequency. This metamaterial‐integrated antenna enhances transmit–receive performance through near‐field coupling, effectively transforming a conventional loop array into a structurally unified MTM‐enhanced antenna that functions as a unified resonant system.

We present a comprehensive design and validation pipeline encompassing electromagnetic field simulations, phantom experiments, thermal safety assessments using SAR and magnetic resonance thermometry (MRTh), Transmit B_1_^+^ field mapping, and multi‐tissue‐contrast in vivo imaging, benchmarked against conventional 2‐channel loop RF coils and with an add‐on MTM configuration. We showed that the MTMA consistently outperforms both reference configurations in transmit–receive efficiency and anatomical coverage, highlighting the contribution of the integrated MTM layer. The bend MTMA was evaluated and applied for imaging the eye, optic nerve, extraocular muscles, and orbit in healthy volunteers and in subjects with an intraocular tumor. The planar MTMA was used to image the occipital lobe, which includes the primary and association visual cortex, and is primarily responsible for visual processing. The proposed design and validation framework serve as a translational roadmap for metamaterial‐integrated next‐generation clinical RF antenna configurations.

## 2 Results

### 2.1 Metamaterial Unit Cell Design and Characterization

#### 2.1.1 Unit Cell Design

**FIGURE 1:**
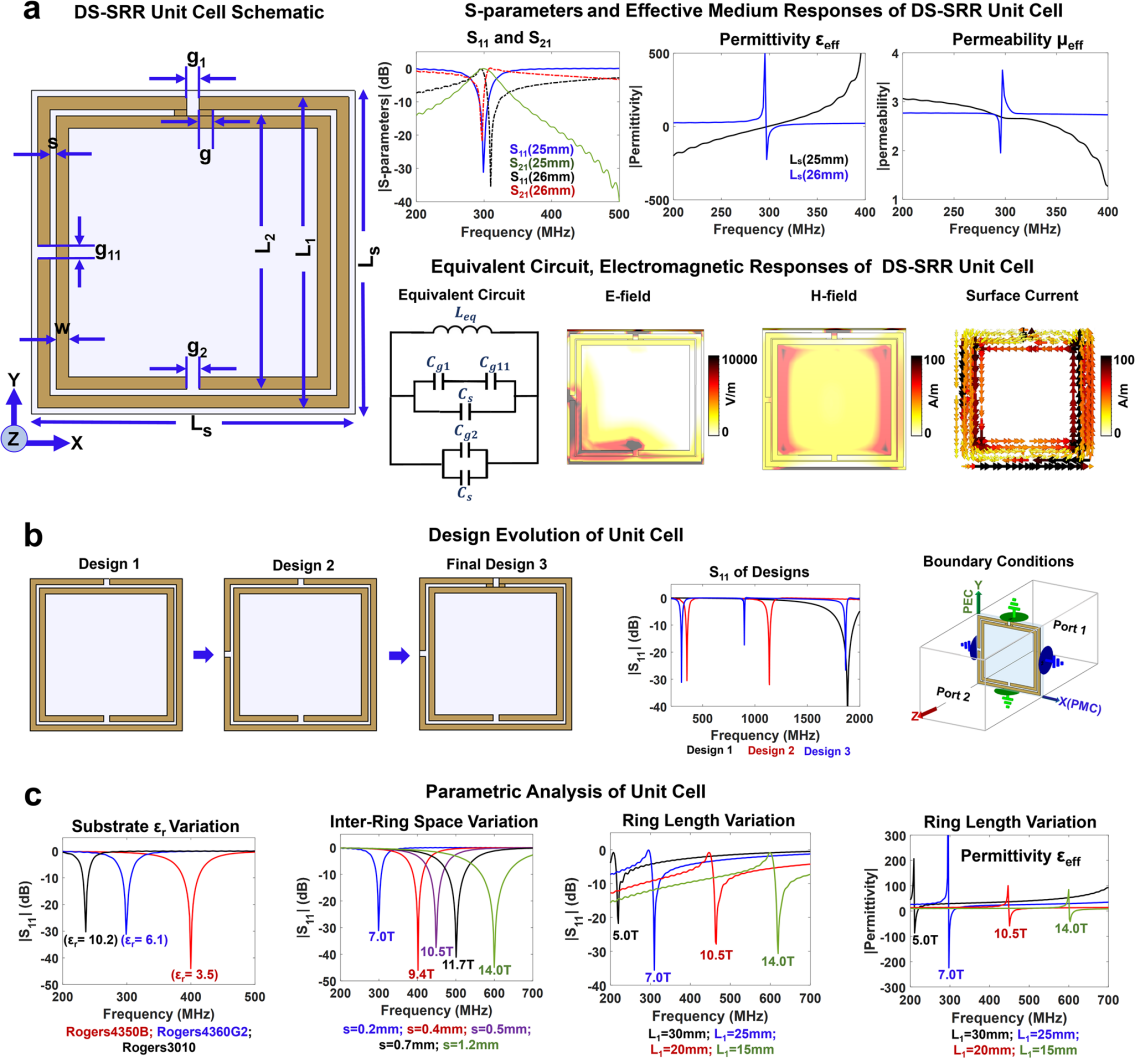
(a) Double‐square split‐ring resonator (DS‐SRR) unit cell schematic with its S‐parameters, effective permittivity (ε_eff_) and permeability (µ_eff_) responses, equivalent circuit model, electromagnetic field distributions (E‐ and H‐fields), and surface current. (b) Geometric evolution of the unit cell designs: Design 1 (basic SRR), Design 2 (intermediate), and Design 3 (final DS‐SRR), along with the corresponding S₁₁ and boundary‐condition setup. **(c)** S₁₁ responses from the unit cell's parametric analysis of substrate ε_r_, inter‐ring spacing s, and ring length L_1_ variations, together with the corresponding effective permittivity ε_eff_ response for the L_1_ variation.

In this study, we designed a subwavelength SRR‐based metamaterial unit cell (UC) that is structurally distinct from classical SRRs. The UC was modeled using annealed copper (thickness = 35 µm, conductivity = 5.96 × 10^7^ S/m), on a Rogers 4360G2 substrate (dielectric constant ε_r_ = 6.15, thickness = 1.52 mm) to enable miniaturization and ensure low dielectric loss and mechanical robustness [80]. The UC's physical size is 26 × 26 × 1.52 mm^3^, corresponding to an electrical dimension of 0.026λ × 0.026λ at 7.0 T Larmor frequency ≈297.2 MHz, where wavelength λ = 1 m in free space (Figure 1a). At 297.2 MHz, the UC's side length of 26 mm corresponds to λ/38 in free space and approximately λ/15 in the Rogers 4360G2 substrate, thereby satisfying subwavelength conditions in both media. The UC structure is based on a double‐square split‐ring resonator (DS‐SRR), comprising two concentric square rings on the dielectric substrate [39, 40, 41, 42]. The outer ring (Ring 1) includes an additional split gap on its left arm, while two copper strips interconnect it with the inner ring (Ring 2), as given in Figure 1a of the UC schematic. These features increase electrical length, enhancing inductive response and capacitive coupling. This enables tuning of the resonance frequency while maintaining compactness. Ring 1 and Ring 2 have side lengths of L_1_  =  25 mm and L_2_  =  22.6 mm, respectively, with uniform copper trace width (w  = 1 mm) and split gap (g = g_1_ = g_2_  =  1 mm), the inner ring length L_2_ is updated automatically according to the relation L_2_ = L_1_−2w−2s. Both rings have diametrically opposite gaps, and Ring 1's additional sidearm gap (g = g_11_) is also of the same size (Figure 1a). The inter‐ring spacing between Ring 1 and Ring 2 is s  =  0.2 mm. Two substrate sizes were studied: L_s _ =  25 mm (same as L_1_) and L_s _ =  26 mm (extended).

Initial UC parameters, including ring lengths, gaps, widths, and inter‐ring spacing, were estimated using an analytical LC equivalent circuit model (Figure 1a) of the DS‐SRR that incorporated geometric inductance and capacitance modeling, yielding a resonance frequency of ≈294.8 MHz, close to 297.2 MHz [39, 40, 41, 42, 43, 44].

The resonance frequency is calculated by

(1) $$f=\frac{1}{2\pi \sqrt{\left(L_{eq}\cdot C_{eq}\right)}}$$

where the equivalent inductance L_eq_ was taken as the sum of the inductances of the two rings and the metal strips, which were estimated using closed‐form expressions for rectangular loop conductors [39, 81].

(2) $$L_{eq}=L_{1}+L_{2}+L_{strip}$$

and the equivalent capacitance C_eq_ included both the split‐gap capacitances (C_g_) and the inter‐ring coupling capacitance (C_s_)

(3) $$C_{eq}=\left(\frac{3C_{g}}{2}\right)+2C_{s}$$

where C_g_ and C_s_ were approximated using parallel‐plate capacitor models on the Rogers 4360G2 substrate [39, 40, 41, 42, 43, 44]. The complete analytical model derivation is provided in Supporting Information S1.

In parallel with the analytical model, simulation‐based design refinement progressed from a conventional SRR (Design 1) to the final DS‐SRR (Design 3), as illustrated in Figure 1b by the geometric progression achieved through optimization of width, gap, and inter‐ring spacing. UC's EM simulations were done in CST Studio Suite's time‐domain solver with hexahedral meshing based on the Finite Integration Technique. The UC was simulated between two waveguide ports along the z‐axis, with PEC on the y‐axis and PMC on the x‐axis, following common boundary conditions to excite electric resonance in SRR‐based metamaterials (Figure 1b) [39, 40, 41, 42, 43, 44, 82, 83, 84, 85]. Applied PEC boundaries across the SRR gap (y‐axis) enforce electric field polarization and induce voltage‐driven circulating currents, triggering LC resonance. A PMC boundary along the SRR arms (x‐axis) preserves symmetry and enables full‐mode excitation by eliminating tangential electric fields [82, 83, 84, 85]. This setup effectively excites the electric dipole mode while minimizing unwanted magnetic coupling [82, 83, 84, 85].

The effective medium ratio (EMR), a key criterion for validating the subwavelength behavior of metamaterial UCs, was also computed:

(4) $$EMR=\frac{wavelength\;in\left(mm\right)}{UC\;length\;in\left(mm\right)}$$

where, EMR > 4 is generally considered subwavelength behavior [82, 83, 84, 85].

#### 2.1.2 S‐Parameters of the Unit Cell

Figure 1a shows the DS‐SRR UC (L_s_ = 26 mm) and its simulated S‐parameters (scattering parameters) at 297.2 MHz. The final DS‐SRR UC design (Design 3) was simulated with two substrate lengths: L_s_ = 25 mm (same as L_1_), which showed S₁₁ (reflection coefficient or return loss) and S_21_ (transmission coefficient) minima at 297.2 MHz. In contrast, L_s_ = 26 mm (extended) exhibited an S_21_ minimum at a lower frequency than S₁₁, indicating modified coupling and phase behavior (Figure 1a, **S_11_** and **S_21_**). The extended substrate alters near‐field boundary conditions, increasing capacitive loading and elongating the current path. The final design (L_s_ = 26 mm) retained the analytical model geometry (S1 Supporting Information), satisfied subwavelength (well below the ≈λ/15 threshold) criteria with an EMR of ≈38 at 297.2 MHz, and was used for all subsequent simulations and systems integration. Figure 1b illustrates the simulated S₁₁ for the geometric progression of the DS‐SRR UC from the initial design (Design 1) to the final configuration (Design 3), where the S₁₁ shows that the resonance was shifted from approximately ≈1.5 GHz (Design 1) to 297.2 MHz (Design 3).

#### 2.1.3 Effective Medium Characterization and Electromagnetic Field Distributions

The DS‐SRR UC (Ls = 26 mm) exhibited effective negative permittivity, i.e., epsilon‐negative (ENG) behavior centered around 297.2 MHz (Figure 1a,**ε_eff_**). This response originates from strong E‐field confinement at the capacitive gaps and circulating surface currents along the SRR arms, which are characteristic of electric dipole resonance and consistent with the observed S‐parameter shift. In contrast, the real part of permeability (µ_eff_ ≈ 2.2) remained positive and weakly dispersive (Figure 1a **,µ_eff_**) [82, 83]. This ENG UC supports subwavelength resonance and localized electromagnetic field confinement, enabling controlled electromagnetic field shaping. When the UCs are arranged into an array to form the MTM layer, these ENG UCs facilitate transmit–receive field shaping in MRI. Electromagnetic field analysis of the DS‐SRR UC at 297.2 MHz revealed strong E‐field localization at the capacitive split gaps, consistent with electric dipole excitation (Figure 1a, **E‐field**). The H‐field intensity was highest along the vertical DS‐SRR arms, with lower magnitude near the top and bottom arms adjacent to the gaps (Figure 1a, **H‐field**). Surface currents formed a circulating loop along the DS‐SRR arms, with the strongest current density concentrated on the vertical arms, which in turn produced the higher H‐field intensity observed in these regions (Figure 1a, **surface current**). Together, the gap‐focused E‐field and this surface current pattern confirm that the dominant resonance mode is electric‐dipole driven [82, 83, 84, 85].

#### 2.1.4 Parametric Analysis of the Unit Cell

To evaluate tunability and geometric sensitivity, a series of parametric simulations was performed to investigate how variations in the UC geometry influence its resonance behavior [82, 83, 84, 85]. The DS‐SRR was simulated on three commercial Rogers substrates, RO3010 (ε_r_ = 10.2), RO4360G2 (ε_r_ = 6.1), and RO4350B (ε_r_ = 3.5), to assess the effect of substrate permittivity on the resonance frequency. The inter‐ring spacing ‘s’ was additionally varied on the RO4360G2 substrate while all other geometric parameters were kept fixed. To explore tunability across MRI magnetic field strengths, the UC's size, i.e., the ring length L_1_, was varied (15, 20, 25, 30 mm) with all other parameters fixed. Small adjustments to the inter‐ring spacing (s = 0.19, 0.20, 0.11, 0.08 mm) were applied to align each UC geometry with representative MRI field‐strength operating frequencies (5.0 T (210.2 MHz), 7.0 T(297.2 MHz), 10.5 T (450 MHz), 14.0 T (600 MHz)) [25, 86]. Representative S₁₁ responses for these variations are shown in Figure 1c. A comprehensive UC parametric analysis, including extended results and E–H field distributions, is provided in S4.

Parametric simulations (Figure 1c) showed that UC resonance can be tuned across MRI field strengths by adjusting substrate permittivity (ε_r_), inter‐ring spacing (s), and the UC size (L_1_). These geometric modifications enabled frequency adaptation for UHF MRI at 5.0 T, 9.4 T, 10.5 T, 11.7 T, and 14.0 T [25, 62, 86]. Increasing substrate permittivity ε_r_ lowered the resonance frequency due to increased capacitive loading, decreasing ε_r_, the resonance shifted to a higher frequency (Figure 1c **, ε_r_ variation**). Increasing the inter‐ring spacing ‘s’ reduced the effective capacitance and raised the resonance frequency, enabling fine‐tuning for a fixed substrate (Figure 1c, **s variation**). Shorter ring lengths L_1_, that is, smaller UC size, lowered the inductive and capacitive loading and shifted the resonance to a higher frequency, while larger L_1_ shifted it to a lower frequency. However, ring length did not alter the intrinsic effective medium behavior of the UC, and all configurations remained within the epsilon‐negative regime (Figure 1c, **L_1_ variation ε_eff_)**. Together with the extended parametric analysis in S4, these results show that the UC design serves as a scalable and modular electromagnetic building block, maintaining ENG behavior across geometric variations while also supporting MNG response, stable array interactions, and localized electromagnetic field shaping. It offers an adaptable platform for different MRI field strengths and enables control over H‐ and E‐field distributions, offering additional capabilities alongside standard MRI resonator designs and supporting integration into next‐generation metamaterial‐integrated RF antennas.

### 2.2 Wireless Metamaterial Surface Design, Implementation, and Characterization

**FIGURE 2:**
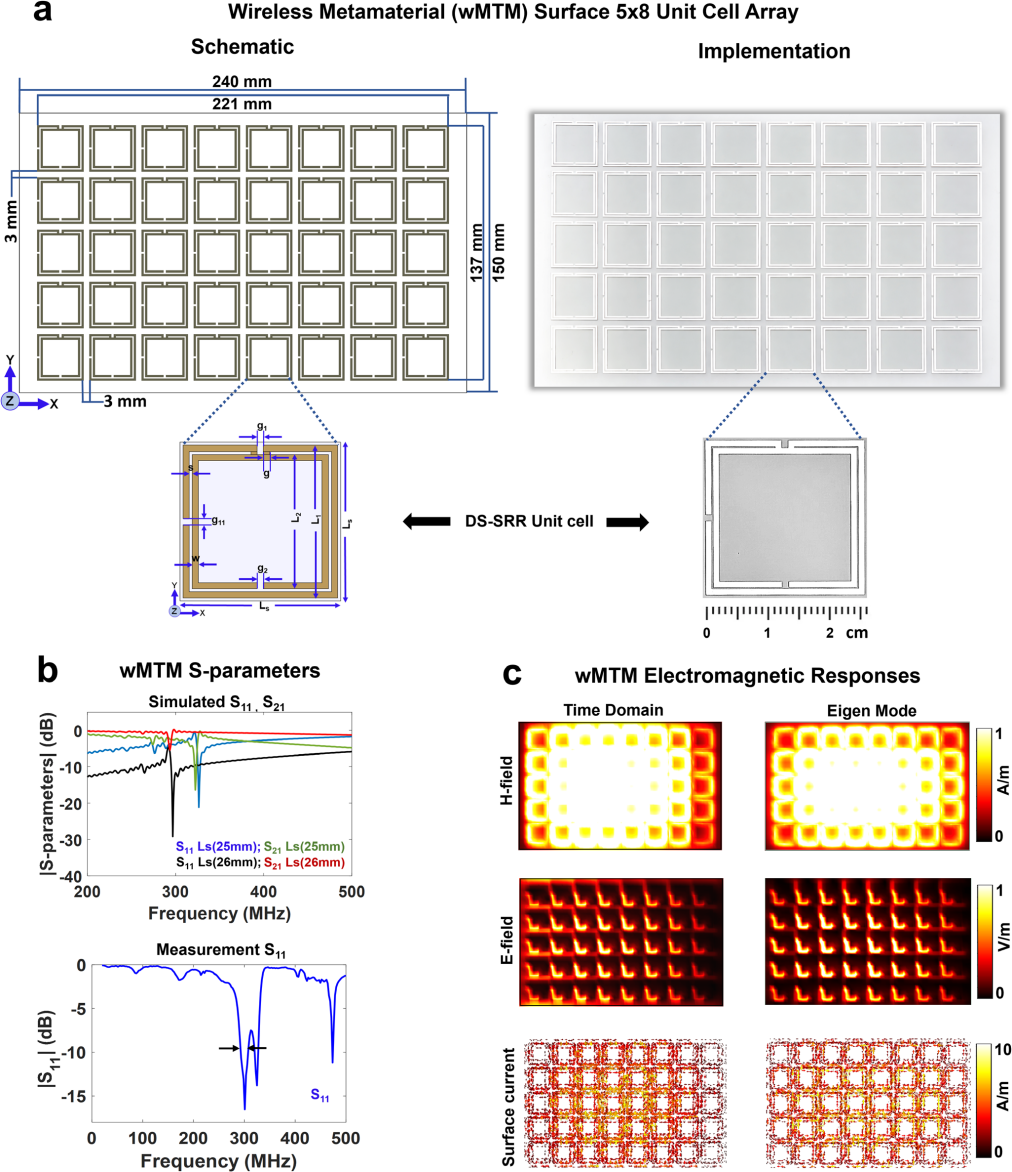
(a) Schematic layout and implemented wireless metamaterial (wMTM) surface with 5 × 8 unit cells on a Rogers RO4360G2 substrate. A zoomed view of the DS‐SRR unit cell, as seen from the wMTM, is shown. (b) Simulated S_11_ and S_21_ of the wMTM for inter‐UC space from 0.5–3.0 mm, with a 3 mm space shifting the resonance frequency to 297.2 MHz. Measured S_11_ of the implemented wMTM using a pick‐up loop coil shows a –10 dB bandwidth of 295.6–305.6 MHz, covering the 7.0 T Larmor frequency of 297.2 MHz. (c) wMTM's electromagnetic responses (E‐ and H‐ fields), and surface current distribution obtained from time‐domain and eigenmode solvers, normalized to respective peaks. An enlarged figure of surface current is given in S7.

Following the simulated UC design, we designed a metamaterial surface by arranging the UCs in a planar array, which we refer to as a wireless metamaterial (wMTM) due to its fully passive architecture, without any active components. The wMTM surface was designed in CST Studio Suite as a 5 × 8 array of UCs on a Rogers 4360G2 substrate, yielding a 240 × 150 × 1.52 mm^3^ structure tuned to 297.2 MHz (Figure 2a, **schematic)**. This UC layout, comprising 40 elements, ensured structural uniformity and subwavelength operation at 297.2 MHz. Each UC retained its original geometry, while inter‐UC spacing was varied between 0.5–3 mm to reduce coupling between adjacent UCs and preserve resonance. The simulation environment, including boundary conditions, was kept identical to that of the single‐UC simulation, using the same substrate. Based on the simulated wMTM design, the wMTM was constructed by fabricating a 5×8 array of UCs on a Rogers 4360G2 substrate, forming a 240 × 150 × 1.52 mm^3^ structure identical to the simulation design (Figure 2a, **implementation)**. Simulated S‐parameters in Figure 2b show that although the UC was designed to resonate at 297.2 MHz, integration into a 5×8 UC array altered the collective response due to mutual coupling, and with a 3 mm inter‐UC spacing, the array was retuned to 297.2 MHz. Bench measurements with a vector network analyzer (VNA) using a 10 × 10 cm^2^ square pick‐loop placed over the central region (H‐field maximum) of the wMTM yielded the S₁₁ response, with a −10 dB bandwidth spanning 293.6–305.6 MHz (Figure 2b, **measurement S_11_)**. The E–H field profiles and surface currents of the wMTM were evaluated at 297.2 MHz, and the resulting distributions matched those observed in the single‐UC simulations (Figure 2c, **Time domain)**. When individual UCs are arranged periodically to form the wMTM surface, they no longer behave as isolated resonators but instead support a collective electromagnetic response across the array. This collective response produces a broad, high‐intensity H‐field region centered on the array, with a gradual decrease toward the periphery (Figure 2c, **H‐field Time domain)**. The resulting H‐field distribution reflects the fundamental collective behavior of the dipole‐driven resonance of the individual ENG UCs. In addition, eigenmode simulations were conducted to cross‐check the resonant electromagnetic mode behavior of the wMTM, using periodic boundary conditions in all directions (Figure 2c) [48, 49, 50, 51]. Eigenmode analysis of the wMTM confirmed a self‐sustained electromagnetic mode from electric dipole resonance at ≈295 MHz, consistent with a centralized H‐field distribution in the time‐domain results and supporting its potential for spatial field control in MRI (Figure 2c, **H‐field Eigen Mode)** [51, 64].

### 2.3 Metamaterial‐Integrated Antenna and Loop Array: Design, Implementation, and Bench Validation

**FIGURE 3:**
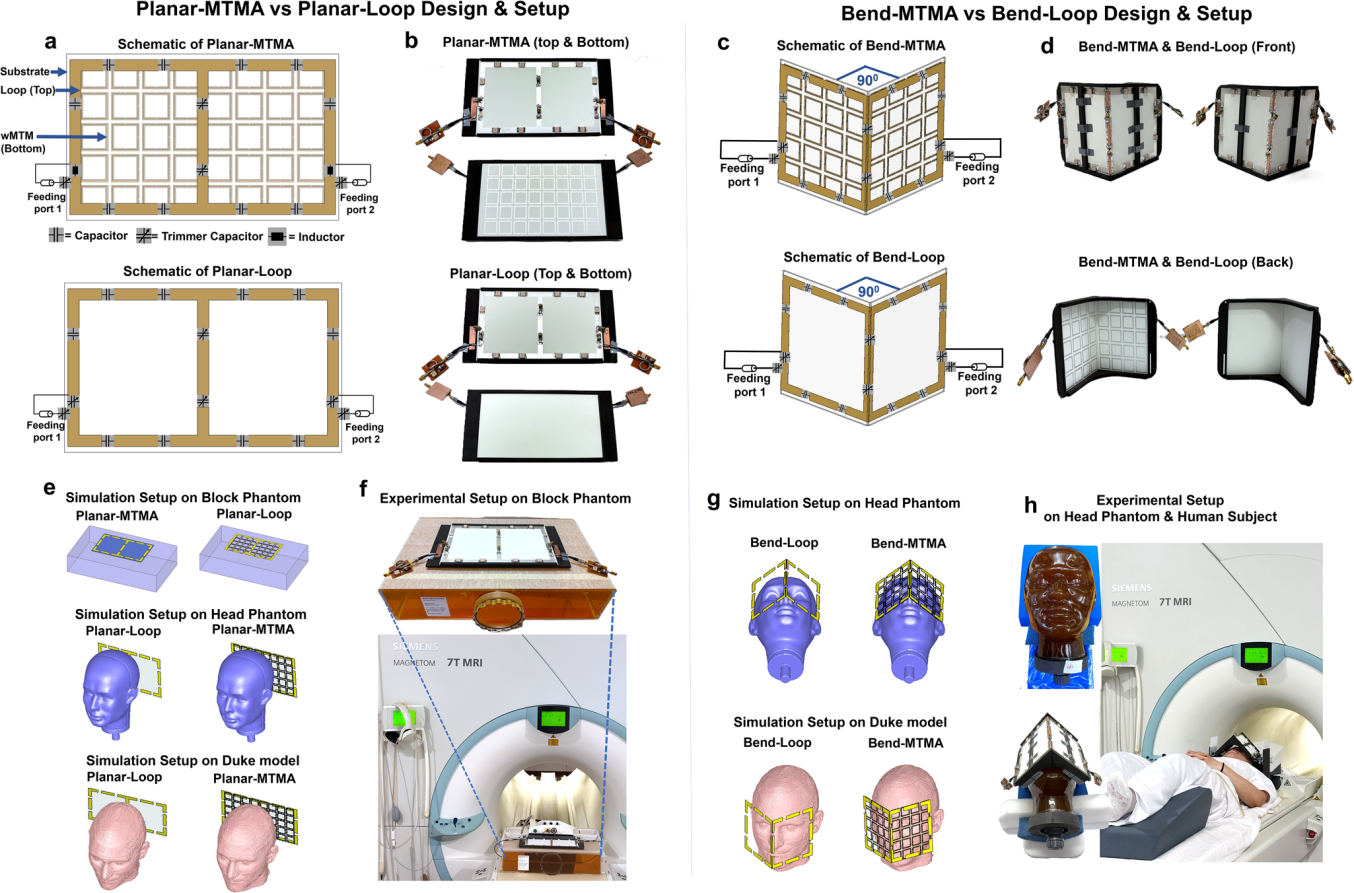
Design, fabrication, and deployment of planar and bend RF antennas with and without an integrated wireless metamaterial (wMTM) layer. (a) Schematic layout of Planar‐MTMA and Planar‐Loop with feeding ports and lumped components. (b) Planar‐MTMA and Planar‐Loop (top and bottom view) implementation. (c) Schematic layout of Bend‐MTMA and Bend‐Loop showing the 90° bend around the middle line (central axis) with feeding ports and lumped components. (d) Bend‐MTMA and Bend‐Loop (front and back view) implementation. (e) Simulation setup of Planar configurations on a rectangular phantom, positioned 1 cm away from the phantom surface (1st row), simulation setup of the posterior placement of the Planar configurations on a head phantom (2nd row), and on the human voxel model Duke (3rd row) to mimic in vivo occipital lobe imaging conditions. (f) Experimental setup on the rectangular phantom and corresponding scanner setup inside a 7.0 T whole‐body human MRI system. (g) Simulation setup showing the anterior placement of the Bend‐MTMA and Bend‐Loop on the head phantom (1st row) and on the human voxel model Duke (2nd row), mimicking in vivo ocular imaging conditions. (h) Experimental setup using an anatomically shaped head phantom. For the in vivo ocular MRI feasibility study using a 7.0 T whole body human MRI system, the Bend‐MTMA was positioned on the anterior head of a human subject.

Following the development of the wMTM surface, we designed a metamaterial integrated RF antenna (MTMA) in which the wMTM is incorporated as a functional part of the antenna architecture rather than attached as a passive add‐on (Figure 3a–d). Two MTMA configurations were designed and simulated: (i) a planar 2‐channel metamaterial‐loaded surface loop antenna (Planar‐MTMA) in which the wMTM is incorporated as part of the antenna architecture, and (ii) a bend configuration of the planar design (Bend‐MTMA). For direct comparison of these MTMA, conventional loop counterparts without metamaterial were designed: (iii) Planar‐Loop and (iv) Bend‐Loop. All RF antennas were designed on Rogers 4360G2 substrates in simulation and subsequently implemented at a resonance frequency of 297.2 MHz (7.0 T).

#### 2.3.1 Planar Metamaterial‐Integrated RF Antenna (Planar‐MTMA)

To investigate the impact of a metamaterial‐integrated loop antenna compared with a conventional loop RF coil, we designed the Planar‐MTMA in simulations using a coplanar dual‐layer architecture, in which the wMTM surface and the 2‐channel surface RF loop array were realized on opposite sides (top and bottom layers) of the same Rogers PCB, forming a structurally unified MTM‐integrated RF antenna (Figure 3a,b) [76, 77]. The top layer of the PCB contains a 2‐channel rectangular transceive planar surface loop (size: 240 ×150 mm^2^) modeled using 1‐cm‐wide annealed copper (thickness = 35 µm, conductivity = 5.96 × 10^7^ S/m), matching the wMTM area (Figure 3a,b) [87, 88, 89, 90]. This 2‐channel loop array was constructed with 12 distributed capacitors placed along its length to ensure uniform current distribution. Tuning and impedance matching were achieved using tuning (C_t_) and matching (C_m_) capacitors. Two shared decoupling capacitors (C_d_) were placed on the common conductor between the two loops to minimize inter‐loop mutual coupling without geometric overlap [87]. The Planar‐MTMA was fed via capacitive tuning (C_t_) and matching (C_m_) networks at the two feeding ports of the top‐layer loop array. The bottom layer featured a wMTM (5 × 8 UCs), precisely aligned beneath the top‐layer loop‐array footprint to avoid edge overlap and spatial EM‐field destructive interference (Figure 3a,b). No direct electrical connection exists between the top‐layer loop‐array and the wMTM layer, aside from the shared substrate. Instead, near‐field coupling between the layers modulates both the transmit and receive fields, that is, the central H‐field of the wMTM constructively interacts with the loop‐arrays's field. This dual‐layer approach of sharing the same substrate ensured mechanical stability, reproducible alignment, and stable electromagnetic performance, thus overcoming the alignment variability typical of separately mounted metamaterial layers with an RF coil. Planar‐MTMA was tuned and impedance‐matched to 297.2 MHz.

To model near‐field interaction in the MTMA system, the active loop‐array (top layer) and passive wMTM array (bottom layer) were treated as resonant subsystems separated by a dielectric substrate (Rogers 4360G2, thickness 1.52 mm, ε_r_ ≈ 6.15) [76, 77]. The induced current in the wMTM layer was calculated by representing the MTMA as a two‐port network using a Z‐parameter formulation (see full derivation in S5):

(5) $$I_{2}=-\frac{Z_{mutual}}{Z_{2}}\cdot \frac{V_{1}}{Z_{1}-\frac{Z_{mutual}^{2}}{Z_{2}}}$$

where V_1_ is the loop port applied voltage, I_1_ is the current in the loop, and I_2_ is the induced current in the wMTM. Z_1_ and Z_2_ denote the self‐impedances of the loop and wMTM layers, while Z_mutual_ accounts for mutual coupling across the dielectric. Substrate capacitance was approximated using a parallel‐plate capacitor model of equation 6 with A is the effective overlap area of DS‐SRR UCs and loop trace, d = 1.52 mm is the substrate thickness

(6) $$C_{\mathrm{substrate}}=\frac{\varepsilon_{r}\varepsilon_{0}A}{d}$$

#### 2.3.2 Reference RF Antenna (Planar‐Loop)

In addition to the Planar‐MTMA, a conventional single‐layer transceive 2‐channel Planar‐Loop RF array was designed using identical geometry (Figure 3a,b), capacitor distribution, and substrate material, but without the wMTM layer. This configuration served as the reference RF antenna for benchmarking the Planar‐MTMA's performance. Due to slight electromagnetic loading differences between the two designs, the Planar‐Loop configuration was retuned and impedance‐matched using C_t_, C_m_, and C_d_ components to maintain resonance at 297.2 MHz.

#### 2.3.3 Bend Configurations of RF Antennas: Bend‐MTMA and Bend‐Loop

To further evaluate MTMA's performance in anatomically relevant geometries and clinically meaningful applications, we developed 90° bend versions (Bend‐MTMA and Bend‐Loop) of the core planar designs in simulation (Figure 3c,d). In the bend configuration, the PCB was split at its midpoint and reassembled into a right‐angle (90°) configuration, resembling a gable‐roof shape. This bending geometry conforms more closely to human facial anatomy, ensuring effective anatomic coverage and transmit‐receive efficiency for ophthalmic and frontal brain imaging. Each bend configuration was individually tuned and impedance‐matched to 297.2 MHz.

#### 2.3.4 Implementation and Bench Measurements of Planar and Bend Antennas

All four antenna configurations were implemented and experimentally validated through bench measurements. The Planar‐MTMA and Planar‐Loop implementations (Figure 3b), as well as the bend configurations (Figure 3d), were fabricated on Rogers 4360G2 substrate exactly as designed in the simulations. The bend configuration involved splitting the PCB at its midpoint and fixing it at a 90° angle using a custom‐built 3D‐printed casing. The distributed fixed ceramic capacitors, along with the tuning (C_t_), matching (C_m_), and decoupling (C_d_) trimmer capacitors, were soldered directly onto the loop surface for all configurations. Tuning and matching were performed using L‐section circuits using trimmer capacitors C_t_ and C_m_. S‐parameters (S_11_, S_2__2_, S_21_, S_12_) were measured at the bench for all four antenna configurations at 297.2 MHz using a vector network analyzer. Planar configurations were tested on the rectangular phantom as well as on the head phantom, and bend configurations on the head phantom. Trimmer capacitors were precisely adjusted to achieve return loss < –13 dB (S_11_/S_22_) and inter‐channel isolation < –13 dB (S_21_/S_12_). To assess in vivo loading, S‐parameters were measured in three healthy volunteers (2 males, 1 female, BMI 21–25 kg/m^2^), representing the average loading range. Detailed capacitor values for each configuration, along with all S‐parameter measurements, are presented in S3. Each antenna from the four configurations was individually connected via its two feeding ports to a 1:2 power splitter and a transmit–receive (TR) switch box (MRI.TOOLS GmbH, Berlin, Germany, Stark Contrast, Erlangen, Germany). The TR switch box interfaced the antenna with the RF chain of a whole‐body 7.0 T MRI scanner (Magnetom, Siemens Healthineers, Erlangen, Germany) (Figure 3f,h) and included the necessary PIN‐diode circuitry for switching between transmit/receive modes. All four antenna configurations were validated in phantom studies, followed by in vivo human ocular MRI using the Bend antennas and occipital brain MRI using the Planar antennas at 7.0 T.

### 2.4 Transmit and Receive Performance Evaluation of the Planar and Bend Antennas

#### 2.4.1 Phantom and Human Voxel Model Transmit Field Simulation Methods

To evaluate MRI performance, we simulated the transmit field (B₁^+^) for all four antenna configurations. For validation, the simulations were benchmarked against experimental measurements. B₁^+^ simulations were performed for two‐phase excitation modes based on antenna geometry. For the planar configurations, equal‐phase excitation (0°/0°) was applied to ensure constructive interference at the central conductor, enhancing B₁^+^ via symmetric current addition. For the bend configurations, quadrature hybrid excitation (0°/90°) matched the 90° geometry, mimicked birdcage excitation, and produced uniform circularly polarized fields [88].

The planar configurations were assessed for B₁^+^ on a homogeneous rectangular shaped block phantom (450 × 270 × 100 mm^3^, ε_r_ = 58, σ = 0.77 S/m) (Figure 3e, **first row**). Planar configurations were positioned 1 cm above the rectangular phantom surface during simulation, and comparisons included Planar‐MTMA vs. Planar‐Loop and Planar‐Loop + add‐on wMTM to distinguish the effects of integrated versus add‐on MTM structures. Planar configurations were also evaluated for B₁^+^ on an anatomically shaped head phantom (ε_r_ = 56.01, σ = 0.411 S/m) with posterior placement to reflect realistic occipital brain imaging conditions (Figure 3e, **second row**). Bend configurations were similarly evaluated for B₁^+^ with anterior placement on the head phantom to simulate ocular imaging conditions (Figure 3g, **first row**). To approximate human in vivo conditions, B₁^+^ simulations were performed using the ‘Duke’ human voxel model (IT'IS Foundation), truncated at the neck (2 × 2 × 2 mm^3^), with assigned tissue properties at 297.2 MHz (Figure 3e,g) [91, 92]. For the Duke model, the Bend configurations were placed anteriorly (centered over the eyes), and the planar configurations were positioned posteriorly (under the occipital lobe). Transmit efficiency was calculated as B₁^+^ (µT/√kW) within regions of interest (ROIs) placed in the phantom and Duke [93].

#### 2.4.2 Phantom MRI Validation Methods for Transmit Field and Receive Signal Intensity

Phantom experiments were conducted to evaluate the performance of planar and bend MTMA compared to their loop counterparts. Two phantoms, identical to those used in the simulations, were used for experimental validation to match antenna geometry and imaging targets (Figure 3f,h). The rectangular phantom (450 × 270 × 100 mm^3^), filled with water, PVP, and salt (ε_r_ = 58, σ = 0.77 S/m), was used to evaluate the performance of the planar configurations, which were positioned centrally, 1 cm above the phantom surface, to assess B₁^+^ and receive sensitivity (Figure 3f). The anatomically shaped head phantom (≈4 kg), filled with water, PVP, CuSO_4_, and salt (ε_r_ = 56.01, σ = 0.411 S/m, ρ = 1000 kg/m^3^) (MRI.TOOLS GmbH, Berlin, Germany), was used to mimic realistic anatomical conditions (Figure 3h). For occipital lobe imaging, the planar configurations were placed at the back of the head phantom and aligned with the real‐world position of the occipital lobe with 1.5 cm spacing, while the bend configurations were positioned on the anterior side, centered over the eyes, ≈1.5 cm from the forehead, and ≈5 cm from the eye surface, using custom 3D‐printed holders.

Experimental phantom measurements were performed as the simulation using the same excitation schemes, i.e., equal‐phase (0°/0°) for planar configurations and quadrature hybrid (0°/90°) for bend configurations. For each antenna geometry, Loop and MTMA comparisons used identical MRI pulse sequences and protocol parameters to assess the transmit field (B₁^+^) and receive sensitivity via low flip angle shot gradient‐echo (GRE FLASH) imaging. B₁^+^ maps were acquired under standardized conditions, using actual flip angle mapping for all four configurations, and were validated against simulations [94, 95]. Under steady‐state conditions and uniform phantom properties, measured signal intensity served as a reliable metric for relative receive sensitivity across antenna configurations [96]. Transmit B₁^+^ and receive sensitivity were evaluated in phantom experiments using mean ± SD values derived from manually defined ROIs, which were presented in bar plots, while corresponding 1D profiles were extracted along horizontal and vertical lines. Multi‐slice orientations (axial, sagittal, coronal) were used in phantom experiments to assess spatial distribution.

To further examine the compatibility of the MTMA with parallel imaging (PI), the performance of the MTMA and Loop antennas was evaluated under accelerated MRI [19, 20, 21, 22, 23, 24, 25, 26, 27, 28, 29, 30, 31]. PI scans were performed using the Siemens integrated Parallel Acquisition Techniques (iPAT) package, employing GRAPPA (Generalized Autocalibrating Partially Parallel Acquisition, k‐space–based reconstruction) and mSENSE (modified SENSE, image‐domain reconstruction) with an acceleration factor of R = 2. All remaining sequence parameters matched those of the GRE‐FLASH protocol used in the respective phantom experiments (see Protocol Table S1). Rectangular block phantom PI MRI were acquired using the Planar‐MTMA and Planar‐Loop configurations, while head phantom PI MRI were performed using the Bend‐MTMA and Bend‐Loop configurations. PI performance comparisons between the MTMA and Loop antennas are presented in detail in S6.

#### 2.4.3 Phantom MRI Validation Results for Transmit Field and Receive Signal Intensity

##### 2.4.3.1 Planar Antennas on Rectangular Phantom

**FIGURE 4:**
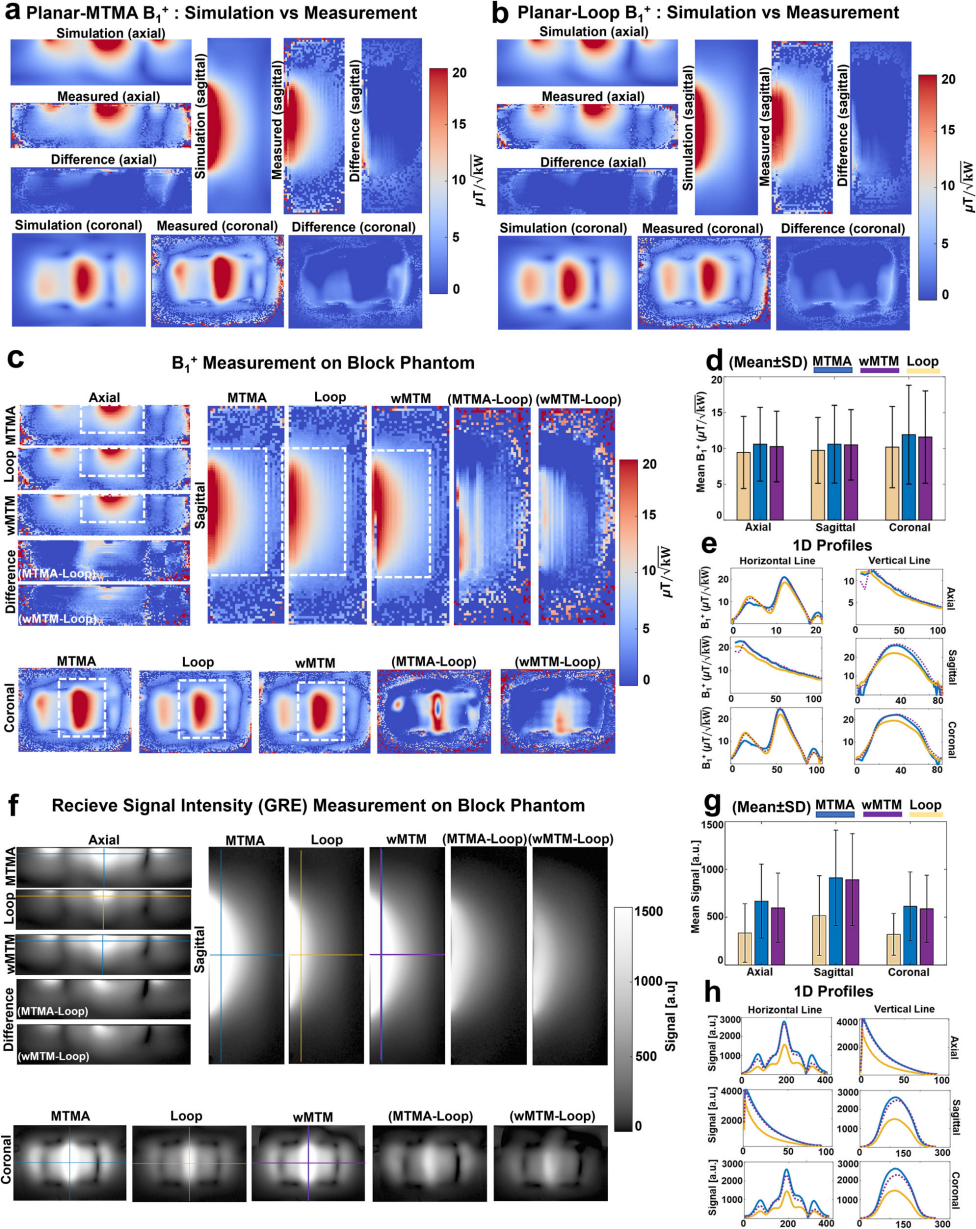
Transmit and receive performance of the Planar‐MTMA, Planar‐Loop, and Planar‐Loop+ add‐on wMTM placed on a rectangular block phantom. (a, b) Simulated and measured B₁^+^ maps, including difference maps obtained for the Planar‐MTMA and Planar‐Loop for axial (central), sagittal (central), and coronal (at ≈2 cm depth) slices. (c) Experimental transmit B₁^+^ maps and corresponding difference maps for the Planar‐MTMA, Planar‐Loop, and Planar‐Loop+wMTM configurations. The ROI (white dashed outline) used to extract mean B₁^+^. (d) Bar plots of mean B₁^+^ within the ROI. (e) 1D B₁^+^ profiles evaluated along the vertical and horizontal phantom lines (f) GRE receive signal intensity obtained for axial, sagittal, and coronal slices using the Planar‐MTMA, Planar‐Loop, and Planar‐Loop+wMTM configurations. (g) Bar plots of mean signal intensity within the same ROI used for mean B₁^+^. (h) 1D signal profiles evaluated along the vertical and horizontal phantom lines.

To assess the transmit B₁^+^ performance, Figure 4a,b compares simulated and measured B₁^+^ field maps obtained for the rectangular phantom using the Planar‐MTMA and the Planar‐Loop, showing agreement, as confirmed by the difference maps. Figure 4c shows B₁^+^ measurement maps and corresponding difference maps for the Planar‐MTMA, Planar‐Loop, and Planar‐Loop+wMTM. The Planar‐MTMA configuration demonstrated higher transmit efficiency ≈17% (axial), 14% (sagittal), and 20% (coronal) compared to the Planar‐Loop, as shown in the bar plots (Figure 4d). The Planar‐MTMA showed additional increases of ≈3% (axial), 1% (sagittal), and 3% (coronal) than Planar‐Loop+wMTM. Compared to the Planar‐Loop, the Planar‐Loop+wMTM shows ≈10% (axial), 13% (sagittal), and 16% (coronal) increased mean B_1_^+^ (Figure 4d). Both the MTMA and the wMTM add‐on setups outperformed the conventional Planar‐Loop. The 1D B₁^+^ profiles across the phantom (Figure 4e) confirmed that the MTMA and wMTM add‐on configurations produced stronger transmit fields than the Planar‐Loop.

Next, we examined the GRE receive signal intensity profiles and corresponding difference maps for the Planar‐MTMA, Planar‐Loop, and Planar‐Loop+wMTM (Figure 4f). Figure 4g shows that the Planar‐MTMA achieved a higher mean receive signal than the Planar‐Loop and the Planar‐Loop+wMTM. Estimated increases of ≈100% (axial), 77% (sagittal), and 92% (coronal) were observed compared to the Planar‐Loop, while moderate gains of ≈12%, 2%, and 5% were observed over the Planar‐Loop+wMTM (Figure 4g). Compared to the Planar‐Loop, the Planar‐Loop+wMTM shows a gain of ≈79% (axial), 73% (sagittal), and 84% (coronal). 1D signal profiles (Figure 4h) confirmed that the Planar‐MTMA yields the highest signal intensity across all orientations, consistent with the mean ROI results. The observed signal improvements extended into the phantom center, indicating enhanced receive sensitivity and greater signal reach toward deeper regions of the phantom. This reflects greater effective depth penetration of the receive field via near‐field focusing supported by the integrated wMTM layer.

Notably, the Planar‐MTMA exhibited greater signal enhancement during reception than transmission. This asymmetry aligns with MRI reciprocity: while B₁^+^ and B₁^−^ fields share spatial profiles, net receive efficiency can surpass transmit due to energy directionality and boundary conditions [97]. It is further reinforced by the spatial arrangement of the loop and wMTM layers, which favors receive‐phase coupling. In transmission, the loop array, the active source, couples its B₁^+^ field secondarily into the wMTM across the dielectric substrate. During reception, the wMTM, positioned on the imaging object‐facing side, functions as a passive electromagnetic lens that is first excited by the MR signal, captures it via resonant coupling, and forwards the B₁^−^ field into the co‐integrated loop via near‐field evanescent interaction [98, 99, 100, 101, 102, 103, 104]. At 297.2 MHz, the effective wavelength in Rogers 4360G2 (ε_r_ ≈ 6.15) is ≈40 cm, making the 1.52 mm substrate thickness deeply subwavelength (≈λ/265), where capacitive and inductive near‐field interactions dominate and induce near‐field coupling across the adjacent layer via evanescent field interactions [100, 101, 102, 103, 104]. The wMTM surface, formed by a 5×8 array of 40 UCs, supports a collective electromagnetic response characterized by a broader, centrally intensified H‐field. This collective mode couples efficiently with the primary field of the integrated loop, thereby enabling stronger signals and improved depth coverage, as achieved with the MTMA design [68, 105]. This mechanism underlies the observed co‐resonant behavior and explains the stronger receive enhancement. Equation (5) further shows that the induced current (I_2_) in the wMTM depends on both coupling impedance and impedance matching between the two layers (S5). Near resonance, where impedance conditions align, I_2_ is maximized, resulting in constructive field reinforcement and enhanced receive sensitivity, as confirmed by our experimental data.

##### 2.4.3.2 Planar Antennas on Head Phantom

**FIGURE 5:**
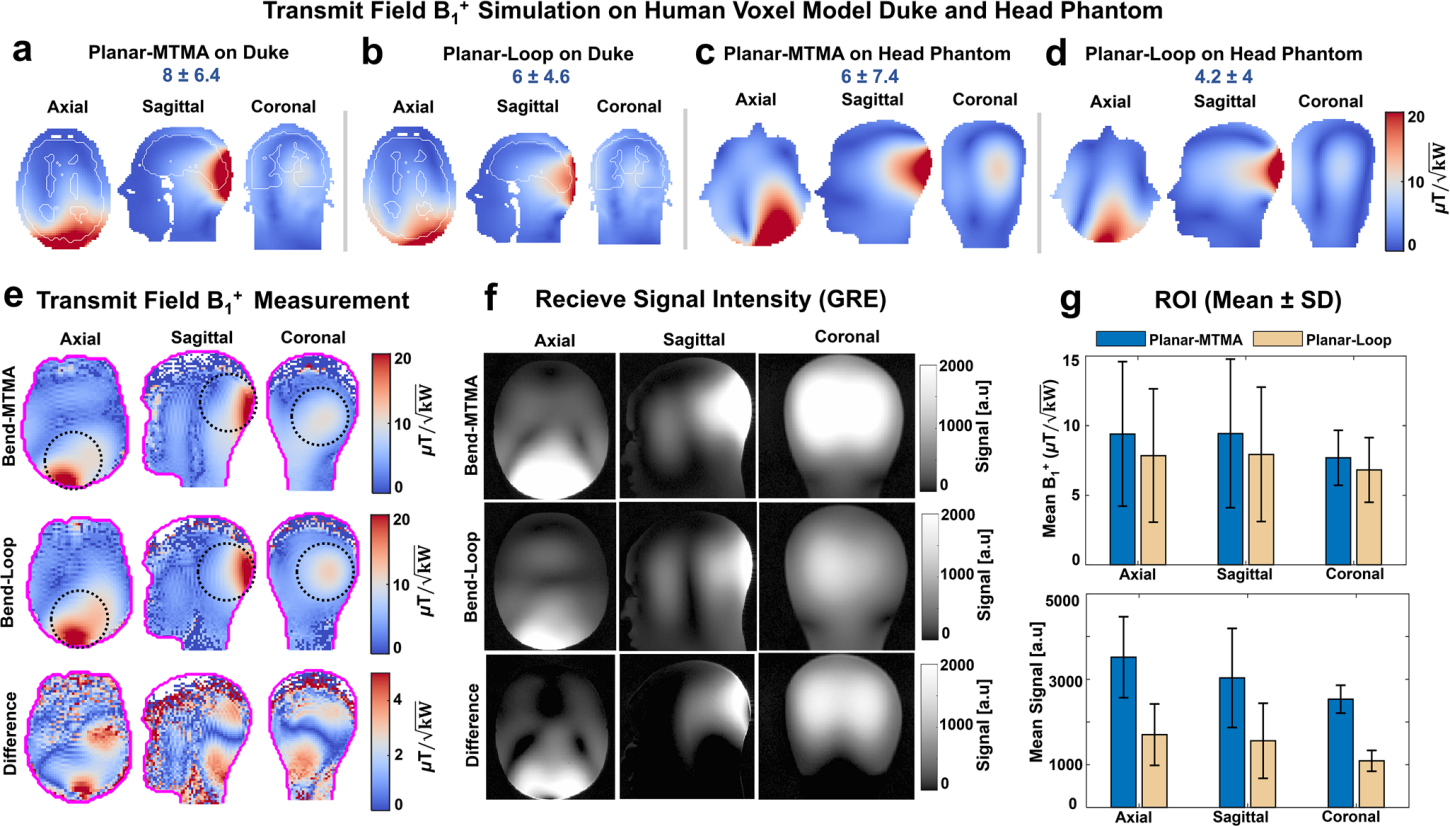
Transmit and receive performance of the Planar‐MTMA and Planar‐Loop configuration placed posteriorly on an anatomically shaped head phantom. (a, b) Simulated B₁^+^ maps obtained for the human voxel model Duke, comparing the Planar‐MTMA and Planar‐Loop. The brain ROI is highlighted with a white dashed outline, and the mean ± SD is indicated on the headers. (c, d) Simulated B₁^+^ maps comparing the Planar‐MTMA and Planar‐Loop for the head phantom, and the mean ± SD is indicated on the headers. (e) Comparison of measured B₁^+^ maps, including difference maps (MTMA‐Loop) derived experimentally from the head phantom using the Planar‐MTMA and Planar‐Loop. The ROI, delineated by a black dashed outline, was used to extract mean B₁^+^ for the bar plot. (f) Receive signal intensity images from GRE MRI of the head phantom acquired with the Planar‐MTMA and Planar‐Loop, with corresponding difference images (MTMA – Loop). The same ROI from B₁^+^ measurement was used to extract mean signal intensity [a.u.] for the bar plot. (g) The top bar plots show measured mean B₁^+^ within the circular ROI for axial, sagittal, and coronal slices. The bottom bar plots show measured mean signal intensity within the ROI.

Figure 5a–d shows simulated B₁^+^ maps of the planar configurations with posterior placement on the Duke model and the head phantom, demonstrating higher occipital B₁^+^ intensity for the Planar‐MTMA than the Planar‐Loop, with ≈33% improvement in Duke and ≈42% in the head phantom.

Experimental B₁^+^ maps shown in Figure 5e at 7.0 T confirmed this observation, with gains of ≈21% (axial), 19% (sagittal), and 13% (coronal) over the Planar‐Loop, quantified in the top bar plot of Figure 5g. GRE imaging (Figure 5f) revealed receive sensitivity improvements of 106% (axial), 94% (sagittal), and 132% (coronal), as quantified in the bottom bar plot of Figure 5g, consistent with the results from the rectangular phantom.

##### 2.4.3.3 Bend Antennas on Head Phantom

**FIGURE 6:**
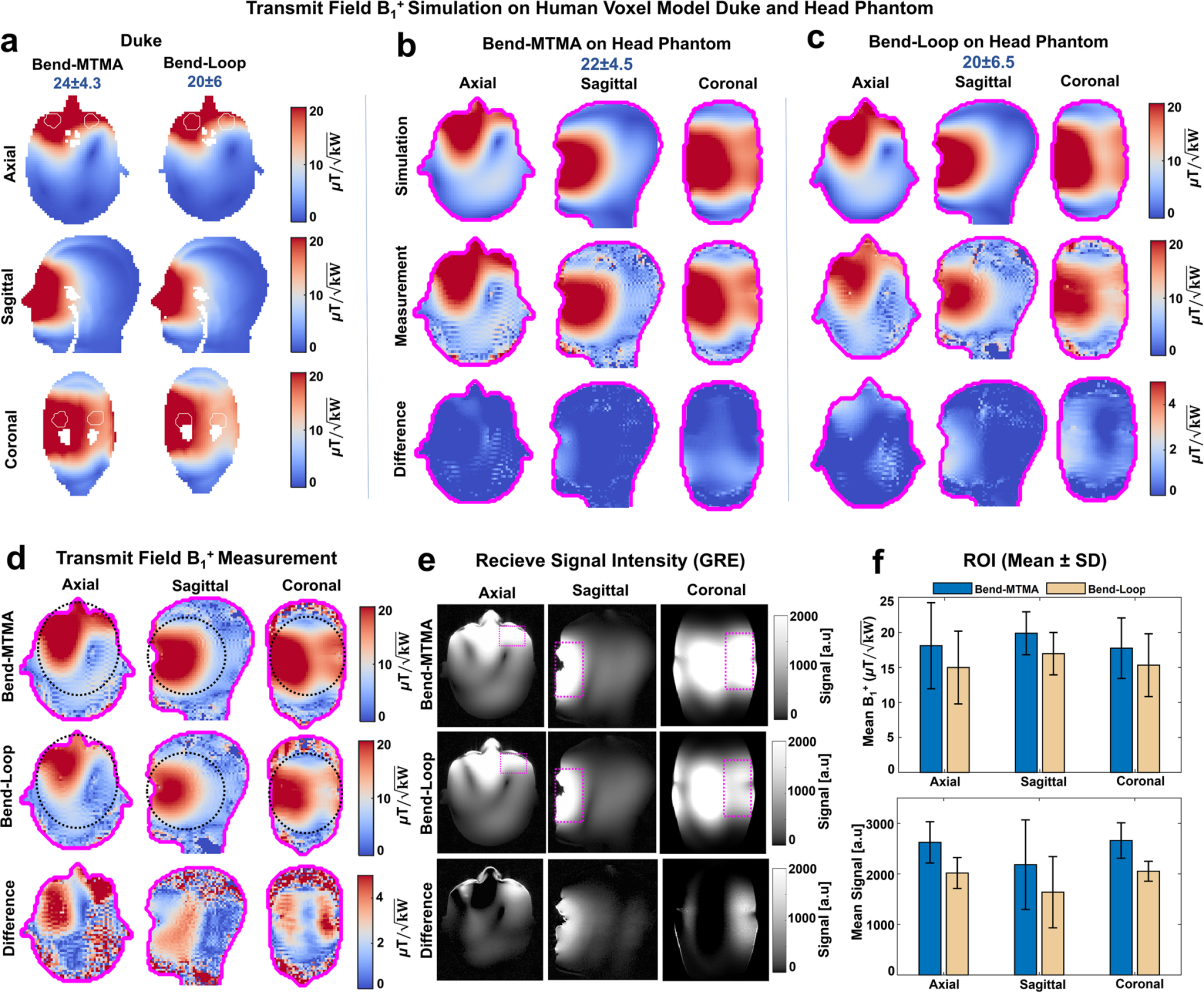
Transmit and receive performance of the Bend‐MTMA vs Bend‐Loop configuration placed on the anterior head of an anatomically shaped head phantom. (a) Simulated transmit field B₁^+^ maps using quadrature hybrid excitation of the human voxel model Duke for Bend‐MTMA and Bend‐Loop, shown for axial, sagittal, and coronal orientations. The eye ROI is highlighted with a white outline. Headers indicate mean ± SD of simulated B₁^+^ inside ROI. (b,c) Simulated and measured B₁^+^ maps obtained for the head phantom using the Bend‐MTMA and Bend‐Loop. The corresponding difference maps between simulation and experiment are shown in the third row. Headers indicate mean ± SD only for simulated B₁^+^ maps. (d) Comparison of measured B₁^+^ maps obtained from the head phantom using the Bend‐MTMA and Bend‐Loop, with corresponding difference maps (MTMA – Loop). The ROI, delineated by a black dashed outline, was used to extract mean B₁^+^ for the bar plot. (e) Receive signal intensity images obtained with GRE MRI of the same head phantom using the Bend‐MTMA and Bend‐Loop, with corresponding difference images (MTMA – Loop). The ROI, delineated by a dashed rectangular pink outline, was used to extract mean signal intensity [a.u.] for the bar plot. (f) The top bar plots show measured mean B₁^+^ within the circular ROI for axial, sagittal, and coronal slices. The bottom bar plots show the mean GRE signal intensity within the pink rectangular ROI.

The next step was to evaluate the bend antenna configurations. Simulations using this anatomically relevant setup with the Duke model (Figure 6a) demonstrated that the Bend‐MTMA provides ≈20% higher transmit B₁^+^ than the Bend‐Loop for eye ROIs. The Bend‐Loop produced an asymmetric B₁^+^ distribution between the eyes, clearly visible in the coronal slice. This asymmetric excitation pattern was effectively mitigated by the Bend‐MTMA, resulting in a more uniform transmission field. These results highlight the value of the human voxel model in predicting anatomical challenges and guiding antenna design for in vivo ocular imaging. Following the Duke simulations, B₁^+^ was assessed using the head phantom. The measured B₁^+^ field distributions closely matched the simulation results, as confirmed by the difference maps (Figure 6b,c). Experimental B₁^+^ maps shown in Figure 6d demonstrated that the Bend‐MTMA yielded transmit gains of ≈21% (axial), 18% (sagittal), and 20% (coronal) over the Bend‐Loop, quantified in the top bar plot of Figure 6f, which is consistent with the results obtained for the Duke. Figure 6e shows GRE receive signal intensity maps of the head phantom, revealing that the Bend‐MTMA outperformed the Bend‐Loop, with enhancements of ≈30%, 33%, and 30% across axial, sagittal, and coronal views, quantified in the bottom bar plot of Figure 6f. These results demonstrate the Bend‐MTMA's superior transmit–receive performance over the Bend‐Loop.

Although the received signal gains obtained for the Bend‐MTMA were less pronounced than for the Planar‐MTMA, the gains remain substantial and anatomically meaningful, particularly in the eye and orbit region. This reduction arises from the forehead‐mounted Bend‐MTMA placement, which introduced a ≈ 5 cm antenna‐to‐eye separation due to facial curvature and foam padding, limiting near‐field coupling to the wMTM layer. In contrast, the planar setup allowed ≈ 1 cm uniform spacing between the antenna and the flat rectangular phantom, which favors stronger coupling. Despite the increased antenna‐to‐object distance, the wMTM layer maintains its near‐field focusing under curvature. As a result, the Bend‐MTMA mitigates the asymmetric excitation pattern clearly visible in the coronal slice with the Bend‐Loop and provides a more uniform transmit field (Figure 6d,e). The overall gain obtained with the Bend‐MTMA therefore reflects the wMTM layer's improved signal coverage under anatomically curved loading conditions, reinforcing its suitability for ocular MRI.

##### 2.4.3.4 Parallel Imaging Performance of Planar and Bend Antennas

MRI images acquired with the MTMA were reliably reconstructed under both GRAPPA and mSENSE at R = 2 and preserved the antenna's higher receive sensitivity compared with the Loop in both planar and bend configurations. PI‐reconstruction–related artifact patterns remained comparable between antennas. These findings further support the MTMA's ability to maintain receive sensitivity and stable reconstruction performance under accelerated imaging conditions. Detailed PI results are provided in S6.

### 2.5 SAR and Temperature Simulation with Thermal Safety Validation

#### 2.5.1 SAR Simulations

**FIGURE 7:**
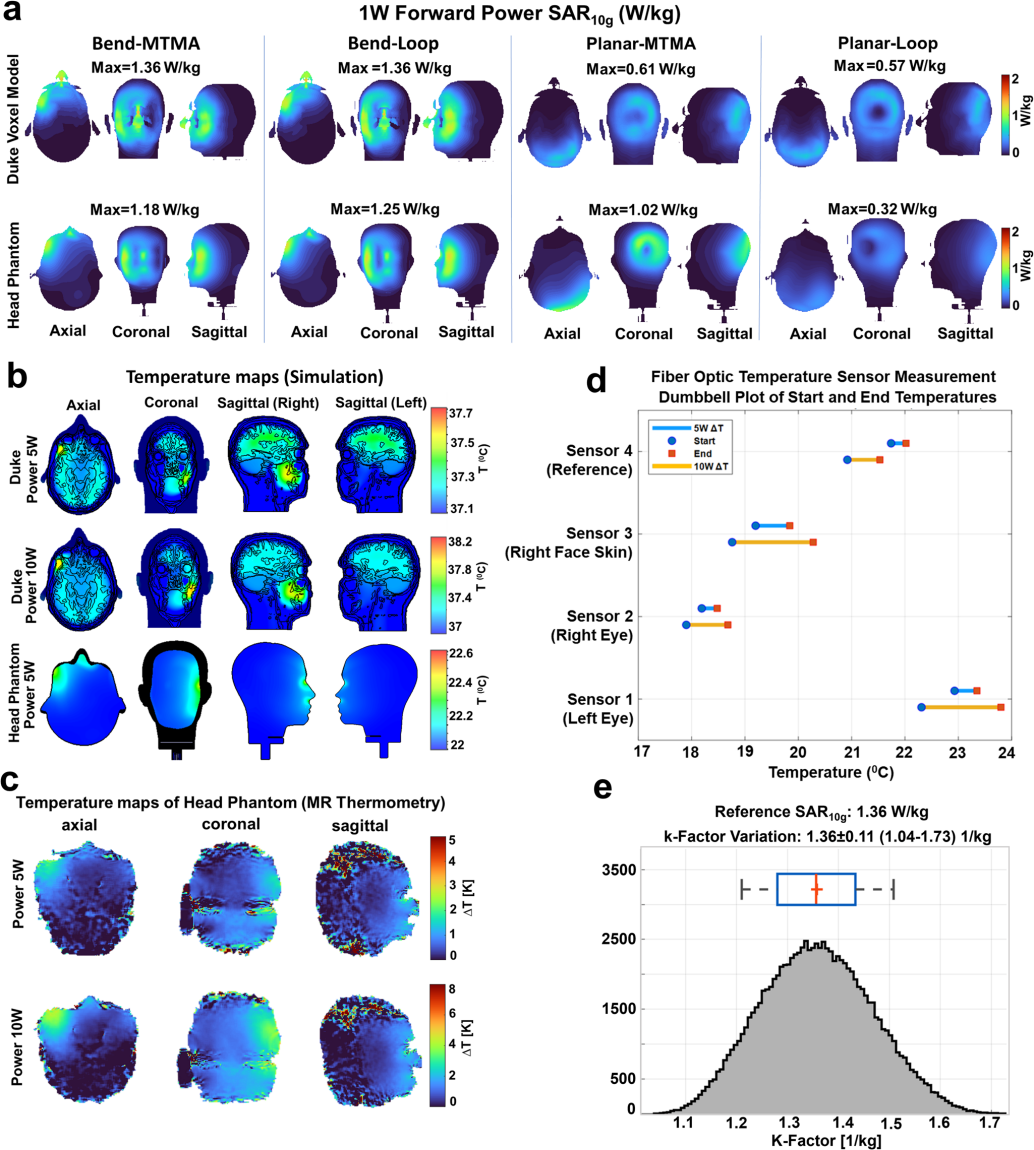
Assessment of Specific Absorption Rate (SAR) averaged over 10g tissue for thermal safety evaluation of the Planar and Bend configurations. (a) Simulated SAR_10g_ maps for 1W forward power using the Bend‐MTMA, Bend‐Loop, Planar‐MTMA, and Planar‐Loop configurations obtained for the human voxel model Duke and an anatomically shaped head phantom. (b) Temperature rise estimated from transient thermal simulations using the Duke model and the head phantom at 5 W and 10 W input powers provided to the Bend‐MTMA configuration. The temperature maps show a minor temperature increase (ΔT≈0.5–0.8 °C versus baseline) in the right facial muscle. (c) Temperature maps validated with PRFS‐based MR thermometry after 30 minutes of RF exposure at 5 W and 10 W using the Bend‐MTMA matched the unilateral heating patterns observed in the thermal simulations. (d) Temperature measurements using fiber‐optic sensors confirmed the results derived from the temperature simulation, with minor localized heating in the right facial muscle (10 W) and overall ΔT <0.5 °C at 5 W. (e) K‐factor histogram used to define the 7.0 T MRI scanner‐specific RF power control limits. A 5 W input was selected for in vivo use to ensure safety, as it results in <0.5 °C temperature rise across all monitored regions.

To evaluate MRI safety, we simulated 10g‐averaged specific absorption rate (SAR_10g_) for all four antenna configurations. RF power deposition was assessed using SAR_10g_, calculated with 1 W of normalized forward input power for all four antenna configurations, based on simulated B₁^+^ distributions in the Duke and head phantom. SAR_10g_ limits following the IEC 60601‐2‐33 guidelines for first‐level mode operation for local SAR (<10 W/kg) and head‐averaged SAR (<3.2 W/kg) [93, 106]. Figure 7a shows, the simulated SAR_10g_ distributions for Planar‐MTMA, Planar‐Loop, Bend‐MTMA, and Bend‐Loop remained well below the IEC 60601‐2‐33 local SAR limit of 10 W/kg for both the Duke and the head phantom setups. The Bend configurations showed the highest localized SAR_10g_ of ≈1.36 W/kg for the human voxel model Duke. Although peak SAR_10g_ (≈1.36 W/kg) were similar, for the Bend‐MTMA and the Bend‐Loop, their spatial distributions differed. The Bend‐Loop configuration produced a unilateral facial hotspot, whereas the Bend‐MTMA configuration showed a more symmetric SAR pattern (Figure 7a). This finding is likely due to the Bend‐Loop's asymmetric B₁^+^ distribution, which concentrates E‐fields locally, while the Bend‐MTMA promotes more uniform field distribution. Despite the MTMA's electric dipole resonance, SAR near the eye remained below 1 W/kg.

#### 2.5.2 Temperature Simulations

Temperature simulations were conducted using CST's thermal solvers to complement SAR_10_g analysis, estimating local heating under RF exposure, incorporating tissue‐specific thermal and dielectric properties [92]. Resulting temperature maps predicted thermal behavior and informed safety thresholds for subsequent in vivo imaging. Temperature simulations focused on the Bend‐MTMA, designed for ocular imaging. The Bend‐MTMA showed the highest simulated SAR_10g_ among all configurations and was therefore selected for bio‐thermal simulations, which were also validated experimentally. Because the eye is sensitive to RF‐induced heating due to its high conductivity, a thorough thermal evaluation is a mandatory prerequisite and safety measure before in vivo application. Temperature simulations were performed on both Duke and a head phantom. For Duke, the baseline steady‐state temperature was first simulated using the thermal steady‐state solver at an ambient temperature of 22°C. Subsequently, a transient temperature rise was simulated from SAR_10g_‐based volumetric power loss (W/m^3^) using the thermal transient solver with isothermal boundary conditions over a 30‐minute RF exposure at input powers of 5 W and 10 W, with tissue‐specific thermal properties from the IT'IS database [92]. The same simulation was applied to the head phantom, and the resulting temperature maps were experimentally validated. Transient thermal simulations for Bend‐MTMA at 10 W input power for 30 minutes revealed maximum local temperature increases of up to ≈0.7°C near the right‐side facial region. The absolute temperatures remained within the IEC threshold of 38°C (Figure 7b).

#### 2.5.3 Thermal Safety Validation: MR Thermometry and Fiber‐Optic Temperature Measurements

Simulated temperature rise associated with SAR_10g_ distributions of Bend‐MTMA were validated using MR thermometry (MRTh) and fiber optic temperature sensors (Omniflex, Neoptix, Québec, Canada) on the head phantom, following the IEC 60601‐2‐33 guideline for local SAR (<10 W/kg), head‐averaged SAR (<3.2 W/kg), and local tissue temperature (<38°C) with increases in core body temperature being limited to ≤1°C [106]. RF heating experiments were conducted at ambient room temperature (≈297 K) with continuous RF input power of 10 W (reference transmitter voltage: 205 V) for 30 minutes. A secondary test at 5 W (reference transmitter voltage: 72 V) was also conducted for an additional safety margin. Power levels were selected based on the scanner's SAR conversion factor (K‐factor), where K = 1 (K = 2 is 5W) corresponds to the system‐imposed limit of 10 W/kg over 6 minutes [90]. Temperature mapping with MRTh was performed using the proton resonance frequency shift (PRFS) method with a dual gradient‐echo sequence [14]. An oil sample was included in the field of view to correct for B_0_ drift. PRFS‐derived temperature maps were compared with readings from four fiber optic sensors placed on the phantom: the left and right eyes, the facial muscle (above the right eye), and a reference sensor positioned at the center of the patient table of the MRI scanner. The fiber optic sensors recorded continuous temperature during RF heating, and the start/end values at each location were documented. The temperature difference (ΔT  =  end – start) was used to verify compliance with the IEC guidelines. Experimental temperature maps derived from MR thermometry validated the thermal simulations, confirming the spatial heating patterns near the right‐sided facial region with the highest temperature increase relative to other regions (Figure 7c). Fiber optic probes validated this finding, reporting a temperature increase of ≈1.5°C at the corresponding location after 30 minutes at 10 W, whereas it remained <0.5°C at 5 W (Figure 7d). Since the 5 W power level consistently produced temperature rises well below IEC safety thresholds, a conservative K‐factor of 2 (5 W input) was selected as a safe operational limit for all in vivo MRI examinations to ensure thermal safety (Figure 7e) [11, 12].

### 2.6 In Vivo Human MRI Validation and Clinical Application

#### 2.6.1 Ethics Statement

For the in vivo feasibility study, all participants were included after approval by the local ethics committee (EA4/084/18, Charité Universitätsmedizin Berlin, Germany) and the Ethics Committee of the University of Rostock (A 2021‐0154) for DFG project number 517901233. Written informed consent was obtained from all participants prior to study participation, in compliance with institutional review board guidelines.

#### 2.6.2 In Vivo MRI Volunteer Study Preparation and Setup

Following assessments of human voxel models and head phantoms, the study proceeded to human in vivo MRI. In vivo MRI of the eye and orbit was performed using the Bend‐MTMA in five healthy adults (1 female, 4 males, aged 24–64 years, mean BMI: 23.8 ± 2.1 kg/m^2^) and in a volunteer with retinal hemangioma (aged 26). Three healthy volunteers (2 males, 1 female) were scanned with both the Bend‐Loop and Bend‐MTMA to enable direct comparison of transmit and receive sensitivity. Stability of the Bend‐MTMA was further assessed through a repeatability study in one volunteer across four MRI sessions on separate days with identical setup and scan parameters. During the MRI, participants were instructed to keep their eyes closed and avoid eye movement. Standard 7.0 T safety instructions were provided before scanning, and a post‐scan interview was documented in accordance with safety protocol. The Bend‐MTMA coil was positioned over the anterior head, centered on the eyes, with ≈1.5 cm spacing from the forehead, ensuring stable, contact‐free placement without facial compression, similar to the setup used in the head‐phantom simulation and experiment. The imaging protocol included transmit B₁^+^ measurement (pre‐saturation‐based mapping) and anatomical scans with standard sequences to assess receive performance. Multi‐sequence receive signal performance was evaluated using T_1_‐weighted gradient‐echo (GRE) and T_2_‐weighted turbo spin‐echo (TSE) imaging, each acquired under steady‐state conditions with sequence‐specific but identical parameters for both Bend‐MTMA and Bend‐Loop. With consistent transmit excitation and tissue properties, signal intensity was used as a metric for relative receive sensitivity. ROIs covering the eye globe were used to extract mean signal intensity values. Transmit B₁^+^ and receive sensitivity were quantified in vivo in axial slices using mean ± SD values from manually defined ROIs shown in bar plots, with corresponding 1D profiles extracted along horizontal and vertical lines. In accordance with ophthalmology conventions, the right and left eyes are referred to as OD (oculus dexter) and OS (oculus sinister), respectively.

Planar configurations were used in two male volunteers for occipital lobe brain imaging, with B₁^+^ mapping (pre‐saturation‐based mapping) and anatomical scans acquired using MPR (Multiplanar Reconstruction) and MP2RAGE (Magnetization Prepared Rapid Gradient Echo) sequences. Planar configurations were placed at the posterior head over the occipital lobe, with ≈1.5 cm spacing from the skull, following the same setup used in the head‐phantom simulations and experiments. B₁^+^ maps (sagittal slice) and signal intensity were compared between Planar‐MTMA and Planar‐Loop.

#### 2.6.3 In Vivo Ocular MRI Results

##### 2.6.3.1 Transmit Performance: B₁^+^ Mapping of the Eye and Orbit

**FIGURE 8:**
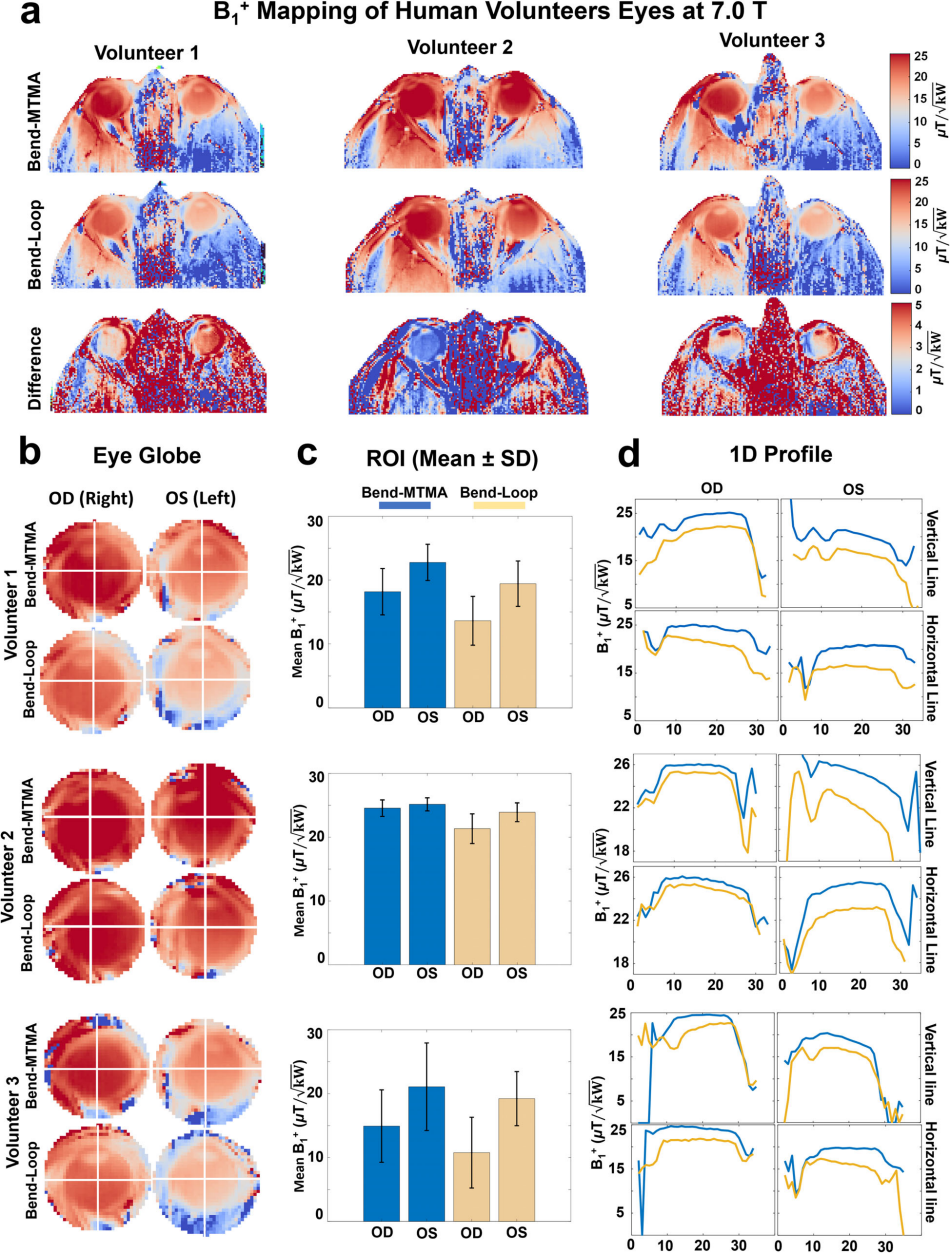
In vivo transmit field B₁^+^ mapping comparing the Bend‐MTMA and Bend‐Loop configurations for three healthy volunteers (2 males, 1 female). (a) B₁^+^ maps (axial slice) show enhanced transmit field in the ocular region for the Bend‐MTMA, with the corresponding difference maps highlighting MTM‐driven B₁^+^ enhancement. (b) Zoomed views of the eye for each volunteer were used as circular ROIs for the quantitative assessment of B₁^+^. Zoomed view used the same scale as the full view above. Vertical/horizontal lines through the eyes were used for 1D profile analysis of B₁^+^. (c) Bar plots showing mean ± SD B₁^+^ in (µT/√kW) within the eye ROIs for each volunteer, demonstrating consistent transmit field enhancement with the Bend‐MTMA compared to the Bend‐Loop. (d) 1D B₁^+^ profiles across both eyes reveal enhanced B₁^+^ depth penetration with the Bend‐MTMA.

To evaluate the transmit performance of the bend antennas, B₁^+^ measurement was performed in the three healthy volunteers (2 males, 1 female), and Figure 8a shows axial B₁^+^ maps of both eyes for three healthy volunteers using the Bend‐MTMA and the Bend‐Loop. Across all volunteers, the Bend‐MTMA produced stronger and more uniform B₁^+^ in the ocular region than the Bend‐Loop, as demonstrated by the difference maps. This enhancement was particularly evident in the OS and aligns with the simulation and experimental results in the head phantom. A closer examination of the eye ROIs (Figure 8b) and their corresponding mean B₁^+^ bar plots (Figure 8c) revealed subject‐dependent gains for the Bend‐MTMA. Volunteer 1 showed B_1_^+^ increases of 33.5% (OD) and 17.2% (OS), Volunteer 2 showed the highest B₁^+^ and most uniform distribution (15% OD, 10% OS), Volunteer 3 showed 40.2% (OD) and 11% (OS) improvement. The Bend‐Loop consistently yielded lower B₁^+^, particularly in OS, consistent with head‐phantom and simulation results. 1D B₁^+^ profiles across both eyes are shown in Figure 8d for all volunteers, illustrating higher B₁^+^ and improved uniformity across OS and OD with the Bend‐MTMA. Although minor asymmetries were obtained between eyes, likely due to anatomical variation, the Bend‐MTMA still consistently enhanced B₁^+^ and coverage across all volunteers.

##### 2.6.3.2 Receive Performance: T_2_‐Weighted TSE Imaging of the Eye and Orbit

**FIGURE 9:**
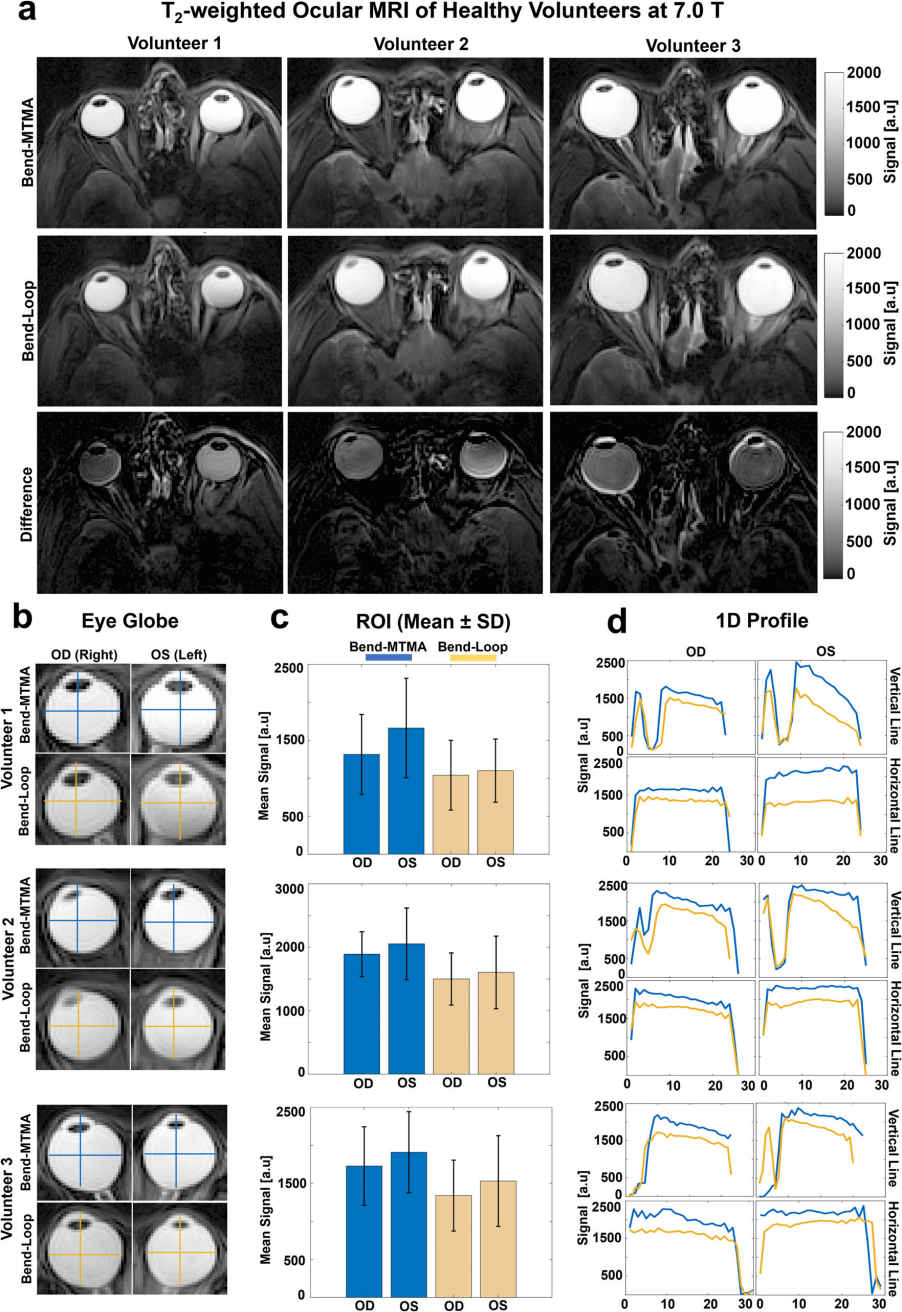
In vivo T_2_‐weighted Turbo Spin Echo (TSE) MRI in the same three healthy volunteers using the Bend‐MTMA and Bend‐Loop. (a) Axial slice images reveal increased intraocular signal for the Bend‐MTMA configuration, while the corresponding difference maps of (MTMA‐Loop) highlight the MTM‐facilitated signal enhancement. (b) Zoomed views of the eye used for eye‐globe ROI analysis. Zoomed view used the same scale as the full view above. Vertical and horizontal lines were used to extract the 1D signal profile. (c) Bar plots showing ocular ROIs’ Mean ± SD signal intensity, demonstrating consistent signal enhancement with the Bend‐MTMA over the Bend‐Loop. (d) 1D signal intensity profiles across both eyes reveal enhanced depth penetration of the signal with the Bend‐MTMA.

To assess the receive performance of the bend antennas, T_2_‐weighted imaging was performed using a turbo spin‐echo (TSE) sequence in the same volunteers who underwent B₁^+^ mapping. Figure 9a shows the receive performance (axial slice) of the Bend‐MTMA and the Bend‐Loop in three volunteers, and the corresponding difference maps highlight the enhanced intraocular signal achieved with the Bend‐MTMA compared to the Bend‐Loop, consistently observed across all volunteers. A closer examination of the eye ROIs (Figure 9b) and their corresponding mean signal‐intensity bar plots (Figure 9c) showed OS signal enhancements of 51%, 28%, and 25% for Volunteers 1, 2, and 3, respectively, with corresponding gains of 27%, 26%, and 29% observed for OD. Although standard deviations were substantial for both configurations due to contrast transitions between the vitreous humor and the lens, MTMA showed consistent relative improvement. 1D signal profiles (Figure 9d) show improved symmetry and deeper signal coverage achieved with the Bend‐MTMA configuration, particularly in OS. These findings align with results obtained for the head phantom and highlight the role of wMTM's near‐field coupling in achieving more uniform signal reception. Overall, the Bend‐MTMA configuration enhanced in vivo T_2_‐weighted image quality, providing more uniform and higher‐intensity signal coverage of the ocular region at 7.0 T than Bend‐Loop.

##### 2.6.3.3 Receive Performance: T_1_‐Weighted GRE Imaging of the Eye and Orbit

**FIGURE 10:**
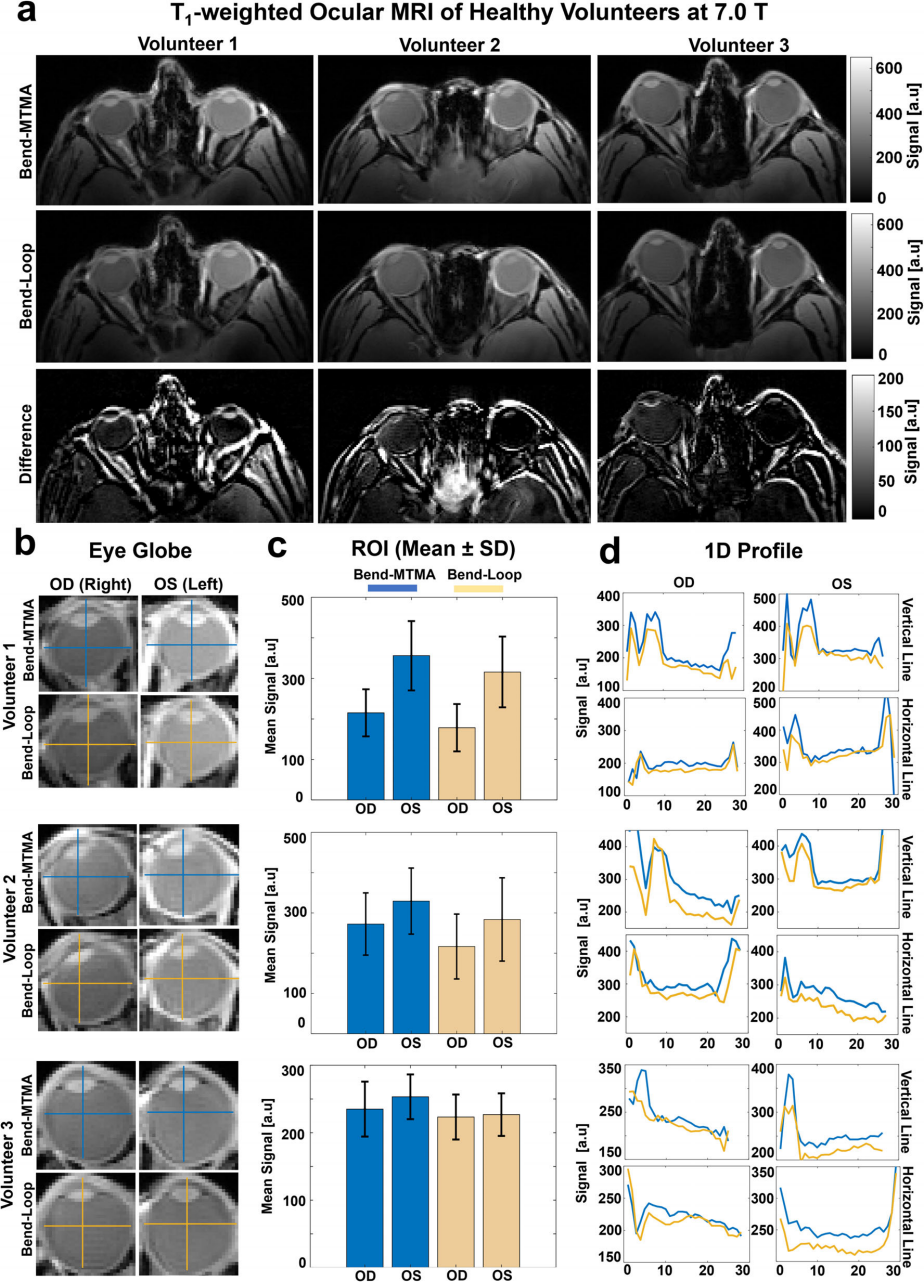
In vivo T_1_‐weighted Gradient Echo Fast Low Angle Shot (GRE FLASH) imaging in three healthy volunteers, comparing the Bend‐MTMA and Bend‐Loop. (a) Axial slice images show increased intraocular signal for the Bend‐MTMA with the corresponding difference maps of (MTMA‐Loop). (b) Zoomed views of the eye showing the eye‐globe ROIs used for signal intensity assessment. Zoomed view used the same scale as the full view above. The vertical and horizontal lines across the eye globe were used to examine 1D signal profiles. (c) Bar plots showing mean ± SD signal intensity within the eye‐globe ROIs demonstrate a consistent, modest signal enhancement with the Bend‐MTMA relative to the Bend‐Loop. The GRE‐FLASH sequence used a low excitation flip angle (α = 6°), a long repetition time (TR), and a short echo time (TE), yielding T_1_‐weighted contrast, thus, the signal is more reflective of receive sensitivity than transmit efficiency. (d) 1D signal intensity profiles across both eyes reveal improved signal depth penetration with the Bend‐MTMA.

Subsequently, the evaluation transitioned from T_2_‐weighted to T_1_‐weighted imaging using the same three volunteers, which provides another clinically relevant MRI contrast mechanism. For T_1_‐weighted gradient‐echo (GRE) imaging, the Bend‐MTMA yielded a higher intraocular signal than the Bend‐Loop for all volunteers (Figure 10a). The eye ROIs (Figure 10b) and their corresponding mean signal‐intensity bar plots (Figure 10c) showed OS signal enhancements of 21%, 16%, and 11% for Volunteers 1, 2, and 3, respectively, with corresponding gains of 13%, 26%, and 7% observed for OD. These findings further support the performance benefits of metamaterial‐integrated RF antenna for ocular imaging at 7.0 T. Differences in receive performance between TSE and GRE sequences highlight the underlying physics of the MTMA's transmit–receive behavior across clinically relevant imaging protocols. High flip angle excitation used in TSE showed greater signal enhancement, reflecting its reliance on transmit fidelity, where the MTMA's enhanced B₁^+^ field provides a clear advantage. In contrast, low flip angle excitation used for GRE demonstrated consistent but less pronounced gains, reflecting its lower dependence on B₁^+^, with improvements still attributed to enhanced receive sensitivity supported by the wMTM.

##### 2.6.3.4 Repeatability Study with Bend‐MTMA

**FIGURE 11:**
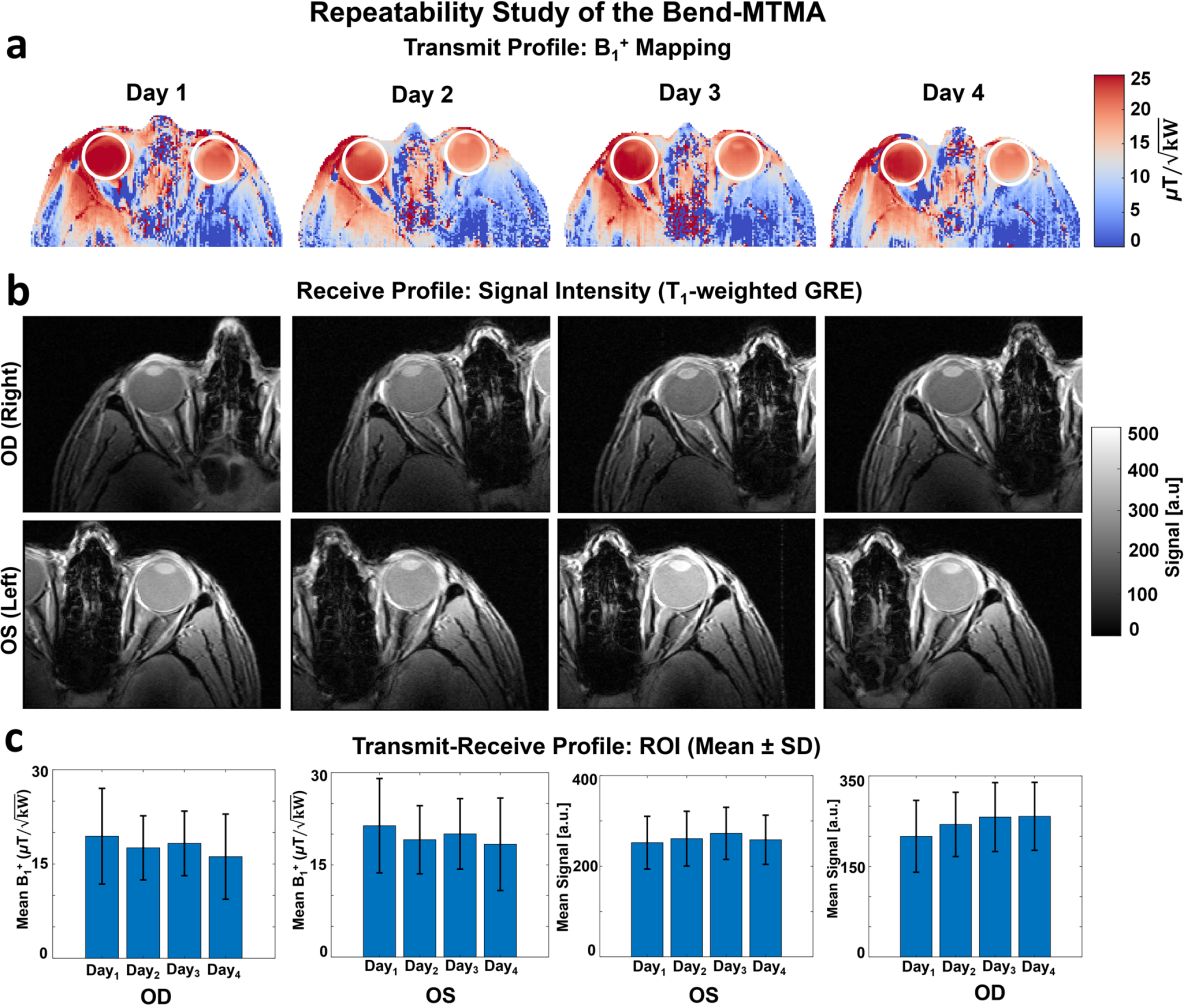
Assessment of intra‐individual repeatability for the Bend‐MTMA configuration across four MRI sessions in a single healthy volunteer. Identical setup and scan parameters were used on separate days to demonstrate consistency, reliability, and validity of the results. (a) In vivo B₁^+^ maps obtained for each of the four sessions, with scan days annotated on top. Mean ± SD B₁^+^ were extracted from manually defined ROIs covering both eye globes. (b) In vivo T_1_‐weighted (GRE FLASH) axial slice images covering both eyes for each session. Mean ± SD signal intensity was calculated across the entire image slice (without ROI masking) to assess global receive sensitivity and image quality across sessions. (c) Bar plots show mean ± SD B₁^+^ (µT/√kW), illustrating minimal variation in transmit fields across independent sessions. Whole‐slice signal intensity analysis revealed a consistent mean ± SD across all four MRI sessions.

Assessing repeatability is an essential prerequisite for the broader clinical application of our metamaterial‐integrated RF antenna. Figure 11 shows repeatability results obtained for the Bend‐MTMA across four independent sessions in Volunteer 1. B₁^+^ mapping and GRE signal intensity showed stable Mean ± SD values in both eyes, with minimal variation, indicating consistent transmit‐receive performance and supporting reproducible behavior of Bend‐MTMA under consistent setup and site conditions (Figure 11b,c).

##### 2.6.3.5 High Spatial Resolution Ocular Imaging in Healthy Volunteers

**FIGURE 12:**
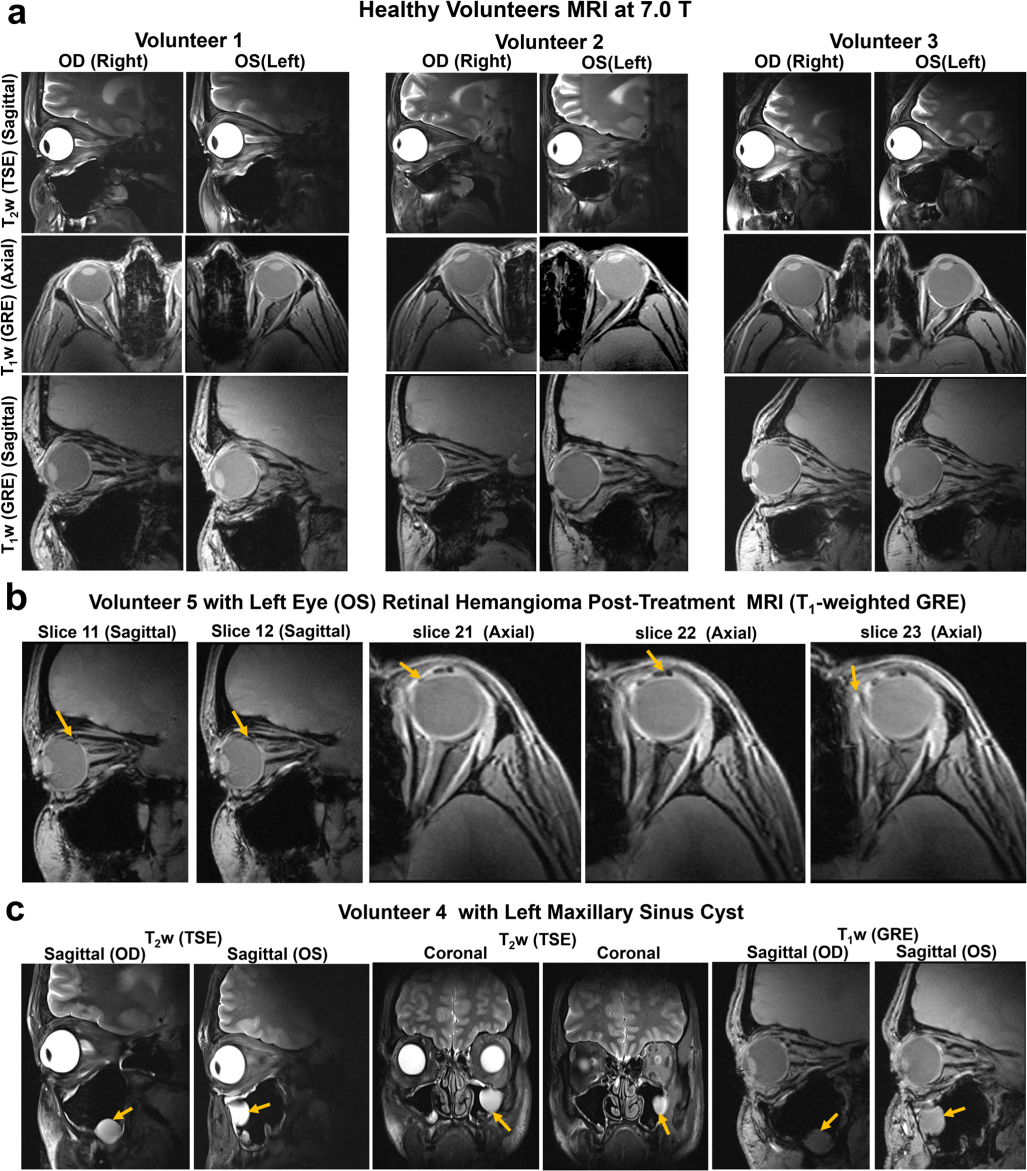
(a) High‐resolution anatomical 7.0 T MRI of three healthy volunteers (2 males, 1 female) using T_2_‐weighted Turbo Spin Echo (TSE, sagittal) and T_1_‐weighted Gradient Echo (GRE, axial/sagittal), acquired with the Bend‐MTMA configuration, enabling detailed visualization of the eye and orbital anatomy. (b) Clinical evaluation in a volunteer with prior retinal hemangioma pathology in the left eye, examined with MRI at 7.0 T using the Bend‐MTMA configuration 16 days post‐treatment. Sagittal and axial views of the eye and orbit reveal post‐treatment structural changes in the globe and anterior orbit, consistent with scarring and a periorbital vascular malformation (yellow arrow). (c) An incidental finding of a left‐sided maxillary sinus cyst (indicated by a yellow arrow) was identified in volunteer 4, appearing hyperintense on T_2_‐weighted MRI and visible on T_1_‐weighted MRI. The coronal slice demonstrates detailed visualization of the anterior cranial structures, including the extraocular muscles, optic nerve, and the cyst adjacent to the left orbit.

High‐resolution anatomical imaging in three healthy volunteers using sagittal TSE (T_2_‐weighted) and axial/sagittal GRE (T_1_‐weighted) MRI with the Bend‐MTMA configuration enabled detailed visualization of orbital anatomy (Figure 12a). GRE imaging provided excellent structural detail, delineating the optic nerves, extraocular muscles, orbital contours, bony margins, and sinus anatomy. TSE imaging complemented this by enhancing fluid‐rich intraocular structures, including the vitreous body, the optic sheath, and the mucous membranes. Both sagittal GRE and TSE views showed strong depiction of the optic nerve pathways, orbital roof, and globe boundaries, with clear visibility of the frontal sinus and anterior cranial structures. This multimodal contrast enabled comprehensive coverage of the orbit and adjacent regions.

##### 2.6.3.6 Clinical Feasibility Study: Incidental and Pathological Findings

Clinical performance of the Bend‐MTMA was evaluated in a volunteer with a prior retinal hemangioma in OS due to genetically confirmed Von Hippel–Lindau disease [107]. The patient underwent repeated transconjunctival cryocoagulation and focal laser photocoagulation as treatment for the retinal hemangioma. MRI at 7.0 T was performed 16 days after the latest treatment using the Bend‐MTMA configuration. The post‐treatment MRI scan revealed two anatomically distinct clusters of structural change (Figure 12b). On sagittal slices 11,12, a T_1_‐hypointense area was observed in the posterior aspect of the left globe, corresponding to the site of the pre‐treated retinal hemangioma. These findings are consistent with post‐interventional scarring with no evidence of lesion progression. Additionally, axial slices 21–23 revealed three T_1_‐hypointense foci anterior to the globe midline, near the lens. These correspond to a previously untreated periorbital vascular malformation located nasally and superiorly to the lens [108]. Focal scarring visible in slice 22–23 suggests vascular remodeling or a secondary therapeutic effect. An incidental left‐sided maxillary sinus cyst was detected in Volunteer 4, appearing hyperintense on T_2_‐weighted TSE imaging and visible on T_1_‐weighted GRE imaging (Figure 12c) [109]. This unexpected finding highlights the added diagnostic value of Bend‐MTMA beyond the orbit, with reliable coverage extending into the paranasal sinuses and inferior frontal lobe. These results demonstrate that the Bend‐MTMA at 7.0 T enables high‐resolution detection of normal ocular and orbital anatomy as well as space‐occupying lesions, post‐interventional changes, and vascular lesions, supporting its translational value in clinical ocular imaging.

#### 2.6.4 In Vivo Occipital Brain Imaging in Healthy Volunteers

**FIGURE 13:**
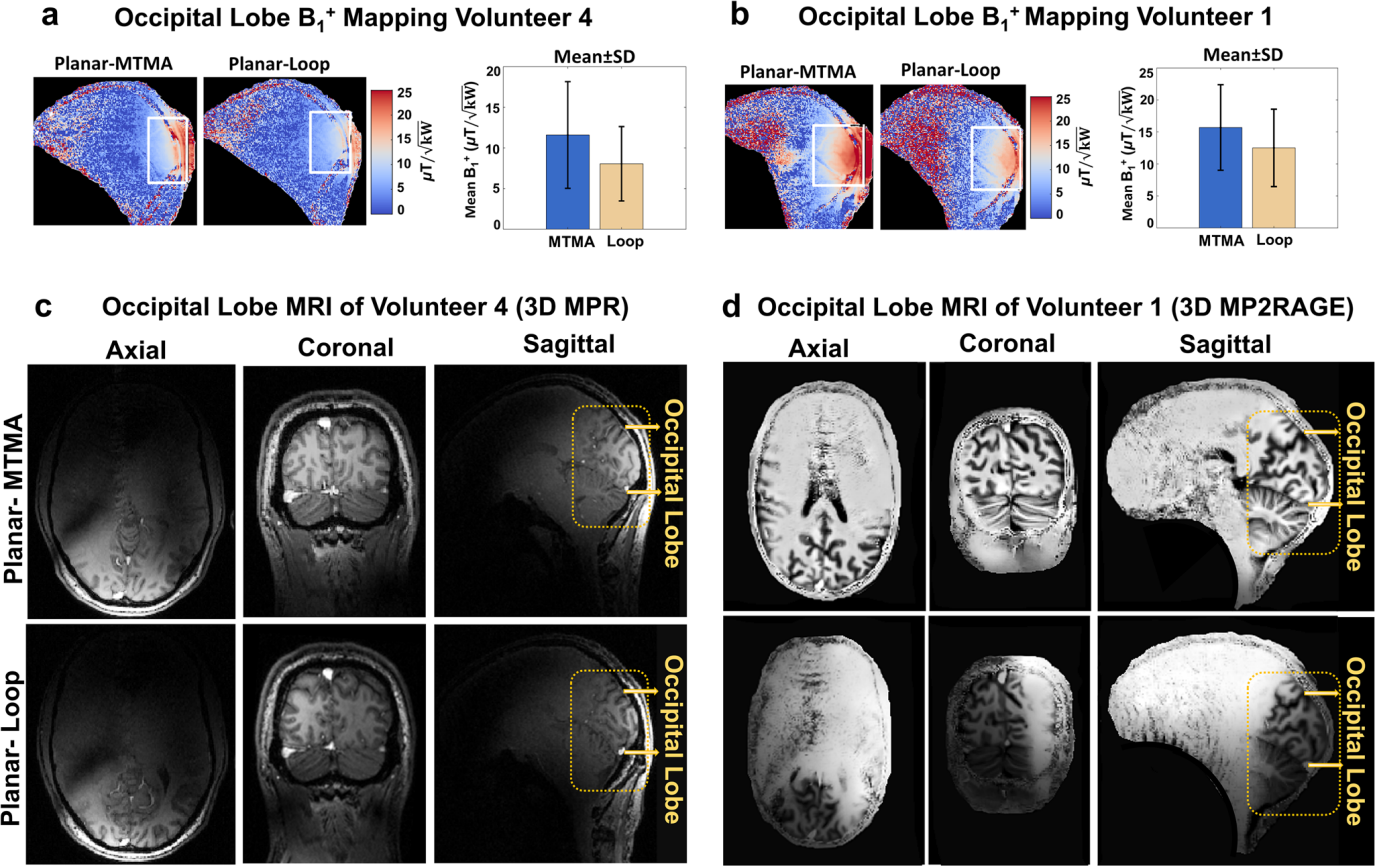
Comparison of the transmit field B₁^+^ mapping and structural MRI of the occipital lobe of the brain using the Planar‐MTMA and Planar‐Loop configuration in two healthy male volunteers. (a, b) Transmit field B₁^+^ maps (sagittal slice) obtained from volunteers 4 and 1 covering the occipital lobe of the brain using the Planar‐MTMA and the Planar‐Loop. The bar plot shows that the Planar‐MTMA yielded a stronger B₁^+^ than the Planar‐Loop for the ROI delineated by the white rectangle. (c) Sagittal, axial, and coronal T_1_‐weighted images of the occipital lobe derived from 3D MPR MRI of volunteer 4 and (d) 3D MP2RAGE MRI of volunteer 1 using the Planar‐MTMA and Planar‐Loop. Yellow dashed boxes denote the occipital lobe region of the brain. The Planar‐MTMA improved signal coverage at greater depths, enhancing the visibility of posterior brain structures, including the cerebellum. In contrast, images acquired with the Planar‐Loop exhibited reduced signal intensity and more limited coverage in deeper brain regions.

To assess the transmit performance and signal coverage of the planar antennas, we focused on the occipital lobe, a clinically relevant target for neuro‐ophthalmic examinations [110]. Figure 13 illustrates the results of occipital lobe imaging in two healthy volunteers using planar antennas. B₁^+^ maps acquired with the Planar‐MTMA showed significantly higher transmit efficiency than the Planar‐Loop, with gains of 44% and 30% in Volunteers 4 and 1, respectively (Figure 13a,b). Structural MRI using T_1_‐weighted MPR and MP2RAGE sequences with the Planar‐MTMA demonstrated greater signal coverage than the Planar‐Loop, resulting in enhanced anatomical depiction of posterior brain regions, including the cerebellum. In contrast, the Planar‐Loop showed reduced signal and limited coverage (Figure 13c,d).

## 3 Discussion

This work demonstrates feasibility and presents a detailed proof‐of‐concept pipeline for a new class of epsilon‐negative UC metamaterial‐integrated RF antenna for MRI, moving beyond conventional loop‐based designs to improve B₁^+^ and receive efficiency. Central to this approach is our custom‐designed subwavelength DS‐SRR UC, which exhibits tailored epsilon‐negative behavior near resonance (≈297.2 MHz) without negative µ_eff_ and therefore functions as a non‐magnetic dielectric resonator. Although MRI excitation is driven by magnetic fields, our ENG UC is designed to shape the near field via an electric dipole response, a mechanism that aligns with prior studies using high‐permittivity dielectric or electrically resonant metamaterials [18]. In contrast to conventional resonators in MRI, which enhance transmit‐receive fields locally without altering the medium's electromagnetic properties, the DS‐SRR UC operates with engineered negative permittivity, thereby modifying the effective medium, controlling resonance behavior, and shaping the resulting E–H field topology. This UC geometry provides strong capacitive and inductive loading, as well as a localized H‐field. Further, the parametric analysis shows that simple geometric adjustments of the UC geometry can shift the resonance to other MRI magnetic field strengths (e.g., 5.0 T, 9.4 T, 10.5 T, 11.7 T, 14.0 T) or alternatively configure the UC as an MNG variant, supporting application‐specific E–H field control. The UC design also provides a controllable balance between E‐H fields, which allows the E‐field to remain well controlled for SAR safety while still producing a strong and useful H‐field for MRI performance. Because the UC is subwavelength (λ/38), it supports a dense periodic array, which is essential for creating the collective electromagnetic mode required for near field control in MRI. (S4) further illustrates how the UC dimensions govern the local E–H field intensity, spatial pattern, and the near field reach of the MTM surface. Smaller ENG UCs generate stronger, more confined E–H fields that sustain higher H‐field strength both at the array level and at greater distances from the MTM surface (array), thereby enhancing the effective near‐field reach when incorporated into the RF antenna system. Furthermore, array‐level simulations indicate that assembling the UCs into an MTM surface produces a collective electromagnetic response with a broader, centrally intensified H‐field that couples efficiently to the integrated loop coil through near‐field interaction, providing the physical basis for the stronger signal and greater coverage in depth observed with the MTMA design. These degrees of freedom extend beyond conventional resonator approaches and enable controlled E‐field behavior for SAR safety, together with a strong, spatially coherent H‐field for enhanced transmit–receive performance. Collectively, these properties establish the proposed UC as a scalable, circuit‐free platform that is parametrically tunable, geometrically reconfigurable, and electrically adjustable, supporting integration to various RF coil geometries and Larmor frequencies across higher and lower magnetic field strengths, including those common in clinical MRI practice [25, 86, 111]. UCs dimensions remain subwavelength at 297.2 MHz (≈λ/38), satisfying the effective medium ratio (EMR) criterion [82, 83]. This enabled the construction of a lightweight, thin 40‐UCs wMTM (5 × 8 array of UC) layer, which was integrated into both planar and anatomically bend 2‐channel RF loop. The 5 × 8 UC layout offered a practical balance, ensuring subwavelength conditions for eigenmode formation, thereby supporting a collective electromagnetic response that produces a broader, centrally intensified H‐field at 297.2 MHz, while also maintaining favorable E‐field performance for SAR safety, anatomical coverage, and integration feasibility.

The relatively large loop size (≈240 mm × 150 mm) was chosen to accommodate the wMTM layer without altering the intrinsic UC response, while also ensuring adequate anatomical coverage for ocular and occipital imaging. While smaller loops may improve intrinsic B₁^+^ uniformity, reducing the loop size would require proportionally smaller UCs, thereby increasing the complexity of resonance tuning, as it necessitates substantial redesign of the capacitive and inductive loading and the fabrication process at ≈300 MHz [88]. Importantly, the larger loop increases baseline asymmetry, allowing a clear demonstration of the wMTM's ability to restore transmit field uniformity. The overall MTMA size (≈240 × 150 × 1.52 mm^3^) determines the extent of the imaging region that benefits from the metamaterial‐enabled RF field enhancement. Matching the MTM dimensions to the loop footprint ensures that the field shaping spans the entire antenna rather than a local area. This provides sufficient FOV and anatomic coverage for ocular and anterior cranial imaging while maintaining enhanced transmit–receive performance. Furthermore, in vivo results show that MTMA's overall size enables conformal placement along the facial contours and high‐resolution imaging of the sinuses and anterior cranial regions.

The current 2‐channel setup functions as a multi‐channel system, though it is not yet a high‐density configuration. Despite its simplicity, this study advances prior MTM‐enhanced single‐loop demonstrations by establishing a dual‐channel platform as an important technical foundation for future high‐density, scalable MTM‐integrated antenna array deployment for MRI [76, 77, 78, 79]. Our study further advances beyond prior metamaterial MRI work, which primarily employed bulk dielectric‐based or thin, flexible receive‐only MTM add‐ons [15, 16, 17, 18, 26, 32, 46, 47, 48, 49, 50, 51, 52, 53, 54, 55, 56, 57, 58, 59, 60, 61, 62, 63, 64, 65, 66, 67, 68, 69, 70, 71, 72, 73, 74, 75]. Unlike these previous approaches, we present a structurally MTM‐integrated 2‐channel RF antenna in both planar and bend configurations, in which the ENG DS‐SRR unit‐cell array and the loop element operate as a single, unified resonant system. Distinct from passive resonators or MTM add‐ons that locally boost receive sensitivity, in our MTMA, the wMTM layer couples to the loop's RF field through controlled near‐field evanescent coupling and enhances the transmit–receive field in MRI [77, 102, 103]. The performance of detached (add‐on) metamaterial surfaces used in conjunction with a conventional RF coil often depends on precise and reproducible placement relative to the RF coil, including spacing and alignment. In our MTMA integrated‐antenna design, the MTM layer and loop element share the same PCB substrate, ensuring stable positioning and loading, since loading does not depend on MTM–coil spacing or air gaps. These benefits are directly reflected in the rectangular phantom results, where the integrated MTMA shows consistently higher transmit and receive sensitivity than the reference loop antenna and detached metamaterial “add‐ons” setup. This architecture explains the greater receive enhancement observed with the planar‐MTMA configuration, reflecting its role as a passive electromagnetic lens that reinforces receive sensitivity through near‐field resonance [98, 99, 100, 101, 102, 103, 104]. Detached metamaterial surfaces remain useful when coil‐integrated designs are not feasible. However, an MTM‐integrated antenna provides a unified co‐resonant platform that can be extended beyond our two‐channel prototype and offers a pathway toward next‐generation, high‐density RF antenna architectures that move beyond conventional loop or dipole designs.

We further demonstrate that both the Planar‐ and Bend‐MTMA are fully compatible with parallel MRI, yielding stable GRAPPA and mSENSE reconstructions while maintaining higher receive sensitivity than the corresponding Loop configurations. In clinical settings, PI compatibility is therefore an important consideration for translating MTMA systems. Previous metamaterial approaches in MRI that demonstrated PI capabilities have relied on add‐on metamaterial structures placed near the patient or adjacent to existing multichannel commercial or custom‐made RF coil arrays [21, 26, 27]. In these prior studies, the parallel imaging performance was determined primarily by the underlying multichannel RF coil array, while the add‐on metamaterial provided only local SNR modulation. In this study, the MTMA itself serves as a 2‐channel array that demonstrates parallel imaging performance, rather than using a separate multichannel coil augmented with an add‐on metamaterial, as in previous work. Therefore, the results presented here represent a substantive advancement in metamaterial‐integrated antenna technology for MRI and, to the best of our knowledge, demonstrate the first metamaterial‐integrated RF antenna that maintains stable performance under parallel MRI.

This work demonstrates the first in vivo application of a metamaterial‐integrated antenna (MTMA) for human eye and orbit MRI at 7.0 T, including quantitative B₁^+^ mapping, transmit‐receive performance benchmarking in volunteers against conventional loop‐based RF arrays of similar geometry, and imaging of ocular pathology. The eye was chosen for its clinical relevance, compact anatomy, and RF heating sensitivity, making it an ideal region and clinically relevant use case for evaluating MTMA's imaging and safety performance to meet the growing demand for high‐resolution MRI in ophthalmology. Our results provide comprehensive validation of conventional add‐on and our structurally integrated MTM configurations in numerical simulations and in phantom and in vivo studies, facilitating a swift transition from theoretical development to clinical applicability. System‐level characterization of transmit‐receive performance was complemented by a multi‐tiered safety validation protocol, including human‐voxel‐model‐based SAR simulations, bio‐thermal modeling, MR thermometry, and fiber optic temperature measurements. While ENG structures are known to enhance local E‐fields, none of the MTMA configurations exhibited excessive RF power deposition. Instead, it exhibited more spatially uniform SAR distributions than the conventional loop coil. These findings confirm safety compliance for in vivo MRI at UHF. Clinical eye RF coils often include openings to support eye gaze fixation to reduce bulk eye motion [12]. Although the current design lacks a dedicated cutout, the 1.5 cm foam interface provided sufficient clearance for open‐eye fixation. This setup was compatible with structural imaging and would support the presentation of visual stimuli. Although the wMTM forms a continuous surface, the design remains adaptable for future applications that require an open cutout.

In vivo imaging in volunteers demonstrated the Bend‐MTMA's versatility across clinically relevant imaging protocols. Differences in receive performance between TSE and GRE imaging reflect the sequence‐dependent influence of transmit uniformity and receive sensitivity achieved with the Bend‐MTMA. Elevated standard deviations in ophthalmic ROIs likely reflect physiological heterogeneity, particularly the pronounced intensity contrast between the vitreous humor and the lens. Some residual B₁^+^ inhomogeneity may arise from the large loop geometry, however, the integrated wMTM layer helped mitigate it. To further illustrate clinical relevance, a Von Hippel‐Lindau associated case of retinal hemangioma demonstrated the clinical potential of the Bend‐MTMA [107]. It enabled clear visualization of both a post‐treatment lesion site and an untreated periorbital vascular abnormality [108]. Additionally, in a healthy volunteer, imaging with the Bend‐MTMA revealed an incidental maxillary sinus cyst, highlighting its ability to capture clinically relevant findings beyond the orbit [109]. These results reinforce the Bend‐MTMA's value for high‐resolution imaging in anatomically constrained regions. Although repeatability testing was limited to one participant, the results demonstrated consistent performance stability and reliability of the Bend‐MTMA. Although this study included representative volunteers and a single clinical case, these datasets were sufficient to demonstrate MTMA's capability. Additional patient data will be acquired in future studies to further expand its diagnostic utility. Our MTMA configurations could be extended to simultaneously image the eye and occipital lobe in a single setup, enabling broad visualization of the visual pathways.

Both MTMA configurations demonstrated enhanced depth penetration, attributed to the wMTM layer's front‐end resonant collector behavior. Depth penetration denotes the RF field's ability to reach and image deeper anatomical regions in MRI [68, 105]. Since MRI depth penetration depends on the full RF coil–sample interaction, the antenna‐level contribution reflected by the H‐field decay inside the rectangular phantom shows that the Planar‐MTMA exhibits a slower effective decay and preserves higher |H| at deeper locations than the Planar‐Loop, consistent with the enhanced depth penetration provided by the integrated wMTM layer (see S4) [68, 105]. This enhancement manifests as improved B₁^+^, stronger signal in deeper regions of the phantom, and extended anatomical coverage across the orbit, paranasal sinuses, and anterior brain, as well as in the occipital region. The Bend‐MTMA enabled improved coverage of deeper ocular and cranial structures despite curvature. This MTMA framework can be extended beyond ophthalmic applications, and the depth‐penetration enhancement demonstrated by the Planar‐MTMA configuration offers potential for cardiac or abdominal MRI that require deep‐tissue imaging [112].

Our structurally metamaterial‐integrated RF antenna approach provides a compact, circuitry‐free alternative for targeted imaging, particularly when pTx systems or high‐density arrays are inaccessible or constrained by regulation. Smart, actively tunable metamaterials incorporating PIN diodes or varactors could enable real‐time field shaping, steering, or detuning, offering a low‐complexity alternative to high‐density arrays and supporting B₁^+^ feedback–driven adaptation to patient‐specific conditions [74, 75]. Developing UC designs with µ‐negative or double‐negative properties could enable magnetic field control, complementing electric dipole MTMs and supporting more adaptive RF coil systems [99]. Future UC architectures inspired by antenna and radar technologies could enable high resolution, directional control by miniaturizing and configuring elements as independent passive receive units for compact, high‐density MTM arrays [113]. Miniaturization strategies for UC for high‐density integration remain a promising direction for ongoing development (S4). The parallel MRI findings further indicate that metamaterial‐integrated antenna arrays could be extended beyond the present 2‐channel configuration. With additional channels, such arrays may support higher acceleration factors and have the potential to offer improved parallel MRI performance compared with conventional loop‐based designs of similar channel count, thereby enabling faster acquisitions in clinical settings. This result opens a new research direction in which UC geometry, resonance behavior, and inter‐element coupling are co‐designed to engineer per‐channel receive‐sensitivity patterns for advanced PI encoding. Such a framework suggests that future metamaterial‐integrated RF coil arrays could be systematically optimized for targeted acceleration performance, in which channel behavior is governed by metamaterial‐driven field shaping rather than by geometric coil placement alone. Together, these insights highlight how next‐generation metamaterial‐integrated antennas may support higher‐acceleration MRI strategies [21]. Furthermore, the MTM framework can be adapted to either suppress local E‐fields for implant safety or amplify them to enhance RF energy delivery in interventional Thermal Magnetic Resonance (ThermalMR) applications, such as hyperthermia and ablation [14, 114]. Broadband MTM antennas may enable simultaneous ^1^H/X‐nuclei imaging for theranostic platforms involving anatomical, physiological, metabolic, and drug‐tracking readouts. MTM‐enhanced X‐nuclei antenna (e.g., ^1^ ^9^F, ^2^ ^3^Na) could also improve transmit‐receive performance, supporting noninvasive monitoring of labeled therapeutics [115, 116]. The MTMA architecture supports adaptation to both human and preclinical platforms, enabling broader clinical and research translation.

## 4 Conclusions

This work introduces a clinically translatable metamaterial‐integrated RF antenna (MTMA) platform for 7.0T MRI, establishing clinical application of electromagnetic metamaterials. The MTMA, which integrates a metamaterial composed of epsilon‐negative unit cells into planar and bendable loop array configurations, demonstrated significantly enhanced transmit–receive performance for MRI of the eye, orbit, and occipital lobe of the brain. Comprehensive quantitative validation and patient imaging support their clinical feasibility for high‐resolution head MRI beyond ophthalmology. The proposed MTMA architecture outperformed conventional RF coil designs and offers a modular, scalable framework adaptable to various MRI magnetic field strengths. This study establishes metamaterials not as passive MRI enhancers, but as foundational components en‐route to the development of next‐generation, metamaterial‐loaded, compact, anatomically tailored RF antennas for precision MRI.

## 5 Methods

### 5.1 Numerical Simulations and Post‐Processing

Numerical electromagnetic (EM) simulations formed the foundation for developing the MTM‐loaded RF antennas. Incorporating realistic human voxel models and phantom setups enabled evaluation of B₁^+^ performance and RF power deposition for safety assessment, thereby guiding key decisions on geometry, layout, and configuration prior to fabrication and validation. The full system, including the UC, MTM, and MTM‐integrated RF antennas, was designed in CST Microwave Studio Suite 2020 (Dassault Systèmes, Germany). All MRI images, B₁^+^ maps, and SAR_10g_ maps were post‐processed in MATLAB R2020a (MathWorks, Natick, USA).

### 5.2 Bench Measurement and 3D‐Printer Hardware

The wMTM and all four antenna configurations were fabricated and experimentally validated through bench measurements. S‐parameters (S_11_, S_2__2_, S_2__1_, S_1__2_) were measured for each antenna configuration, including the wMTM, at 297.2 MHz using a vector network analyzer (VNA) (ZVT 8, Rohde & Schwarz, Germany). A custom 3D‐printed casing for the antenna holders was produced using a Fuse 1+ 30 W printer (Formlabs Inc., Somerville, MA, USA).

### 5.3 MRI Hardware and Protocols

^1^H MRI and ^1^H MRTh were performed on a 7.0 T whole‐body MRI system (MAGNETOM, Siemens Healthineers, Erlangen, Germany, software version VB17) equipped with an 8‐kW single‐channel RF amplifier (Stolberg HF‐Technik AG, Stolberg‐Vicht, Germany) and a gradient system with a maximum slew rate of 200 mT/m/ms and maximum gradient strength of 45 mT/m (Siemens Healthineers). All MRI scan protocols are given in Table S1.

## Author Contributions

**Nandita Saha, M.Sc** (Conceptualization: lead; Data curation: lead; Formal analysis: lead; Investigation: lead; Methodology: lead; Project administration: lead; Software: lead; Supervision: supporting; Validation: lead; Visualization: lead; Writing – original draft: lead; Writing – review & editing: lead) **Bilguun Nurzed** (Methodology: supporting; Validation: supporting) **Mostafa Berangi, PhD** (Methodology: supporting) **Andre Kuehne, PhD** (Methodology: supporting) **Helmar Waiczies, PhD** (Methodology: supporting) **Igor Fabian Tellez Ceja, M.Sc** (Data curation: supporting) **Xiang Hu, M.Sc** (Data curation: supporting) **Thomas Gladytz, PhD** (Data curation: supporting) **Lisa Krenz, Radiographer Technician (Study Nurse)** (Data curation: supporting) **Dave Huebler, Radiographer Technician** (Data curation: supporting) **Beate Endemann, Radiologist** (Data curation: supporting; Investigation: supporting) **Claudia Brockmann, Medical Doctor** (Investigation: supporting) **Ebba Beller, Radiologist** (Data curation: supporting; Funding acquisition: lead; Investigation: supporting) **Oliver Stachs, PhD** (Data curation: supporting; Funding acquisition: lead; Investigation: supporting; Resources: supporting) **Thoralf Niendorf, PhD** (Conceptualization: equal; Funding acquisition: lead; Resources: lead; Supervision: lead; Validation: supporting; Visualization: supporting; Writing – review & editing: supporting).

## Funding

This project was funded by the European Research Council (ERC) under the European Union's Horizon 2020 Research and Innovation Program, grant agreement No. 101248030 (MR‐ACT, TN) and by the Deutsche Forschungsgemeinschaft (DFG, German Research Foundation, DFG‐Project number: 517901233 (TN, OS, EB). N.S. and T.N. received funding from the Helmholtz International Research School iNAMES (Imaging and Data Science from the NAno to the MESo).

## Consent

Written informed consent was obtained from all participants before the in vivo study in compliance with the institutional review board guidelines.

## Institutional Review Board Statement

The in vivo study was approved by the local ethics committee (EA4/084/18, Charité Universitätsmedizin Berlin, Germany) and the Ethics Committee of the University of Rostock (A 2021‐0154).

## Conflicts of Interest

Thoralf Niendorf is founder and CEO of MRI.TOOLS GmbH, Berlin, Germany.

## Supporting information




**Supporting File**: adma72291‐sup‐0001‐SuppMat.docx.

## Data Availability

The data that support the findings of this study are available on request from the corresponding author. The data are not publicly available due to privacy or ethical restrictions.
