# Supplementary material for: Metamaterial Antennas Enhance MRI of the Eye and Occipital Brain
Source: Adv Mater. 2026 Feb 2;38(32):e17760. doi: 10.1002/adma.202517760 (PMC13244817; doi:10.1002/adma.202517760)
Supplement: Supplementary file 1 — Supporting File: adma72291‐sup‐0001‐SuppMat.docx. [file ADMA-38-e17760-s001.docx]

Supporting Information

Metamaterial Antennas Enhance MRI of the Eye and Occipital Brain

Nandita Saha, Bilguun Nurzed, Mostafa Berangi, Andre Kuehne, Helmar Waiczies, Igor Fabian Tellez Ceja, Xiang Hu, Thomas Gladytz, Lisa Krenz, Dave Huebler, Beate Endemann, Claudia Brockmann, Ebba Beller, Oliver Stachs and Thoralf Niendorf*

**S1: Equivalent circuit modeling for the metamaterial unit cell**

***Equivalent Capacitance Model of the Metamaterial Unit Cell***

The total equivalent capacitance was calculated by combining the contributions from Ring 1, Ring 2, and the inter-ring distributed capacitance of DS-SRR unit cell. Each split-gap capacitor C_g_ was modeled as a parallel-plate structure, where g is the split-gap distance, and with the overlapping conductor area.^[1-8]^ The inter-ring capacitance C_s_ was approximated as a distributed sidewall capacitance across all four edges of Ring 2.

As all Gaps are identical: $Cg₁ =Cg₁₁ =Cg₂ =Cg$

**Step 1:** Ring 1’s total capacitance C₁ is from two gaps capacitance i.e. top gap (Cg₁) and side gap (Cg₁₁) which are in series with parallel to inter-ring space capacitance Cs.

$$C₁ = (Cg₁ series Cg₁₁) ║ Cs = (\frac{1}{Cg₁}+\frac{1}{Cg₁₁}) + Cs = (\frac{Cg}{2}) + Cs$$

**Step 2:** Ring 2’s total capacitance $C₂$ is from one gap capacitance i.e. bottom gap ($Cg₂$) in Parallel with Cs

$$C₂ =Cg₂║ Cs = Cg₂ + Cs = Cg + Cs$$

**Step 3:** Total equivalent capacitance

$$C_{eq}= C₁ + C₂ = (\frac{3Cg}{2}) + 2Cs$$

The split-gap capacitance C_g_ and inter-ring capacitance Cs modeled as a parallel-plate capacitor:

$$C_{g}=\varepsilon_{0}\varepsilon_{r}\cdot\frac{A_{g}}{d}$$

$$C_{s}=\varepsilon_{0}\varepsilon_{r}\cdot\frac{A_{s}}{s}$$

where:

- $\varepsilon_{0} = 8.85\times{10}^{-12}F/m$​, vacuum permittivity.
- $\varepsilon_{r} =$ relative permittivity between the plates (substrate Rogers 4360G2 $\varepsilon_{r} = 6.15$)
- $A_{g} = w.t$ = area of the gap (t = copper thickness, w = width)
- $A_{s} = (4*side length \times trace width)$ area in-between Ring 1 and Ring 2 i.e. like two microstrip lines side-by-side capacitance in parallel
- s = spacing between the two traces (inter-ring gap)
- $d=g$ the gap distance between plates

These are simplified parallel-plate approximations, they don’t capture fringe fields or edge effects.

***Equivalent Inductance Model of the Metamaterial Unit Cell***

The total inductance L_eq_ of the unit cell was modeled as the sum of the self-inductances of Ring 1 (L_1_) and Ring 2 (L_2_), along with the contribution from a short interconnecting stub (L_strip_). The inductances of Ring 1, Ring 2, and the connecting strip were modeled in series, as the current flows sequentially through all segments of the unit cell. Each ring's inductance was estimated using a rectangular loop approximation, considering the adjusted physical side lengths to exclude the gap regions. The effective conductor diameter was modeled using d_eff_, a geometric mean that approximates field confinement in flat copper traces, where w is the trace width and t is the copper thickness. This formulation is consistent with planar inductor theory and provides a compact estimate of loop inductance under high-frequency conditions.^[9-15]^ Total equivalent inductance is Ring 1 $(L₁)$ , Ring 2 $(L₂)$ and two short metal stub strip’s inductances $L_{\mathrm{strip}}$ in series.

$$L_{eq}= L₁ + L₂ + L_{strip}$$

Each loop inductance (L) is calculated using:^[9]^

$$L=(\frac{\mu_{0}\mu_{r}}{\pi})\times\left[ -2\left( W+H \right)+2\sqrt{\left( W^{2}+H^{2} \right)}-H\cdot ln\left( \left( H+\sqrt{\left( W^{2}+H^{2} \right)} \right)/W \right)-W\cdot ln\left( \left( W+\sqrt{\left( W^{2}+H^{2} \right)} \right)/H \right)+H\cdot ln\left( 2H/\left( d_{eff} \right) \right)+W\cdot ln\left( 2W/\left( d_{eff} \right) \right) \right]$$

Where,

• $d_{\mathrm{eff}}=\sqrt{\left( w\cdot t \right)}$ is the effective conductor diameter, modeled for flat copper traces

• W and H are the effective lengths of each ring side, adjusted for split gaps using:

• Ring 1: $W = H = 2.5 cm - 2w = 2.3 cm$

• Ring 2: $W = H = 2.26 cm - w = 2.16 cm$

• μ₀ = 4π × 10⁻⁷ H/m, vacuum permeability and $\mu_{r} =1$, relative permeability of air

The two short metal strips each (s × w × t) (0.02 cm × 0.1 cm × 35 μm) were modeled using the flat wire Inductor, yielding ~0.57 nH per strip.^[15]^ Their combined contribution of ~1.13 nH was added in series with the ring inductances to form the total equivalent inductance.

***Resonance Frequency Calculation for Metamaterial Unit Cell***

$$L_{eq}=168.84 nH (L₁\approx87.0 nH; L₂\approx80.7 nH; L_{strip}\approx1.14 nH)$$

$$Cg = 1.91 \times10⁻¹⁵ F; Cs = 861.4 \times10⁻¹⁵ F$$

$$C_{eq}\approx1.73\times{10}^{-12} F$$

Resonance Frequency:

$$f=\frac{1}{2\pi\sqrt{\left( L_{eq}\cdot C_{eq} \right)}} = \boldsymbol{294.85 MHz}$$

The above resonance frequency was calculated using the standard LC resonance formula.^[1-8]^
Using the inductance and capacitance values derived from the above model, the analytically estimated resonance frequency was found to be approximately ~294.85 MHz in the range of the CST simulation frequency ~297.2 MHz (7.0 T Larmor frequency). The equivalent circuit model was developed to estimate initial parameters for designing the unit cell. It provides a simplified understanding of how the ring geometry, gap size, and coupling affect resonance. This analytical framework helped us identify reasonable starting values for the physical parameters, which were later refined using full-wave CST simulations. The model does not explicitly capture fringing fields near the substrate edges, and the inter-rings capacitance does not account for local field variations at the corners or edge split gaps or region with metal strips. Mutual inductance between the rings was avoided for simplicity, as its impact on the resonance frequency has been shown to be minimal in similar geometries, compared to the dominant lumped elements.^[8,13]^ Due to the simplified model, there is a slight offset between the analytically estimated and simulated resonance frequencies. However, these secondary field interactions are inherently captured in full-wave CST simulations. In future work, the analytical model can be expanded to incorporate these effects more comprehensively.


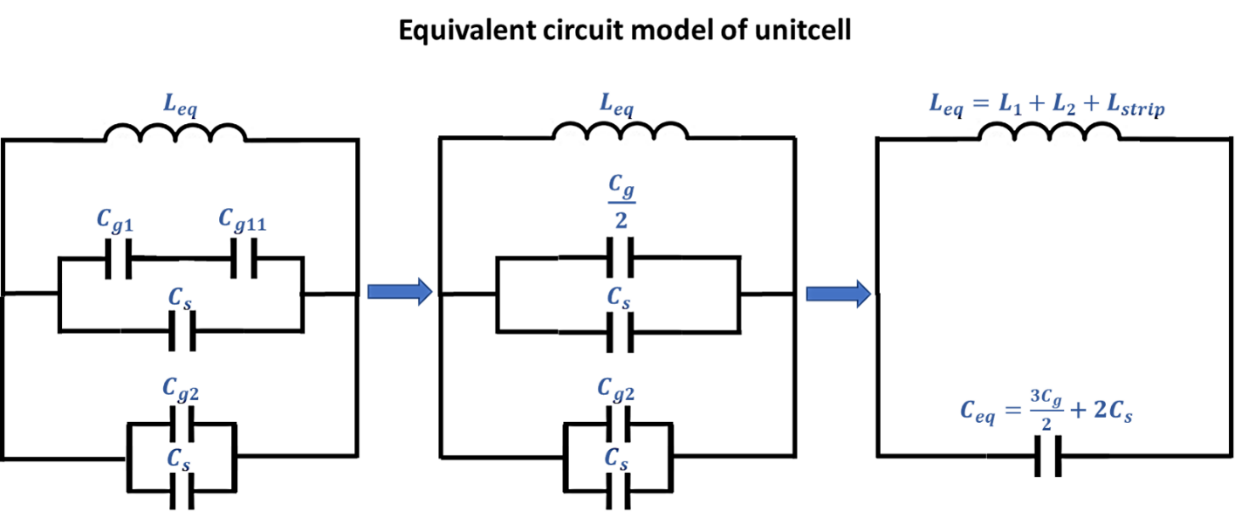


**Figure S1.** Equivalent circuit model progression of the metamaterial unit cell.
In left side the initial schematic showing the gap capacitors along with the inter-ring coupling capacitance and total series inductance​. In center, the simplified model where C_g1_ and C_g11_ are replaced by their series equivalent C_g​/2_, and the capacitance topology is reduced.
In the right side, final lumped-element LC model showing total inductance $L_{eq}= L₁ + L₂ + L_{strip}$and equivalent capacitance $C_{eq} = (\frac{3Cg}{2}) + 2Cs$​, capturing the dominant resonant behavior of the structure.

**S2: Wireless Metamaterial (wMTM) surface**


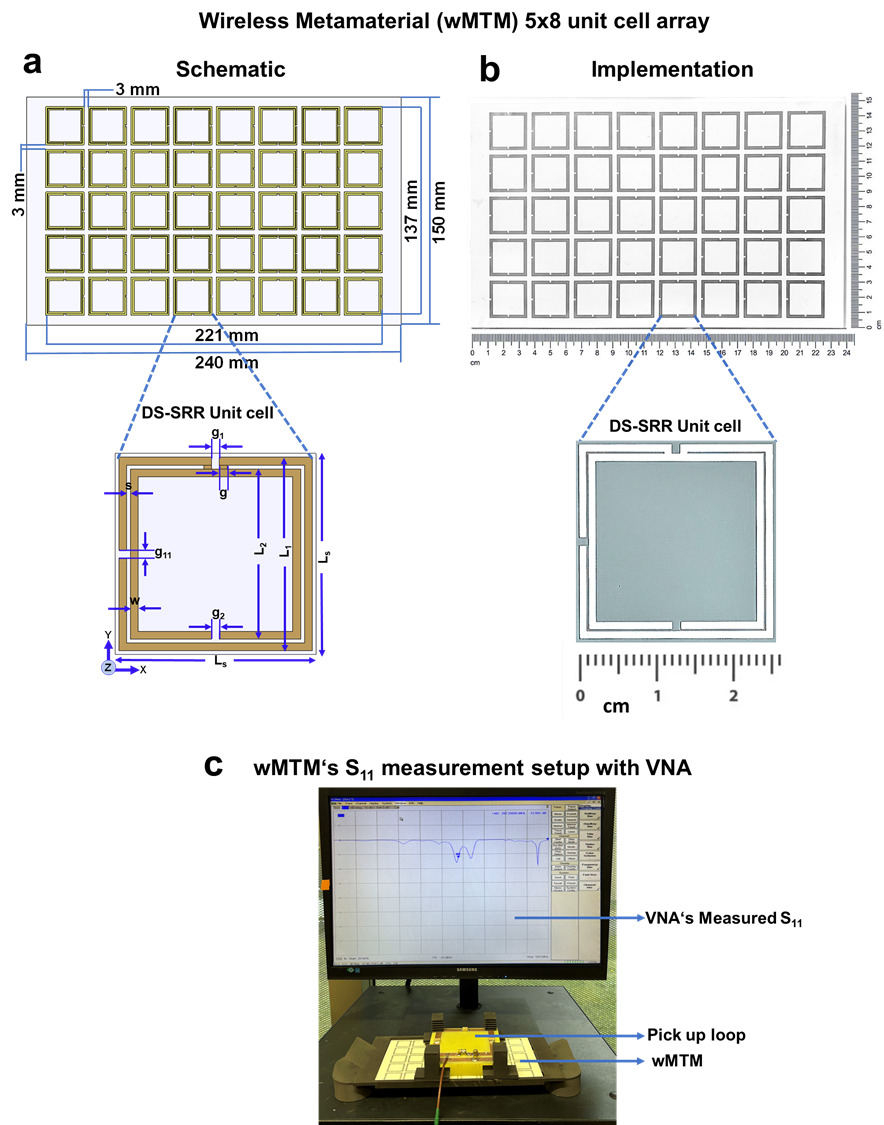


**Figure S2:** (a) Schematic of the wireless metamaterial (wMTM) with a 5 × 8 unit cell array and with zoomed view of the unit cell. (b) Fabricated wMTM on Rogers 4360G2 substrate with a zoomed view of the unit cell. (c) S₁₁ measurement setup of the wMTM using a VNA with a 10 × 10 cm² pickup loop placed over the wMTM.

**S3: Lumped component values and measured S-parameter matrices for planar and bend antennas**


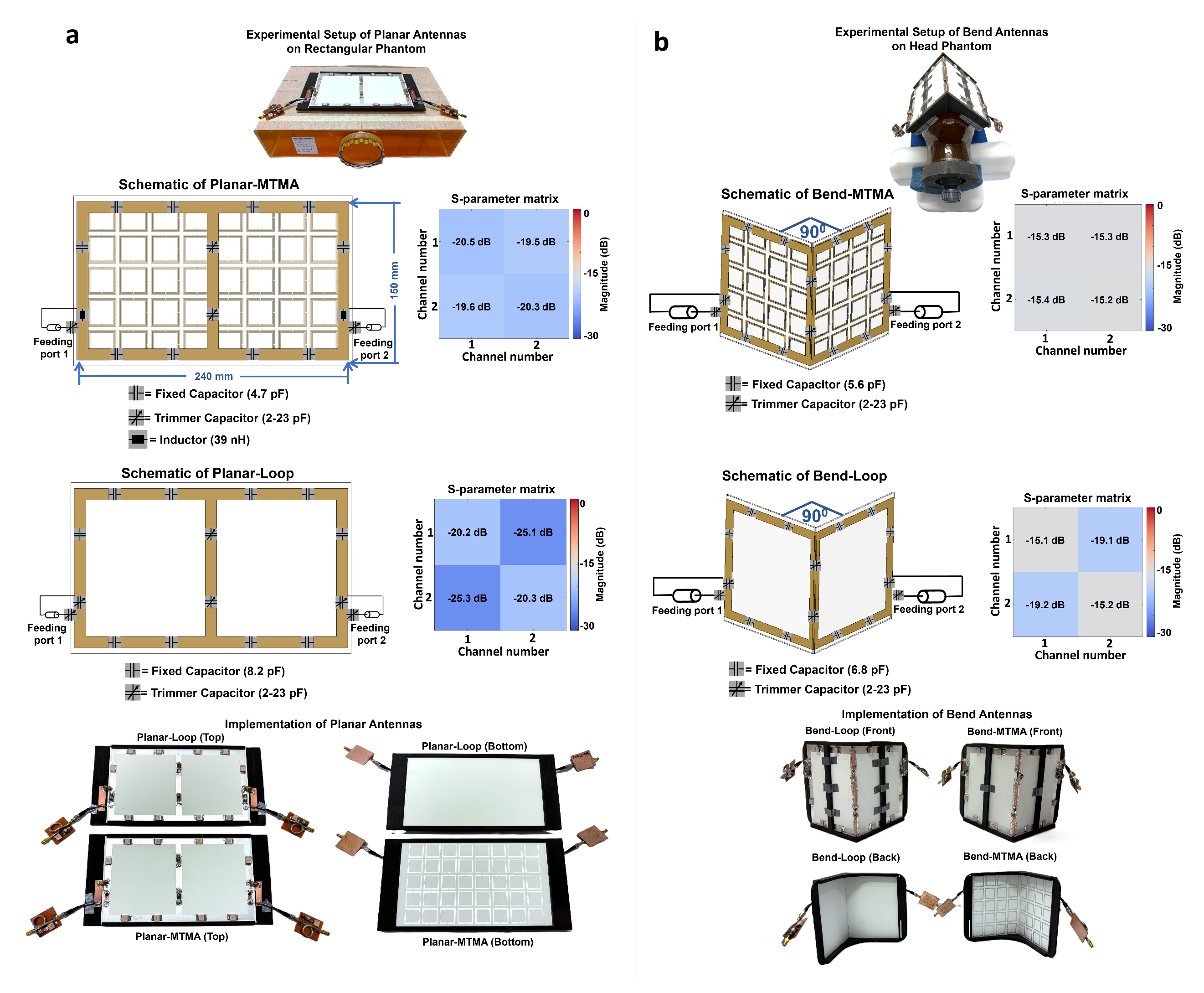


**Figure S3:** Bench measurements and characterization of the scattering parameters (S-parameter) were performed using an 8-channel vector network analyzer (VNA) (ZVT 8; Rohde & Schwarz, Memmingen, Germany) in a frequency range of 200 to 400 MHz. Each 2×2 matrix was measured using VNA and represents the return loss (S₁₁, S₂₂) and inter-channel isolation (S₁₂, S₂₁) coefficients. The heatmap values are presented in decibels (dB), reflecting both mutual coupling and matching characteristics. **(a)** Experimental setup of Planar antennas on rectangular phantom. Schematic layouts with detailed lumped component values and measured S-parameter matrices on rectangular phantom are shown for Planar-MTMA, Planar-Loop configurations. In the head phantom and two volunteers (2 men), the averaged measured S-parameters were S_11_ and S_22_ < –12.2 dB, and S_12_ and S_21_ < –12.5 dB for in vivo occipital lobe imaging. Implemented Planar-MTMA, Planar-Loop with top and bottom view. **(b)** Experimental setup of Bend antennas on anatomically shaped head phantom. Schematic layouts with detailed lumped component values and measured S-parameter matrices are shown for Bend-MTMA, Bend-Loop configurations. Averaged measured S-parameter matrices in the head phantom and in three human volunteers are shown for the Bend-MTMA and Bend-Loop configurations to cover various coil loading conditions for in vivo ocular imaging. Implemented Bend-MTMA, Bend-Loop with front and back view.

**S4: Parametric analysis of the metamaterial unit cell**

To understand how unit cell (UC) geometry influences resonance behavior and the resulting RF field characteristics, we performed an expanded parametric analysis. This included variations in UC size (ring length, gap width, and trace width), evaluation of an MNG-excited variant, a tri-ring configuration, and array-level E-H field simulations to assess how UC geometry shapes the collective electromagnetic response.^[16,17]^  These analyses establish relationships among UC size, field strength, and qualitative near-field reach, factors that collectively influence the effective depth penetration behavior of the metamaterial once it is incorporated into the RF system.^[16,17]^

***S4.1 Unit Cell Size (Ring Length) Variation***

Simulations were performed by varying the outer ring length L_1_ (15, 20, 25, 30 mm) while keeping all other geometric parameters identical to the original UC design. Minor adjustments of the inter-ring spacing s (0.19, 0.20, 0.11, 0.08 mm) were applied to align each geometry with its corresponding resonance to MRI field strength (5.0 T → 210.2 MHz; 7.0 T → 297.2 MHz; 10.5 T → 450 MHz; 14.0 T → 600 MHz). For each variant, the inner ring length L_2_ updated automatically according to the DS-SRR relation L_2_=L_1_−2w−2s. The S-parameter responses (**Figure 4.1a**) show a shift toward lower resonance frequencies with increasing ring length, and a shift toward higher frequencies for smaller ring sizes, in accordance with the LC-controlled behavior of split-ring resonator geometries and the analytical model in S1. In general, the inductance of a split-ring resonator increases with its total ring length, and the analytical model also shows that the inductance increases with the total ring length of the DS-SRR; since the LC resonance frequency (f) varies as f∝1/√(LC), increasing L_1_ naturally shifts the resonance to lower frequencies, in agreement with the simulation results. Retrieval of the effective medium parameters **(Figure 4.1b,c**) confirms that all variants maintain a negative permittivity (ENG) with positive permeability, preserving the electric-dipole dominated resonance response. The electromagnetic field distributions (**Figure 4.1d**) demonstrate that decreasing L_1_ strengthens both the magnetic and electric fields and concentrates the H-field more centrally within the UC. Quantitatively, the smallest UC (15 mm) exhibits the highest mean H-field and E-field, indicating stronger local field confinement compared with the original 25 mm UC. Conversely, the largest UC (30mm) produces weaker but spatially broader fields. These results establish a clear correlation between UC size, resonance tuning, and the resulting E–H field characteristics.

Although the smaller UCs in this design exhibit stronger near-field confinement and could, in principle, be engineered to resonate at ~300 MHz, scaling these geometries to the 7.0 T frequency would require substantial redesign of the capacitive and inductive loading. The 25 mm UC used in this work already lies in the compact regime for 7.0 T metamaterial design and provides an effective compromise between magnetic-field enhancement, manufacturability, and E-field/SAR control. This makes it a suitable and robust choice for integration into MRI coil systems.


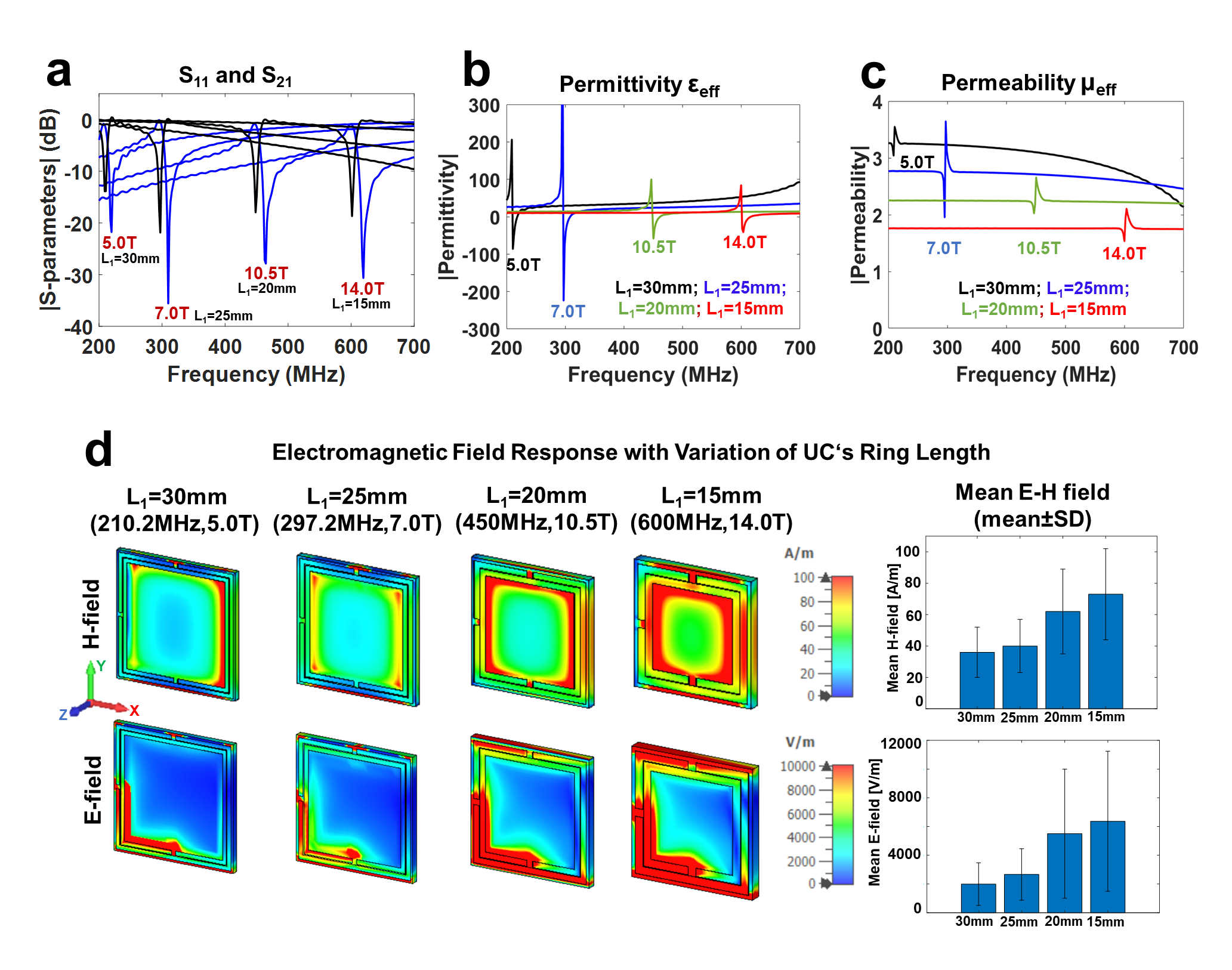


**Figure S4.1:** Parametric variation of UC ring length (L₁) and its influence on resonance behavior and field distributions. (a) S-parameters (S_11_ and S_21_) showing resonance shifts with L₁. (b–c) Retrieved ε_eff_ and μ_eff_ confirming ENG behavior across variants. (d) Simulated H- and E-field distributions for different L₁ values and corresponding mean field strengths in bar plots.

***S4.2 Unit Cell Gap Size Variation***

To assess the resonance response of the UC to its capacitive loading, the gap ‘g’ was varied from 0.5 mm to 25 mm, i.e., g=0.5,1.0,1.5,2.0,2.5 mm while keeping all other geometric parameters constant (**Figure S4.2**). The original design (g=1 mm) resonates at ~300 MHz. Reducing the gap to 0.5 mm increases the capacitance and shifts the resonance to lower frequencies, whereas increasing the gap progressively decreases the capacitance and shifts the resonance to higher frequency than ~300MHz baseline (**Figure S4.2**). Moderate changes in g = 1 to 1.5 mm produce only minor resonance shifts of ~3 MHz, while larger increases (1 to– 2 or 2.5 mm) result in more pronounced upward shifts of ~9 MHz. As expected for DS-SRR structures, gap variation primarily affects the LC resonance without substantially altering the ENG and positive-permeability character of the UC. These simulation results are consistent with the analytical model in **S1**, where the gap size determines part of the total capacitance.

**Figure S4.2:** S₁₁ response for UC gap size variation (g = 0.5, 1.0, 1.5, 2.0, 2.5 mm) and its effect on resonance behavior.

***S4.3 Unit Cell Width Variation***

To evaluate the resonance response of the UC to changes in trace width, ‘w’ was varied from 0.5 mm to 2.0 mm (w=0.5,1.0,1.5,2.0 mm) while keeping all other parameters as same as original UC design (w=1.0 mm) (**Figure S4.3**). Reducing the width to 0.5 mm increased the inductance of the rings and shifted the resonance to a lower frequency than the 300 MHz baseline, whereas increasing the width to 1.5-2.0 mm reduced the inductance and shifted the resonance to a higher frequency than 300 MHz. Inductance decreases as the trace width increases, because a wider strip provides a larger area for current flow and therefore alters the magnetic flux linked with the ring. These modest frequency shifts are consistent with the expected LC behavior of SRR structures and the matched analytical model of **S1**. Variations in width did not alter the ENG mode or the overall field characteristics.

**Figure S4.3:** S₁₁ response for UC width variation (w = 0.5, 1.0, 1.5, 2.0 mm) and its effect on resonance behavior.

***S4.4 Mu-Negative (MNG) Unit Cell Variant***

To examine whether alternative resonance modes alter the electromagnetic response and field behavior, the unit cell was also simulated under boundary conditions that support a magnetic dipole resonance. A PEC boundary was applied along the x-axis and a PMC boundary along the z-axis, with wave propagation along y, enabling excitation of a Mu-negative (MNG) mode. The retrieved effective parameters reveal a negative permeability near ~300 MHz while maintaining positive permittivity, distinguishing this response from the ENG-dominated mode of the original UC **(Figure S4.4a,b)**. Compared with the ENG UC, the MNG excitation produces a distinctly different H-field pattern, exhibiting a more symmetrically rounded distribution inside the UC ring rather than the ring-side-enhanced, dipole-like H-field pattern observed in the ENG configuration. MNG UC shows a ~23% increase in mean H-field, accompanied by a ~30% increase in E-field strength compared to ENG UC **(Figure S4.4a,b)**. These differences in field pattern confirm that the same physical geometry can support distinct resonance mechanisms depending on excitation symmetry. Importantly, the MNG mode demonstrates that modifying the magnetic response of the UC directly alters near-field localization, reinforcing that UC’s effective medium characteristics influence E-H field distribution.


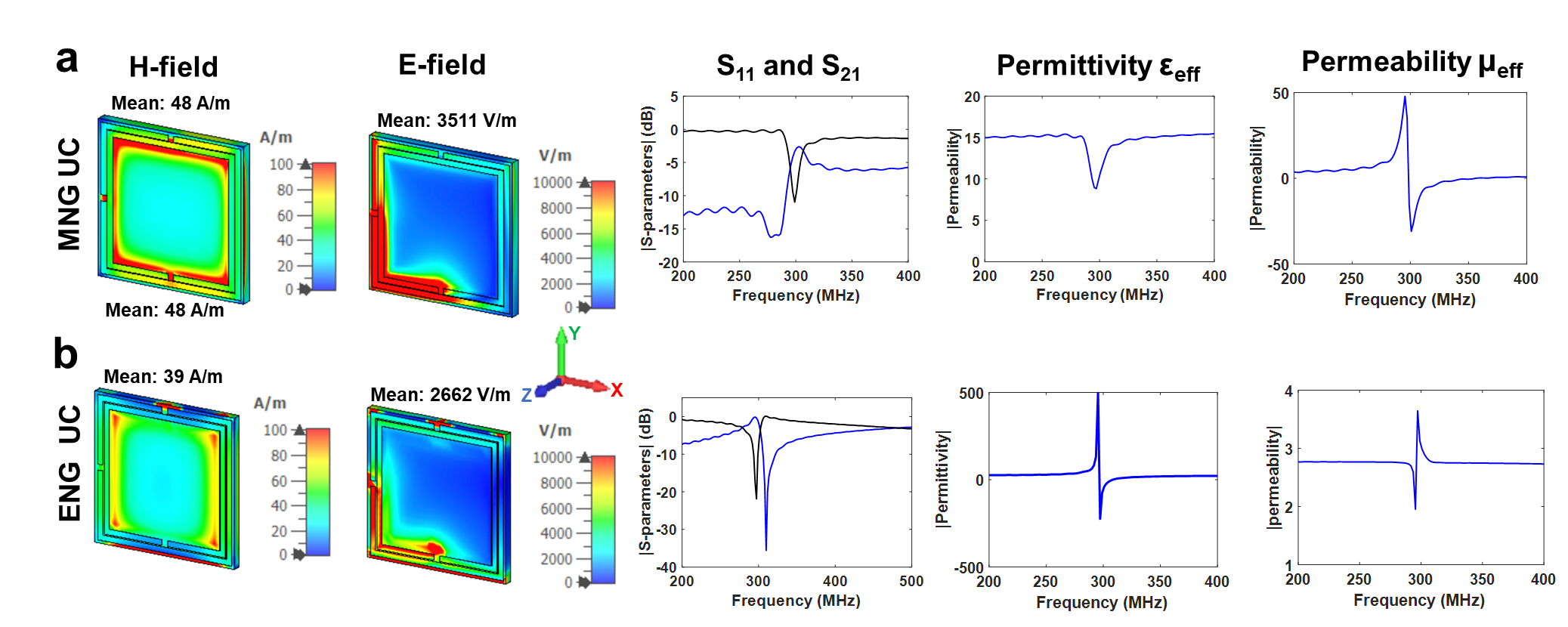


**Figure S4.4:** MNG UC (top) vs. ENG UC (bottom): H- and E-field distributions, S-parameters, and effective medium response. (a) MNG-excited UC exhibiting μ-negative behavior with a modified H/E-field pattern. (b) ENG UC (the original UC used in this study) showing ε-negative behavior for reference.

***S4.5 Tri-Ring Unit Cell Variant***

To explore whether additional inductive-capacitive loading could further enhance the electromagnetic response, a third conductive ring was added to the original UC design **(Figure S4.5a)**. The geometry was adjusted so that the third inner ring length satisfied L_3_=L_2_−2w−2s, and the inter-ring spacing (s=0.3mm) was slightly optimized to maintain resonance at ~300 MHz. The tri-ring UC remains an ENG configuration and is governed by an electric-dipole–driven resonance. As shown in **Figure S4.5**, the tri-ring UC produces a higher mean H-field (+28% relative to the dual-ring UC) while preserving a similar spatial pattern, indicating that the additional ring increases the overall inductive–capacitive coupling of the structure and thereby strengthens both the magnetic and electric fields. However, this benefit comes at the expense of substantially elevated electric fields, i.e., the mean E-field nearly doubles (+98%) and becomes concentrated along the additional gap regions of the third ring. This illustrates a fundamental design trade-off, i.e., while stronger H-fields can support larger coverage or greater field penetration, thereby improving transmit–receive efficiency in MRI applications, excessive E-field concentration is undesirable because it can increase local SAR. These results highlight the importance of balancing magnetic-field enhancement with electric-field control when engineering UCs for safe and efficient MRI performance.


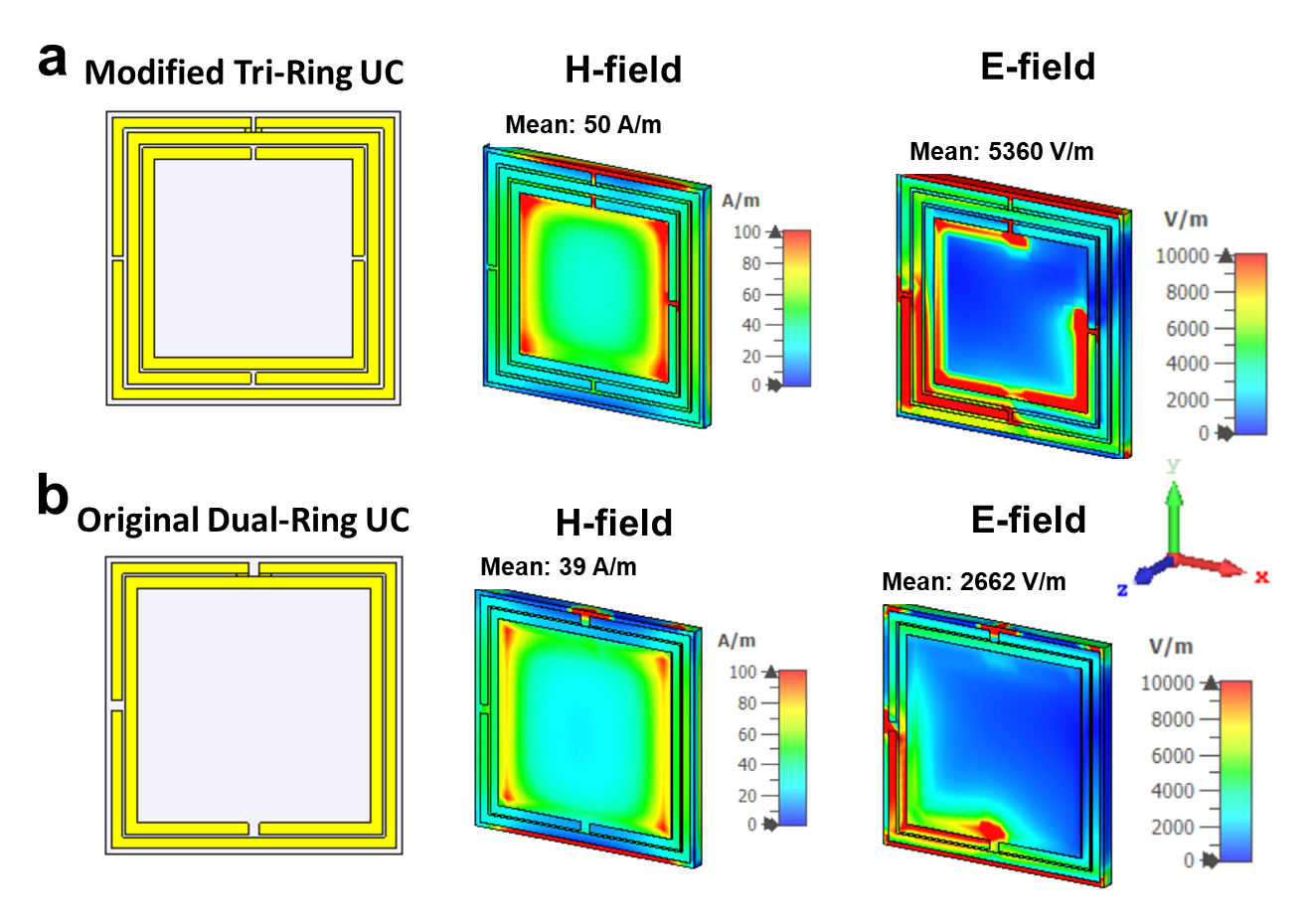


**Figure S4.5:** Comparison of tri-ring and dual-ring UCs. (a) Modified tri-ring UC schematic with corresponding H-field and E-field distributions. (b) Original dual-ring UC and its H-field and E-field shown for comparison.

***S4.6 Unit Cell Characteristics in Arrays***

To connect the UC characteristics to array-level performance, 5×5 ENG UC arrays were simulated using UCs of three L_1_ sizes, i.e., 15 mm (~600 MHz, 14.0 T), 20 mm (~450 MHz, 10.5 T), and the original 25 mm (~300 MHz, 7.0 T) **(Figure S4.6).** When individual UCs are arranged periodically to form a metamaterial surface, they no longer behave as isolated resonators but instead support a collective electromagnetic response across the array. This collective response produces a broad, high-intensity H-field region centered on the array, with a gradual decrease toward the periphery **(Figure S4.6a)**. The resulting field distribution reflects the fundamental collective behavior of the ENG metamaterial surface and is consistent with the dipole-driven resonance of the individual UCs. Arrays constructed from smaller UCs (15 mm and 20 mm) exhibit stronger and more centrally concentrated H-field responses, consistent with their higher local H-field strength at the single-UC level. This behavior is also reflected quantitatively in the mean E- and H-field values extracted from the array simulations, confirming that the array-level trend directly mirrors the unit-cell results **(Figure S4.6a,b)**. To further illustrate how UC geometry influences near-field reach, we evaluated the H-field decay above 5×5 UC arrays constructed from L₁ = 15 mm and L₁ = 25 mm designs. Mean |H| was extracted from transverse Z planes covering the entire array footprint, sampled between z = 0 and z = 5 cm for each UC array. The smaller UC (15 mm) produced a higher surface H-field and shows a slower effective decay, meaning it preserves higher |H| at deeper locations within the first few centimeters (~3cm), consistent with its stronger local field confinement **(Figure S4.6a,c).** It confirms the expected trend that UC-level field strength influences the near-field reach of the metamaterial surface. While this does not directly quantify depth penetration in MRI, it demonstrates the expected trend that UC’s stronger field confinement at the array level can support improved E-H field reach when integrated into the full RF coil–sample system of MRI. These array-level simulations demonstrate how UC’s size directly shapes the collective E-H field distribution and thereby can influence the effective penetration depth behavior.


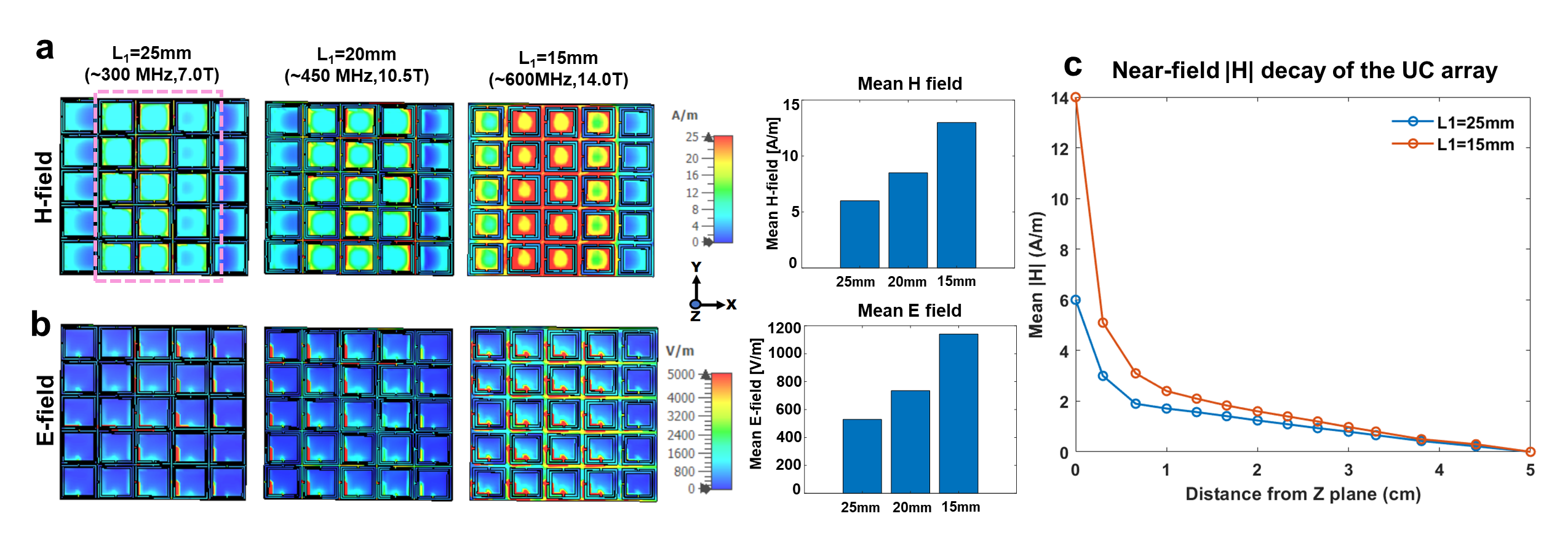


**Figure S4.6:** Effect of UC size on array-level (metamaterial surface) electromagnetic response. (a) H-field maps of 5×5 arrays constructed from L₁ = 25 mm, 20 mm, and 15 mm UCs, with corresponding mean H-field in the bar plot. The pink rectangle shows the central region of the array. (b) E-field maps of the same arrays and their mean E-field in the bar plot. (c) Mean H-field near field decay with distance for arrays constructed from L_1_ = 15 mm and 25 mm UCs. Smaller UC (L_1_=15mm) generate stronger surface H-fields and exhibit a slightly slower near-field effective decay, consistent with enhanced local field confinement.

***S4.7 H-field characteristic*** ***of Planar and Bend Antennas***

The array-level simulations demonstrate that when UCs are assembled into an array, the resulting MTM surface supports a collective electromagnetic response characterized by a broader, centrally intensified H-field. In MRI, such central H-field enhancement is beneficial for enabling stronger signal and greater depth coverage. ^[16,17]^ However, depth penetration in MRI is determined by the complete RF system, i.e., the transmit–receive coil, the metamaterial layer, sample loading, and the associated detuning, matching, and loss mechanisms. The array-level simulations presented here demonstrate that variations in UC’s geometry and size influence the E-H field strength and spatial extent of the collective near-field generated by the array and its near-field reach.

To directly quantify this effect at the antenna level, the H-field decay inside the rectangular phantom (phantom height 100 mm) was evaluated using full-array CST simulations **(Figure S4.7a,b)**. Consistent with previous metamaterial reports, the results demonstrate that the Planar-MTMA exhibits a slower effective decay, preserving higher |H| at deeper locations than the Planar-Loop **(Figure S4.7b),** consistent with the enhanced depth penetration provided by the integrated metamaterial..^[16,17]^ As shown in **Figure S4.7b**, the Planar-MTMA delivers approximately ~43% higher surface |H|, maintains higher field strength throughout the phantom depth, and exhibits a slower effective decay than the Planar-Loop, with both curves converging only near ~100 mm. Furthermore, the imaginary component of the H-field reflects reactive near-field energy storage. The Planar-MTMA exhibits a substantially larger imaginary H-field than the Planar-Loop **(Figure S4.7b, imaginary H-field)**, consistent with the capacitive and inductive response of the ENG DS-SRR UC array. This elevated reactive field strengthens near-field evanescent coupling and contributes to the increased overall |H|-field magnitude observed with the MTMA. Overall, the H-field behavior is characteristic of an ENG-based metamaterial integrated antenna, which concentrates fields near the surface and enhances effective penetration depth, consistent with the experimentally measured B₁⁺ and GRE signal-intensity 1D profiles obtained in the rectangular phantom in **Figure 4**. In addition, we evaluated the H-field distribution of the Bend-MTMA and Bend-Loop antennas placed on a square phantom (height 160 mm) with electrical properties identical to those of the rectangular phantom used for the Planar antennas **(Figure S4.7c)**. Bend antennas produce two dominant lateral H-field lobes rather than a single central lobe as Planar antennas; therefore, a horizontal 1D line was taken across the phantom width at ~mid-height (**Figure S4.7c**) for H-field computation. The Bend-MTMA exhibits consistently higher |H|, real(H), and imaginary(H) across both lobes compared with the Bend-Loop antenna, reflecting stronger near-field coupling and enhanced effective depth penetration along the lateral directions (**Figure S4.7d**). These results are consistent with the planar antenna findings and demonstrate that the wMTM layer preserves its near-field enhancement capability under bending. The results of Bend antennas are consistent with the experimentally measured B₁⁺ enhancement and improved anatomical coverage for ocular imaging. Furthermore, the array simulations demonstrate that UC size governs the resulting E–H field intensity and the shape of the collective mode supported by the MTM surface. This collective mode couples efficiently to the integrated loop coil via near-field evanescent interaction, enabling the stronger signal and improved coverage in depth achieved with the MTMA design. This behavior is confirmed in the array simulations and validated experimentally in phantom and in vivo B₁⁺ maps. The enhancement manifests as improved B₁⁺, stronger signal in deeper regions of the sample (phantom), and extended anatomical coverage for example, across the orbit, paranasal sinuses, and anterior brain, as well as in the occipital region.


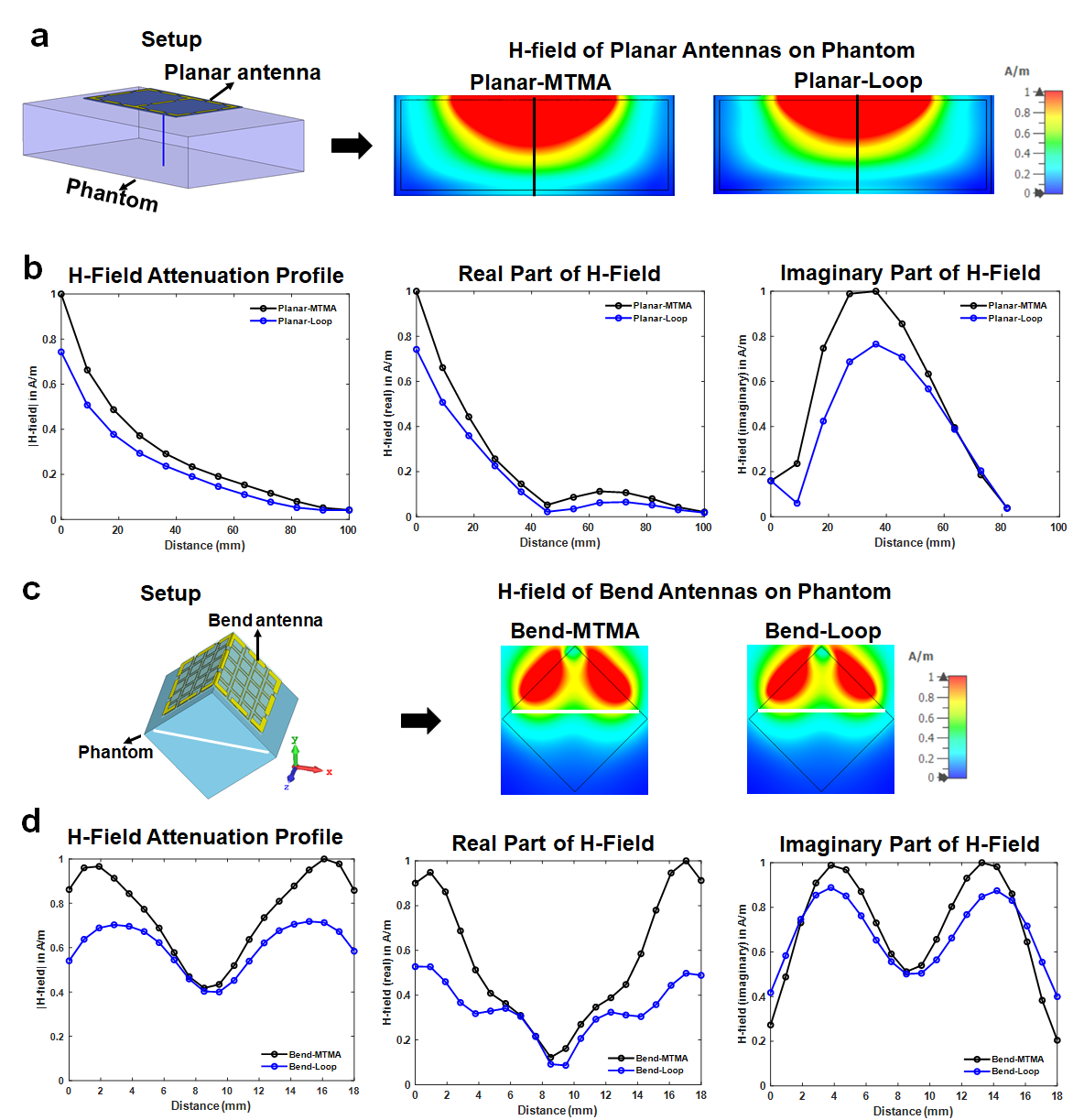


**Figure S4.7:** **(a)** Simulation setup showing the planar antenna configuration placed on top of the rectangular block phantom. The blue vertical line indicates the 100 mm phantom height along which the H-field was evaluated. Representative |H|-field maps at the phantom center are shown for the Planar-MTMA and the Planar-Loop. **(b)** H-field attenuation 1D profile inside the phantom (0–100 mm depth) with real and imaginary parts of the H-field for Planar-MTMA vs Planar-Loop. The H-field values were normalized to their respective peak magnitudes (0–1). **(c)** Simulation setup showing the bend antenna configuration placed on top of the square block phantom. The white horizontal 1D line indicates ~mid-phantom height along which the H-field was evaluated at the phantom center. Representative |H|-field maps at the phantom center are shown for the Bend-MTMA and the Bend-Loop. **(d)** H-field attenuation 1D profile inside the phantom with real and imaginary parts of the H-field for Bend-MTMA vs Bend-Loop. The H-field values were normalized to their respective peak magnitudes (0–1).

# S5: Equivalent Impedance Model of the MTMA

## To model near-field interaction in the metamaterial antenna (MTMA) system, the active loop coil array (top layer) and passive wireless metamaterial (wMTM) comprising an 5x8 array of 40 DS-SRR unit cells (bottom layer) are treated as resonant subsystems separated by a dielectric substrate (Rogers 4360G2, 1.52 mm, εr ≈ 6.15). Each is represented by a complex impedance, with mutual coupling captured by a shared impedance term. The system is described as a 2-port network using a Z-parameter matrix:^[14]^

## The impedance definitions are given below:

- Z_11_​=Z_1_​ (loop array’s self-impedance)
- Z_22​=_Z_2_​ (wMTM’s self-impedance)
- Z_12_​=Z_21_​=Z_mutual_​ (mutual impedance across the substrate)

$$Z_{1}=R_{1}+j\omega L_{1}+\frac{1}{j\omega C_{1}}$$

$$Z_{2}=R_{2}+j\omega L_{2}+\frac{1}{j\omega C_{2}}$$

$$Z_{12}=j\omega M+\frac{1}{j\omega C_{substrate}}$$

Here, C_substrate_​ can be estimated using a parallel plate approximation: ^[14]^

$$C_{\text{substrate}}=\frac{\varepsilon_{r}\varepsilon_{0}A}{d}$$

- A: Effective overlap area of DS-SRR cells and loop trace
- d=1.52 mm: Rogers 4360G2 substrate’s thickness
- ε_r​_=6.15: dielectric constant of Rogers 4360G2

Using Kirchhoff's voltage law for a two-port system with mutual coupling, the loop-driven system satisfies:

$V_{1}=Z_{1}I_{1}+Z_{12}I_{2}$ (1)

$V_{2}=Z_{12}I_{1}+Z_{2}I_{2}$ (2)

As the wMTM (DS-SRR array) is a passive receiver (i.e., V₂ = 0), we solve the equation (2) for I₂:

$$0=Z_{12}I_{1}+Z_{2}I_{2}$$

$$I_{2}=-\frac{Z_{12}}{Z_{2}}I_{1}$$

Substitute into the equation (1):

$$V_{1}=Z_{1}I_{1}-\frac{Z_{12}^{2}}{Z_{2}}I_{1}$$

$$I_{1}=\frac{V_{1}}{Z_{1}-\frac{Z_{12}^{2}}{Z_{2}}}$$

$$I_{2}=-\frac{Z_{12}}{Z_{2}}\cdot\frac{V_{1}}{Z_{1}-\frac{Z_{12}^{2}}{Z_{2}}}$$

## Hence, current induced in wMTM layer is given by below

$$I_{2}=-\frac{Z_{12}}{Z_{2}}\cdot\frac{V_{1}}{Z_{1}-\frac{Z_{12}^{2}}{Z_{2}}} (3)$$

## *Simplified Resonance Criterion for Maximum Coupling*

To determine the condition under which the wMTM layer receives maximum energy from the loop coil, we analyze the denominator of the induced current expression of equation (3): The induced current |I₂| is maximized when the denominator approaches zero, i.e.:

$Z_{1}-\frac{Z_{12}^{2}}{Z_{2}}\approx0$ when, Z₁·Z₂ ≈ Z₁₂²

This derivation shows that the induced current in the wMTM depends on both the coupling impedance and the impedance matching between the two layers. Near resonance, where impedance conditions align, I_2_​ maximized, resulting in constructive field reinforcement and enhanced receive sensitivity, as supported by simulations and experimental data.

**Note:** This equivalent impedance model treats the DS-SRR array as a unified resonant surface and does not resolve individual unit cell’s currents. While sufficient for first-order coupling analysis and system-level predictions, the model can be expanded to incorporate distributed DS-SRR currents or full electromagnetic interactions in future work.


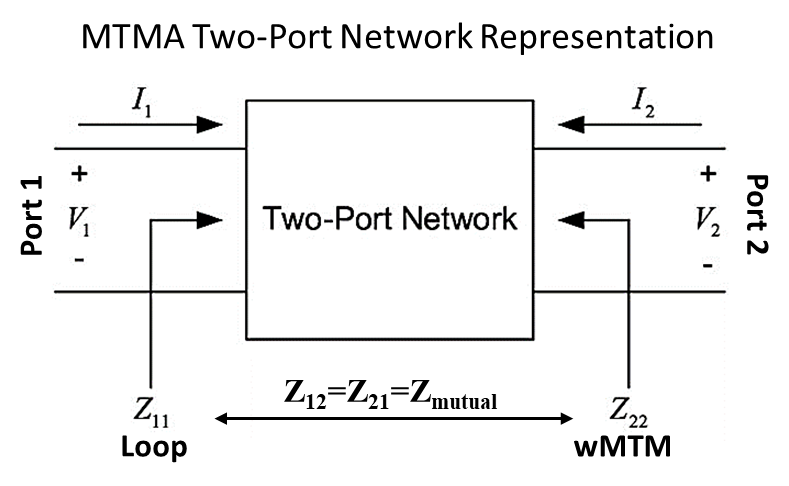


**Figure S4:** The schematic of the MTMA as a **2-port impedance model**, showing:

**Top layer loop array** (Port 1, with impedance Z_11​=_Z_1_); **wMTM** (Port 2, with Z_22_​=Z_2_); **Mutual impedance** Z_12_​=Z_21_​=Z_mutual​_; voltage source V_1_​ applied at Port 1.

**S6: Parallel Imaging Performance of Planar and Bend Antennas in Accelerated MRI**

Parallel imaging (PI) performance was evaluated to confirm that the MTMA preserves the sensitivity and channel independence required for accelerated MRI acquisition. ^[18-29]^

***Methods***

PI scans were performed using Siemens’ integrated Parallel Acquisition Techniques (iPAT) package, employing GRAPPA (Generalized Autocalibrating Partially Parallel Acquisition; k-space–based reconstruction) and mSENSE (modified SENSE; image-domain reconstruction) on the MAGNETOM 7.0 T MRI scanner.^[25-29]^ Rectangular block phantom PI scans were acquired using the Planar-MTMA and Planar-Loop configurations, while head-phantom PI scans were performed using the Bend-MTMA and Bend-Loop configurations. All PI experiments used an acceleration factor of R = 2 with 24 reference ACS lines (phase encoding: left–right). All remaining sequence parameters matched those of the GRE-FLASH protocol used in the respective phantom experiments (see Protocol Table S1). With R = 2, the scan time was reduced from 6.26 to 3.26 min for the rectangular phantom and from 4.18 to 2.18 min for the head phantom. PI performance comparisons between MTMA and Loop antennas are shown in **Figure S6.1-S6.2**. For PI reconstructions, the evaluation focused on the axial slice in which the L–R phase-encoding direction makes acceleration effects most visible. Mean signal was evaluated within the regions of interest (ROI) used for receive-sensitivity analysis (ROI-1). Image noise graininess was quantified in a second ROI (ROI-2), placed in visually low-signal regions of each phantom, using a fixed 5×5-pixel local standard-deviation window applied across ROI-2.^[22-24]^ Exported noise-correlation matrices were presented in **Figure S6.1-S6.2** to assess channel independence for each antenna configuration.

***Results***

With GRAPPA (R = 2), both the Planar-MTMA and Planar-Loop antennas exhibited the expected mild L–R shading with no reconstruction instabilities **(Figure S6.1)**. The Planar-Loop showed a more pronounced top-edge (right) mid-main lobe shading (black arrow) distortion compared with the Planar-MTMA. With mSENSE (R = 2), the Planar-MTMA appeared more distorted along the side of the main lobe (black arrow), as its higher received signal reveals reconstruction texture in regions where the Planar-Loop has little to no signal, making these effects essentially invisible in the Planar-Loop. The mean signal intensity (ROI-1), shown in the corresponding bar plots, confirmed that the Planar-MTMA consistently maintained its higher receive sensitivity, with percentage increases over the Planar-Loop closely reproducing the improvements previously measured in rectangular phantom experiments with the planar antennas without PI **(Figure S6.1b)**. Across both GRAPPA and mSENSE, the Planar-MTMA maintained a smooth image appearance with minimal noise graininess, whereas the Planar-Loop exhibited more apparent graininess and stronger propagation of noise in low-signal regions (ROI-2). Relative to the no-PI scan image, the Planar-MTMA showed almost no visible noise amplification, with a minor increase in noise graininess (~1.5% with GRAPPA and ~3% with mSENSE) in ROI-2, whereas the Planar-Loop exhibited higher noise graininess in ROI-2 (~4% with GRAPPA and ~14% with mSENSE) **(Figure S6.1b)**. These results indicate that the enhanced receive sensitivity of the Planar-MTMA contributes to stable PI performance by tolerating the typical undersampling-related noise amplification **(Figure S6.1b)**. Overall artifact patterns were comparable across antennas, confirming that the MTMA does not disrupt PI encoding and reconstructs reliably under both GRAPPA and mSENSE, while maintaining its higher receive signal. Uncombined channel maps (no PI and GRAPPA R = 2) confirm that the MTMA preserves distinct receive-sensitivity profiles for both elements of the 2-channel array (Channel-1 and Channel-2), ensuring stable parallel-imaging reconstruction **(Figure S6.1c)**. In contrast, the Planar-Loop array also maintains distinct channel profiles but with substantially lower receive sensitivity, which contributes to the higher noise amplification observed in its PI reconstructions. Noise-correlation matrices demonstrated good channel independence for both planar antenna configurations **(Figure S6.1d)**.


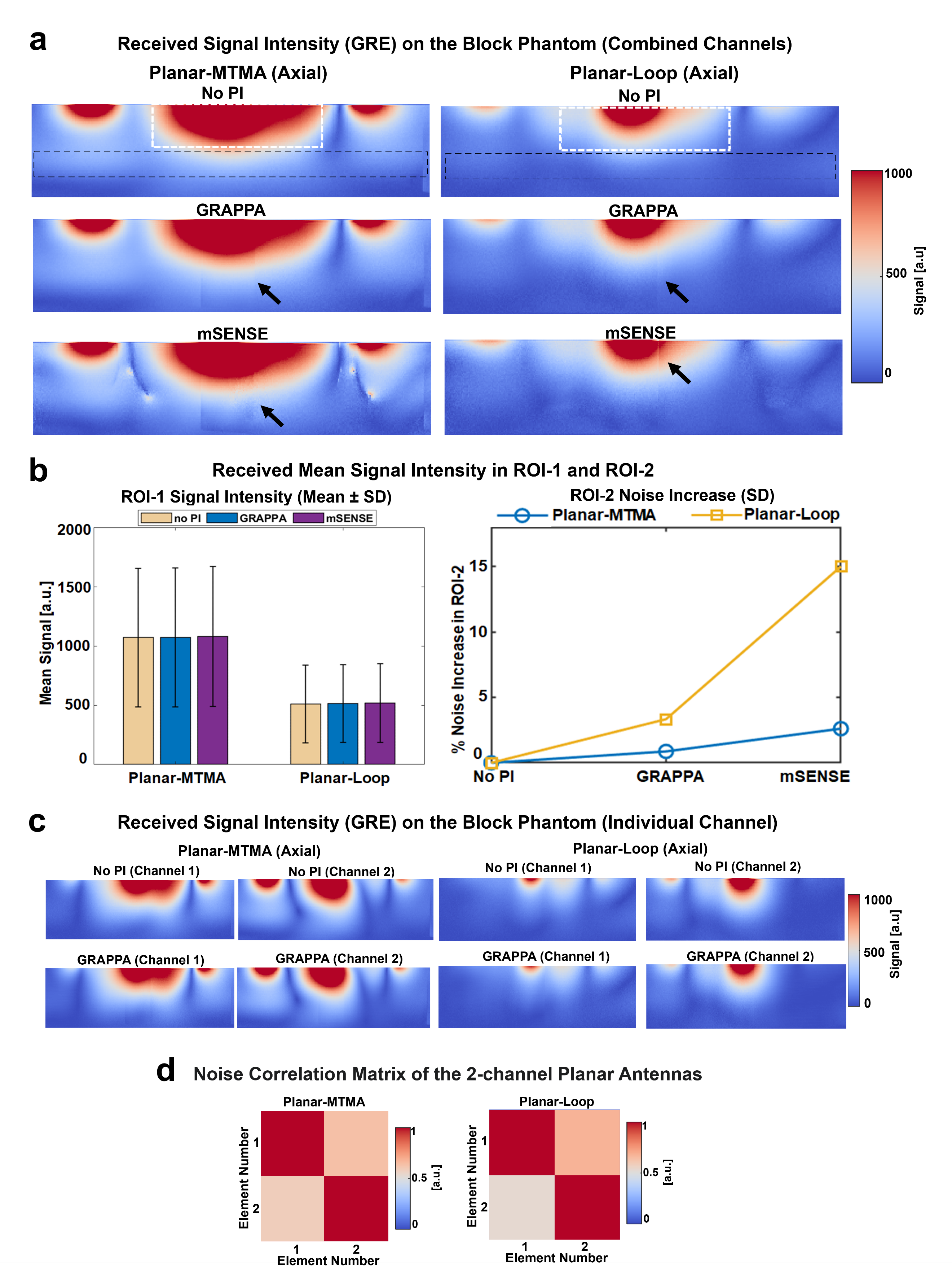


**Figure S6.1:** Parallel Imaging Performance of Planar Antennas in Accelerated MRI. A cool–warm colormap was chosen to enhance the visibility of PI-related signal differences, which are less visible in grayscale. (a) GRE signal intensity maps (axial slice) on the rectangular phantom (combined channels) for the Planar-MTMA and Planar-Loop under no PI, GRAPPA, and mSENSE acceleration. (b) Mean signal intensity bar plot of ROI-1 corresponds to the white dashed rectangle positioned within the main lobe of highest signal intensity for no PI, GRAPPA, and mSENSE. Noise graininess increase analysis of the 1D plot of ROI-2 is placed in visually low-signal regions (black dashed rectangle) of the phantom to assess PI-related noise amplification. (c) GRE signal maps (axial slice) from individual channels under no PI and GRAPPA for Planar-MTMA and Planar-Loop. (d) Noise-correlation matrices of the 2-channel Planar-MTMA and Planar-Loop.

For the Bend-MTMA, PI effects were also evaluated in axial slices where the L–R phase-encoding direction makes acceleration behavior most apparent **(Figure S6.2a)**. With GRAPPA (R = 2), only very mild lateral shading (black arrow) was observed outside the main head-phantom FOV, with no structural distortion and stable reconstruction across both MTMA and Loop arrays. With mSENSE (R = 2), peripheral periodic overfolding (black arrow) outside the head region appeared, which is a classic SENSE artifact pattern, yet the head shape, central FOV, and overall anatomy remained unaffected. Importantly, the Bend-MTMA maintained its higher receive sensitivity and showed no PI-related collapse, confirming full compatibility with both GRAPPA and mSENSE. The mean signal intensity (ROI-1), shown in the corresponding bar plots, confirmed that the Bend-MTMA consistently maintained its higher receive sensitivity, with percentage increases over the Bend-Loop closely reproducing the improvements previously measured in the bend configurations without PI **(Figure S6.2b)**. Only a minimal increase in image noise graininess (≈1-2% in ROI-2) was observed between the Bend-MTMA and Bend-Loop within the head-phantom FOV, indicating that PI-related noise amplification remained negligible for both antennas **(Figure S6.2b)**. The uncombined channel maps also confirm that the Bend-MTMA preserves the distinct sensitivity profiles of each channel required for PI and maintains higher receive sensitivity than the Bend-Loop **(Figure S6.2c)**. Noise-correlation matrices demonstrated good channel independence for both bend antennas, similar to the planar case **(Figure S6.2d)**.

Overall, for both planar and bend antennas, ROI-1, located in the region of maximal signal intensity, the mean signal remained stable across no-PI and PI (GRAPPA, mSENSE) conditions for both antennas, which allowed the MTMA’s percentage signal gain over the Loop to remain consistent across all antenna configurations. These phantom results (no PI) also demonstrate repeatability for both the planar and bend antenna configurations, as confirmed by the consistent ROI-1 mean signal values across the rectangular and head phantoms. Overall artifact patterns were comparable across antennas, confirming that the MTMA reconstructs reliably under both GRAPPA and mSENSE and maintains its higher receive sensitivity than the Loop for both the planar and bend configurations.


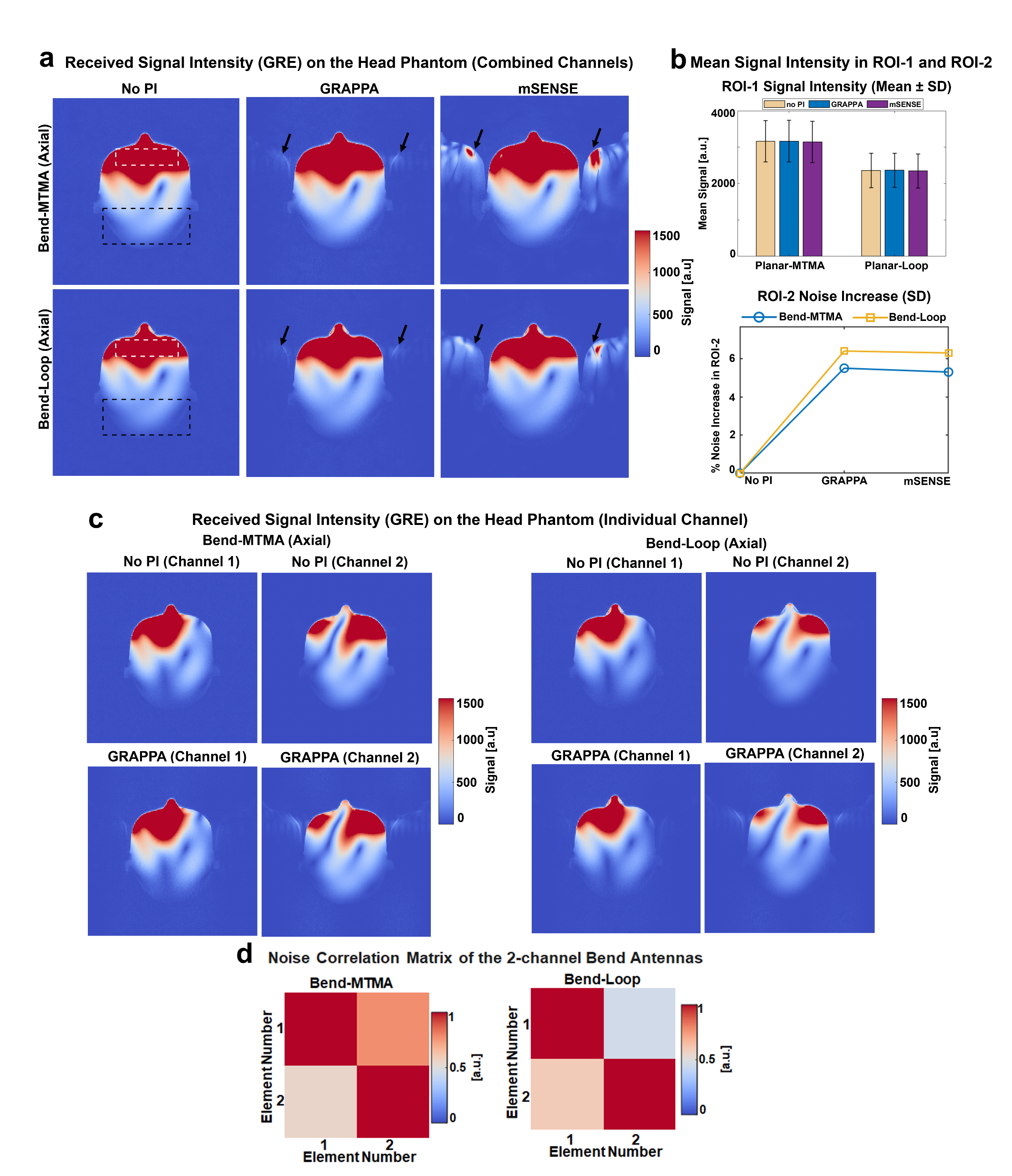


**Figure S6.2:** Parallel Imaging Performance of Bend Antennas in Accelerated MRI. A cool–warm colormap was chosen to enhance the visibility of PI-related signal differences, which are less visible in grayscale. (a) GRE signal intensity maps (axial slice) on the head phantom (combined channels) for the Bend-MTMA and Bend-Loop under no PI, GRAPPA, and mSENSE acceleration. (b) Mean signal intensity bar plot of ROI-1 corresponds to the white dashed rectangle positioned within the front region of the head phantom mimicking the ‘in vivo eye ROI’ of the highest signal intensity for no PI, GRAPPA, and mSENSE. Noise graininess increase analysis of the 1D plot of ROI-2 is placed in visually low-signal regions (black dashed rectangle) of the head phantom to assess PI-related noise amplification. (c) GRE signal maps (axial slice) from individual channels under no PI and GRAPPA of Bend antennas. (d) Noise-correlation matrices of the 2-channel Bend-MTMA and Bend-Loop.

**S7: Surface current of unit cell and the wMTM surface**


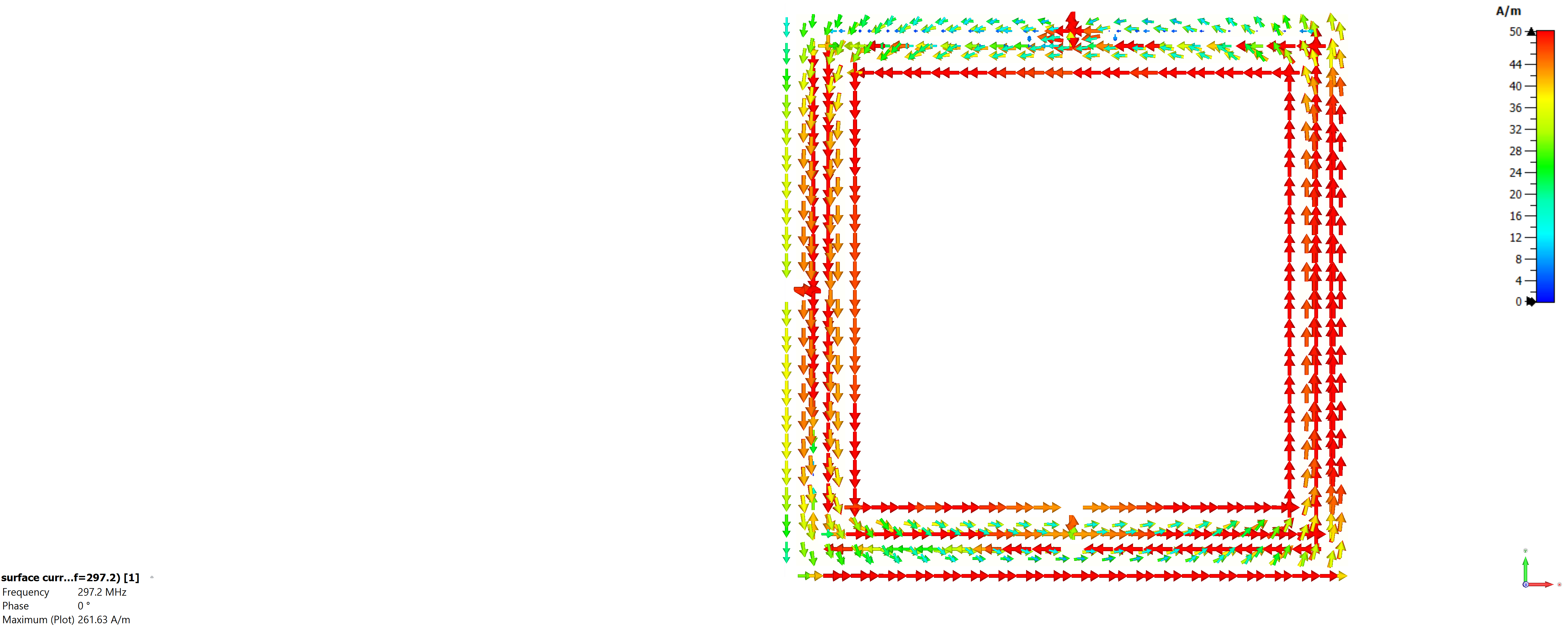

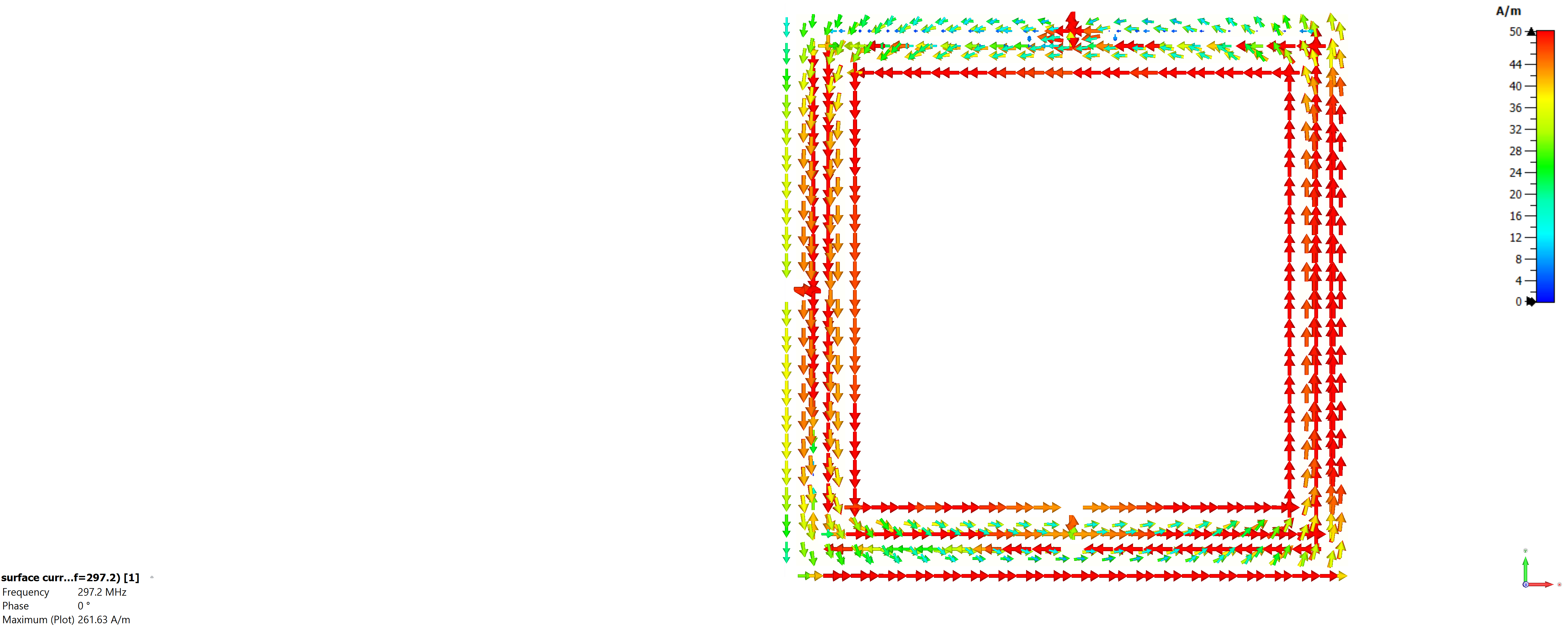


Figure S7.1: Surface current of the DS-SRR unit cell.


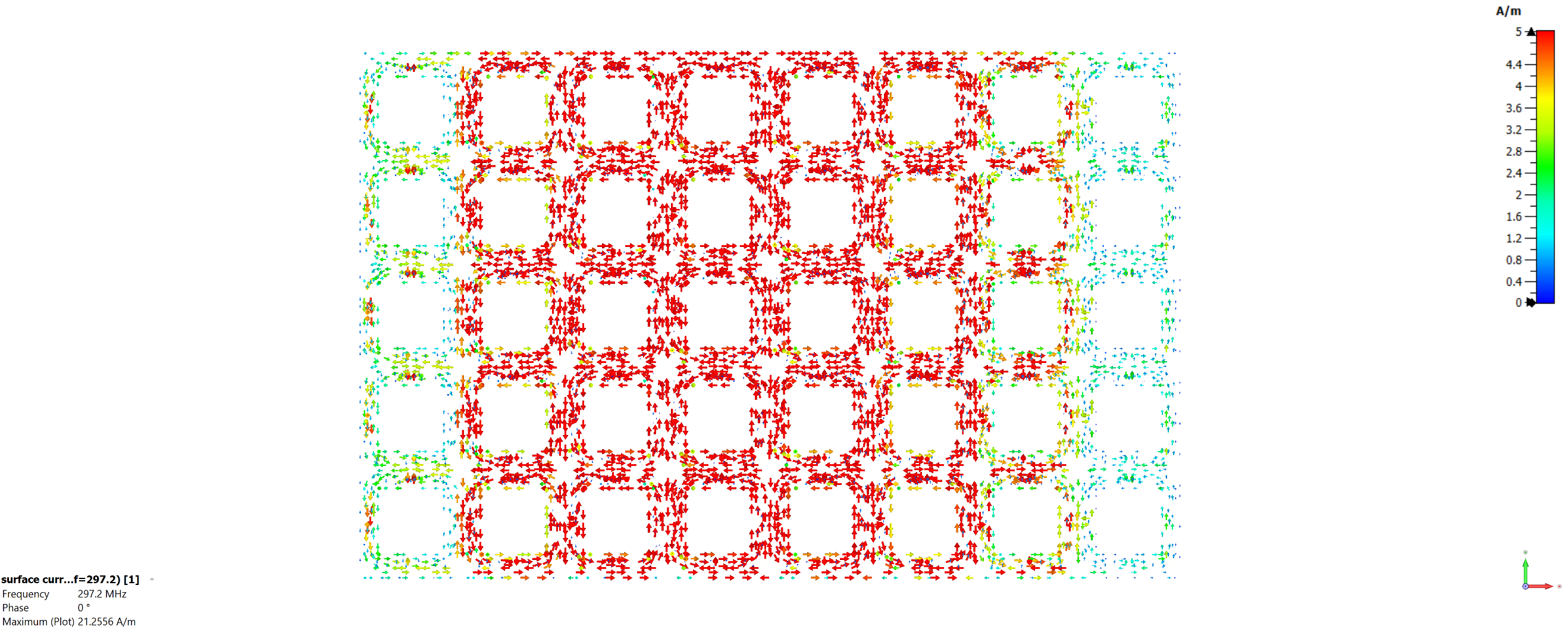

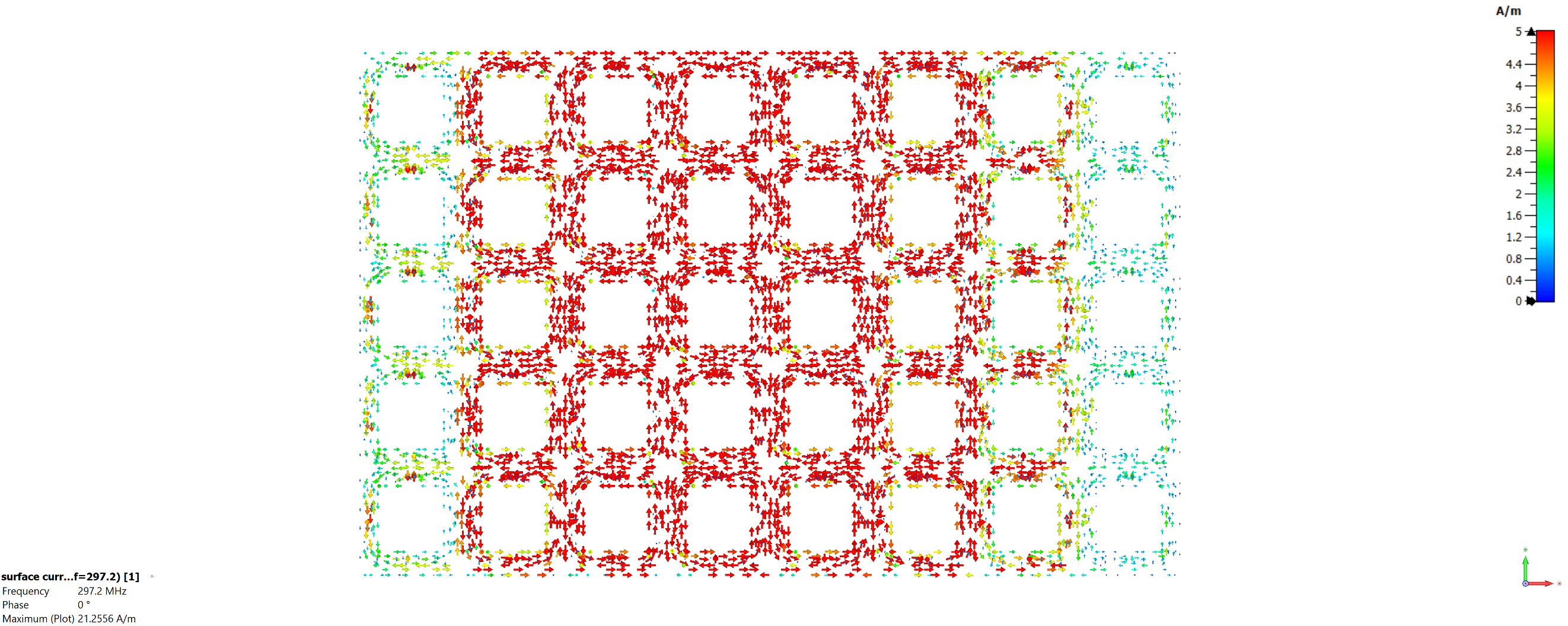


Figure S7.2: Surface current of the wMTM surface.

**References**

1. Saha, C.; Siddiqui, J.Y.; Guha, D.; Antar, Y.M.M. Square Split Ring Resonators: Modelling of resonant frequency and polarizability. In Proceedings of the 2007 IEEE Applied Electromagnetics Conference (AEMC), 19-20 Dec. 2007, 2007; pp. 1-3.
2. C. Saha, J. Y. Siddiqui and Y. M. M. Antar, "Square split ring resonator backed coplanar waveguide for filter applications," 2011 XXXth URSI General Assembly and Scientific Symposium, Istanbul, Turkey, 2011, pp. 1-4, doi:10.1109/URSIGASS.2011.6050665.
3. S. Linden et al., "Photonic Metamaterials: Magnetism at Optical Frequencies," in IEEE Journal of Selected Topics in Quantum Electronics, vol. 12, no. 6, pp. 1097-1105, Nov.-dec. 2006, doi: 10.1109/JSTQE.2006.880600.
4. Moghbeli, E.; Askari, H.R.; Forouzeshfard, M.R. Analyzing the effect of geometric parameters of double split ring resonator on the effective permeability and designing a cloak of invisibility in microwave regime. *Applied Physics A* **2018**, *124*, 361, doi:10.1007/s00339-018-1774-3.
5. Baena, J.D.; Bonache, J.; Martin, F.; Sillero, R.M.; Falcone, F.; Lopetegi, T.; Laso, M.A.G.; Garcia-Garcia, J.; Gil, I.; Portillo, M.F.; et al. Equivalent-circuit models for split-ring resonators and complementary split-ring resonators coupled to planar transmission lines. *IEEE Transactions on Microwave Theory and Techniques* **2005**, *53*, 1451-1461, doi:10.1109/TMTT.2005.845211.
6. Lahiri, B.; McMeekin, S.G.; Khokhar, A.Z.; De La Rue, R.M.; Johnson, N.P. Magnetic response of split ring resonators (SRRs) at visible frequencies. *Opt Express* **2010**, *18*, 3210-3218, doi:10.1364/OE.18.003210.
7. Durán-Sindreu, M.; Naqui, J.; Bonache, J.; Martín, F. Split rings for metamaterial and microwave circuit design: A review of recent developments (Invited paper). *International Journal of RF and Microwave Computer-Aided Engineering* **2012**, *22*, 439-458, doi:https://doi.org/10.1002/mmce.20635.
8. Bilotti, F.; Toscano, A.; Vegni, L. Design of Spiral and Multiple Split-Ring Resonators for the Realization of Miniaturized Metamaterial Samples. *IEEE Transactions on Antennas and Propagation* **2007**, *55*, 2258-2267, doi:10.1109/TAP.2007.901950.
9. All About Circuits. (n.d.). Rectangle Loop Inductance Calculator. Retrieved August 31, 2025, from https://www.allaboutcircuits.com/tools/rectangle-loop-inductance-calculator/
10. Caloz, C. I., & Itoh, T. (2005). Electromagnetic metamaterials: Transmission line theory and microwave applications: The engineering approach. Wiley–IEEE Press.
11. M. T. Thompson, “Inductance calculation techniques—Part I: Classical methods,” Power Control and Intelligent Motion, vol. 25, no. 12, pp. 40–45, Dec. 1999.
12. M. T. Thompson, “Inductance calculation techniques—Part II: Approximations and handbook methods,” Power Control and Intelligent Motion, vol. 26, no. 1, pp. 4–11, Jan. 2000.
13. Seetharaman, S. S., King, C. G., Hooper, I. R., & Barnes, W. L. (2017). Electromagnetic interactions in a pair of coupled split-ring resonators. Physical Review B, 96(8), 085426. https://doi.org/10.1103/PhysRevB.96.085426
14. D. M. Pozar, Microwave Engineering, 4th ed. Hoboken, NJ, USA: John Wiley & Sons, 2011. P. Lorrain and R. Dale, “Electric circuits C: Mutual and self-inductance,” in Electromagnetic Fields and Waves: Including Electric Circuits. New York, NY, USA: W. H. Freeman & Co., 1988.
15. Inductance of flat wire. Terman, F. E. (1945). Radio engineers’ handbook. New York, NY: McGraw-Hill.
16. Ke Wu, Xia Zhu, Xiaoguang Zhao, Stephan W. Anderson, Xin Zhang. Conformal Metamaterials with Active Tunability and Self-Adaptivity for Magnetic Resonance Imaging. Research. 2024;7:0560.DOI:10.34133/research.0560
17. Aydin, K.; Bulu, I.; Guven, K.; Kafesaki, M.; Soukoulis, C. M.; Ozbay, E. Investigation of magnetic resonances for different split ring resonator parameters and designs. New Journal of Physics 2005, 7, 168.DOI 10.1088/1367-2630/7/1/168
18. **Li, B.; Xie, R.; Sun, Z.; et al.** Nonlinear Metamaterials Enhanced Surface Coil Array for Parallel Magnetic Resonance Imaging. Nature Communications **2024,** 15, 7949. https://doi.org/10.1038/s41467-024-52423-1
19. **Freire, M. J.; Lopez, M. A.; Algarin, J. M.; Breuer, F.; Marqués, R.** Image Acceleration in Parallel Magnetic Resonance Imaging by Means of Metamaterial Magnetoinductive Lenses. AIP Advances **2012**, 2, 022136. https://doi.org/10.1063/1.4723675
20. **Mo, Z.; Che, S.; Du, F.; Xiao, E.; Chen, Q.; Li, N.; Jia, S.; Tie, C.; Wu, B.; Zhang, X.; Zheng, H.; Li, Y.** A Near-Field Coupling Array Enables Parallel Imaging and SNR Gain in MRI. Advanced Science **2025**, 12(37), e03481. https://doi.org/10.1002/advs.202503481
21. **Ohliger, M. A.; Sodickson, D. K.** An Introduction to Coil Array Design for Parallel MRI. NMR in Biomedicine **2006,** 19, 300–315. https://doi.org/10.1002/nbm.1046
22. **Dietrich, O.; Raya, J. G.; Reeder, S. B.; Reiser, M. F.; Schoenberg, S. O.** Measurement of Signal-to-Noise Ratios in MR Images: Influence of Multichannel Coils, Parallel Imaging, and Reconstruction Filters. Journal of Magnetic Resonance Imaging **2007,** 26(2), 375–385. https://doi.org/10.1002/jmri.20969
23. **Robson, P. M.; Grant, A. K.; Madhuranthakam, A. J.; Lattanzi, R.; Sodickson, D. K.; McKenzie, C. A.** Comprehensive Quantification of Signal-to-Noise Ratio and g-Factor for Image-Based and k-Space-Based Parallel Imaging Reconstructions. Magnetic Resonance in Medicine **2008,** 60(4), 895–907. https://doi.org/10.1002/mrm.21728
24. **Constantinides, C. D.; Atalar, E.; McVeigh, E. R.** Signal-to-Noise Measurements in Magnitude Images from NMR Phased Arrays. Magnetic Resonance in Medicine **1997,** 38(5), 852–857. https://doi.org/10.1002/mrm.1910380524
25. **Griswold, M. A.; Jakob, P. M.; Heidemann, R. M.; Nittka, M.; Jellus, V.; Wang, J.; Kiefer, B.; Haase, A.** Generalized Autocalibrating Partially Parallel Acquisitions (GRAPPA). Magnetic Resonance in Medicine **2002**, 47(6), 1202–1210. https://doi.org/10.1002/mrm.10171
26. **Deshmane, A.; Gulani, V.; Griswold, M. A.; Seiberlich, N.** Parallel MR Imaging. Journal of Magnetic Resonance Imaging **2012,** 36, 55–72. https://doi.org/10.1002/jmri.23639
27. **Pruessmann, K. P.; Weiger, M.; Scheidegger, M. B.; Boesiger, P.** SENSE: Sensitivity Encoding for Fast MRI. Magnetic Resonance in Medicine **1999**, 42, 952–962.
    https://doi.org/10.1002/(SICI)1522-2594(199911)42:5<952::AID-MRM16>3.0.CO;2-S (Siemens mSENSE is derived from this method.)
28. **Kirilina, E.; Kuehne, A.; et al.** Current Controlled Transmit and Receive Coil Elements (C2ONTAR) for Parallel Acquisition and Parallel Excitation Techniques at High-Field MRI. Magnetic Resonance in Medicine **2010**, 64, 236–248. https://doi.org/10.1002/mrm.22402
29. **Sodickson, D. K.; Manning, W. J.** SMASH: Simultaneous Acquisition of Spatial Harmonics Using Multicoil Arrays. Magnetic Resonance in Medicine **1997**, 38(4), 591–603. https://doi.org/10.1002/mrm.1910380414

**Table S1.** MRI Sequence Parameters for Phantom and In Vivo Human Ocular and Occipital Brain Imaging Using Planar and Bend Antennas

| **Antenna**  **Type** | **Scan Type** | **Sequence Name** | **TR / TE (ms)** | **Flip Angle(α)** | **Vref (V)** | **Bandwidth (Hz/Px)** | **FoV (mm)** | **Spatial Resolution (mm^3^)** | **Slice Orientation** | **Scan Time** | **Total Slices** | **Extra Info** |
| --- | --- | --- | --- | --- | --- | --- | --- | --- | --- | --- | --- | --- |
| Planar | Rectangular Phantom | Actual Flip Angle B₁⁺ mapping (AFI) 3D | 62 / 2.9 (TR_1_=22, TR_2_=102) | 50^0^ | 200 | 500 | 384x384 | 3×3×3 | Sagittal | 33:59 | 128 | Spoiler=4.6, 3mm slice |
| Planar | Head Phantom | Actual Flip Angle B₁⁺ mapping (AFI) 3D | 62 / 2.9 (TR_1_=22, TR_2_=102) | 50^0^ | 200 | 500 | 256x256 | 4×4×4 | Sagittal | 8:29 | 64 | Spoiler=4.6, 4mm slice |
| Planar | Rectangular Phantom | Gradient Echo (GRE) FLASH 2D  T1w | 1000 / 2.4 | 40^0^ | 200 | 334 | 384x384 | 1×1×3 | Axial, Sagittal, Coronal | 6:24 | 80 | 3mm slice |
| Planar | Head Phantom | Gradient Echo (GRE) FLASH 2D  T1w | 800 / 2.19 | 30^0^ | 200 | 332 | 320x320 | 1×1×3 | Axial, Sagittal, Coronal | 4:16 | 48 | 3mm slice |
| Planar | Human | Pre-saturation B_1_^+^Mapping 2D | 2530 / 3.45 | 20^0^ | 125 | 201 | 224x224 | 1×1×3 | Sagittal | 00:48 | 5 | 1 average, 3mm slice |
| Planar | Human | MP2RAGE T1w 3D | 5000 / 2.3 | 5^0^ & 9^0^ | 100 | 248 | 200x200 | 1.3×1.3×1.3 | Sagittal | 12:28 | 144 | 1 average, 1.3mm slice |
| Planar | Human | MPR T1w 3D | 1900 / 2.25 | 9^0^ | 150 | 200 | 269x320 | 1×1×1 | Sagittal | 7:27 | 176 | 1 average, 1.3mm slice, Inversion time=900 ms |
| Bend | Head Phantom | Actual Flip Angle B₁⁺ mapping (AFI) 2D | 60 / 2.9 (TR_1_=20, TR_2_=100) | 50^0^ | 200 | 500 | 256x256 | 4×4×2 | Axial, Sagittal, Coronal | 8:13 | 64 | Spoiler=5, 2mm slice |
| Bend | Head Phantom | Gradient Echo (GRE) FLASH 2D | 800 / 2.19 | 30^0^ | 200 | 332 | 320x320 | 1×1×3 | Axial, Sagittal, Coronal | 4:16 | 48 | 3mm slice |
| Bend | Human  (both eyes) | Pre-saturation B_1_^+^Mapping 2D | 2530/3.18 | 20^0^ | 125 | 201 | 224x224 | 1×1×4 | Axial | 00:48 | 3 | 1 average, 4mm slice |
| Bend | Human  (both eyes) | Turbo Spin Echo (TSE) T2w 2D | 4600 / 84,  4600 / 84,  2500/84, | 100^0^  100^0^  80^0^ | 125 | 260 | 180x180, 192x192,  180x180 | 2×2×2,  1×1×1,  2×2×2 | Sagittal,  Axial,  Coronal | 4:23,  2:23,  2:23 | 20,  6,  20 | 2 averages, 2 & 1mm slice |
| Bend | Human  (single eye) | Gradient Echo (GRE) FLASH 3D T1w | 2526/3.13,  2535/3.15, | 6^0^ | 125 | 300 | 204x208,  208x208 | 0.5×0.5×1 | Axial,  Sagittal | 1:31 | 24 | 2 averages, 1mm slice |
| Bend | Human  (both eyes) | Gradient Echo (GRE) FLASH 3D T1w | 2195/2.82 | 6^0^ | 125 | 300 | 204x208 | 1×1×1 | Axial | 1:19 | 24 | 2 averages, 1mm slice |
| Bend-MTMA | Head Phantom  (MR Thermometry) | PRFS: dual gradient-echo (GRE) 2D | TR= 40/ TE₁ = 2.26 msTE₂ = 6.34 ms | 90^0^ | 205 (10W),  72 (5W) | 300 | 227x280 | 1.5×1.5×3.0 | Axial, Sagittal, Coronal | 30:00 | 44 | 3000 averages |

*Detailed acquisition parameters for all B₁⁺ mapping, anatomical, and MR-thermometry MRI scans across phantom models and human subjects. Each entry specifies coil type, scan type, sequence, timing parameters, transmit voltage, receiver bandwidth, field-of-view, spatial resolution, orientation, total slices, scan time, and additional sequence-specific notes.
